# Full-Length Genome Sequencing and Analysis of Hepatitis B Viruses Isolated from Iraqi Patients

**DOI:** 10.1155/2024/6826495

**Published:** 2024-04-29

**Authors:** Yaseen I. Mamoori, Ibrahim A. Ahmed, Ayhan R. Mahmood, Safaa A. Al-Waysi

**Affiliations:** ^1^Department of Molecular and Medical Biotechnology, College of Biotechnology, Al-Nahrain University, Jadriya, Baghdad 10072, Iraq; ^2^Department of Plant Biotechnology, College of Biotechnology, Al-Nahrain University, Jadriya, Baghdad 10072, Iraq; ^3^Department of Medical Microbiology, College of Medicine, University of Kirkuk, Kirkuk 36001, Iraq; ^4^GIT Teaching Hospital, Medical City, Baghdad 10049, Iraq

## Abstract

Hepatitis B virus (HBV) causes liver diseases (chronic hepatitis, cirrhosis, and hepatocellular carcinoma) and is a leading health problem worldwide. Sequencing of the whole HBV genome provides insight into the virus genotype, subgenotype, serotype, genetic variation, and viral drug resistance. To date, no study has been conducted on the whole genome sequence of HBV obtained from Iraqi patients. Therefore, this is the first study to sequence clinical samples from these patients. Viral genomic DNA was isolated and amplified using five primer sets to amplify five overlapping regions covering the entire HBV genome. The amplicons were sequenced, aligned to a reference sequence, annotated, and submitted to the National Center for Biotechnology Information GenBank database. Sequence analysis showed that the genome size of the tested viral samples was 3,182 bp and belonged to genotype D, subgenotype D1, and serotype ayw2. Missense mutations were found in the four regions (X, PreS1-S, PreC-C, and P) of the tested samples, leading to amino acid substitutions, which were 8.4%, 5.1%, 4.7%, and 4.6%, respectively. These mutations may cause severe liver diseases.

## 1. Introduction

Hepatitis B viral infection is a life-threatening liver infection and a major worldwide health problem caused by the Hepatitis B virus (HBV). This disease is associated with chronic infections that may cause liver cancer and cirrhosis. Approximately 1.5 million people were newly diagnosed with chronic HBV infection in 2019, and approximately 800 deaths from HBV infection occurred worldwide [1].

Antiviral drugs such as interferon (conventional and pegylated interferon) and nucleos(t)ide analogues (RT inhibitors of HBV) such as lamivudine and entecavir have been used to treat HBV infection [2]. These antiviral drugs suppress HBV replication and consequently decrease liver damage. Nonetheless, these antivirals have side effects and do not clear all the viral particles due to the presence of covalently closed circular DNA (cccDNA) in the liver cells [3]. Therefore, there is an urgent need for new antiviral agents to cure HBV infection, which requires a comprehensive understanding of the genome sequence, structure, and diversity of the virus [2].

HBV is an enveloped circular, partially double-stranded DNA virus with a genome size of 3.2 kb. This viral pathogen is a prototype belonging to the Hepadnaviridae family. The viral genome is composed of the following four overlapping open reading frames (ORFs): the PreC-C region encodes the e antigen (HBeAg) and core protein antigen (HBcAg); the P region encodes the DNA polymerase; the S region encodes a surface protein (HBsAg) large (L), medium (M), and small (S); and the X region encodes the X protein [4]. Owing to the lack of viral DNA polymerase proofreading activity, the viral genome is variable and produces a natural replication error rate [5]. Based on the entire genome sequence divergence of the virus (>8%), it can be classified into nine established genotypes (A-I) and one presumed genotype (J), each with several subgenotypes [6] that are distributed geographically and associated with different infection severity [7]. Genotype D is common in India and other countries such as Africa, the Middle East, and Europe. This genotype is associated with 22% of the chronic HBV infections worldwide [8].

Databases of HBV sequences such as NCBI virus and Hepatitis B virus (HBV) databases frequently consist of whole genome sequences of individual viral isolates obtained from Sanger sequencing. In Iraq, studies were conducted to determine the viral genotypes using molecular techniques of different genes or regions of HBV genomes [9, 10]. Nonetheless, there are no reports on the whole genome sequence of HBV isolated from Iraqi patients. Consequently, to our knowledge, this is the first study designed to sequence and analyse the entire viral genome of seven HBV isolates from patients living in Baghdad governate (the capital of Iraq).

## 2. Materials and Methods

### 2.1. Sampling and DNA Extraction

Sera were isolated from the blood samples of seven HBV positive patients (the inclusion criteria include cases recently diagnosed with HBV, untreated with detectable viral load, and duration of infection did not exceed 6 months. Therefore, all included cases are at early/acute stage of infection. The exclusion criteria were patients had no treatment and with no chronic status with no complication; all information about the samples were included in S1, Supplementary Materials). Two-hundred microlitres of serum were used for viral DNA extraction. The extraction protocol was performed using the Viral Nucleic Acid Extraction Kit II (Geneaid, Taiwan) following the manufacturer's instructions. The purified viral DNA was stored at −20°C for further analysis.

### 2.2. Amplification of Viral Genome and Sequencing

Viral genomic DNA was amplified using primers designed by Snapgene software (https://www.snapgene.com), except F3 and F5 primers were obtained from references [11, 12] to amplify five overlapping amplicons that encompassed the whole genome sequence of the virus (Table 1). Amplification of the viral genome was achieved using 1X GoTaq® G2 Green Master Mix (reaction buffer (pH 8.5), 200 *µ*M dATP, 200 *µ*M dGTP, 200 *µ*M dCTP, 200 *µ*M dTTP and 1.5 mM MgCl_2_, (Promega, USA)), 0.4 *µ*M of each forward and reverse primers and 100 ng of templates DNA in a total volume 50 *μ*l of reaction mixture. The cycling rounds consisted of 35 cycles of denaturation at 95°C for 30 s, annealing for 30 s at different temperatures depending on the primer, and extension time at 72°C for 1 min to extend X, PreC-C, and P fragments as well as 1.5 min to extend PreS1-S and P1 fragments. Initial denaturation was performed at 95°C for 2 min, and a final extension was performed at 72°C for 5 min in a thermal cycler (Prime Elite Satellite, Techne, UK). Finally, the amplicons were sent for Sanger sequencing (Macrogen, South Korea).

### 2.3. Genotyping, Subgenotyping, and Serotyping of HBV Samples

The genotypes of the samples were annotated using the annotation algorithm included in the HBV database (HBVdb) https://hbvdb.lyon.inserm.fr/HBVdb/ [5]. The subgenotype was determined using phylogenetic analysis by comparing the samples to other subgenotypes in the NCBI GenBank database and confirmed using the online tool Geno2pheno [hbv] 2.0 https://hbv.geno2pheno.org/index.php. HBV serotypes were determined using HBsAg for each sample in the HBV Serotyper tool [13]. All obtained sequences were annotated using the integrated annotation tool in HBVdb to determine the coding sequences and their translated proteins.

### 2.4. Phylogenetic Tree Construction

The obtained nucleotide sequences were aligned to the reference sequence (NC_003977) in the NCBI GenBank database using the Clustal W method [14] integrated into the SnapGene software. Each sample sequence was aligned to the NCBI database using the Standard Nucleotide Basic Local Alignment Search Tool BLAST (BLASTn, https://blast.ncbi.nlm.nih.gov/Blast.cgi). A total of 100 nucleotide sequences, 7 sequenced HBV isolates, 86 reference genomes retrieved from the NCBI GenBank database of genotype D, and 7 randomly selected sequences of other genotypes A, B, C, E, F, G, and H retrieved from HBVdb were aligned and a phylogenetic tree was constructed using the unweighted pair group method with arithmetic mean (UPGMA) method [15]. The bootstrap consensus tree inferred from 1000 replicates was used to represent the evolutionary history of the analysed taxa [16]. The evolutionary distances were computed using the maximum composite likelihood method [17]. Evolutionary analyses were conducted using the MEGA X software [18]. Another bootstrap consensus phylogenetic tree was created using the neighbour-joining method [19] to determine the subgenotypes of HBV isolates by comparing 7 samples to 93 randomly selected genomes of six subgenotypes (D1–D5 and D7) retrieved from the NCBI GenBank database.

### 2.5. Amino Acid Substitutions

Substitutions in amino acid sequences between the reference sequence (NC_003977) and the samples were determined. First, the entire genome of samples and the reference were divided into four regions (X, C, P, and PreS1-S) and aligned using the SnapGene software. For each region, amino acid locus, amino acid substitution, and substitution percentage among aligned sequences were determined using the Mutation Reporter Tool http://hvdr.bioinf.wits.ac.za/mrt/ [20].

## 3. Results

### 3.1. Whole HBV Genome Amplification and Sequencing

**Figure 1:**
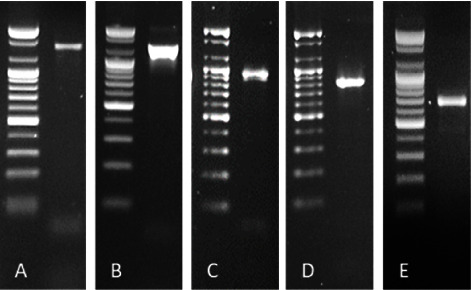
Amplification of five regions (X-region, PreC-C-region, PreS1-S-region, and two P-regions) of HBV genome. (A) P1 region. (B) PreS1-S region. (C) P region. (D) PreC-C region. (E) X region with the molecular sizes 1540, 1265, 941, 826, and 681, respectively. M: 100 bp DNA ladder. Amplicons were run on 1.5% agarose with 5 V/cm for 100 min.

The primers used in this study successfully amplified five overlapping amplicons with 681, 826, 1265, 941, and 1540 bp (Figure 1). Next, the amplicons from the five regions were sequenced and aligned to the reference genome (NC_003977) to obtain the whole genome sequence (S2, Supplementary Materials). The genomes were annotated and submitted to the NCBI GenBank database under accession numbers OM721310–OM721316.

### 3.2. HBV Genotypes and Serotypes

Whole genome sequence analyses demonstrated that all tested samples belonged to genotype D/D1 and were sensitive to viral drugs (S3 and S4, Supplementary Materials). Furthermore, analysis of the amino acid sequences of the samples revealed that amino acids 122, 160, 127, 159, and 140 were R, K, P, G, and T, respectively. Consequently, all HBV samples were ayw2 serotype (S5, Supplementary Materials).

### 3.3. Phylogeny Analysis

**Figure 2:**
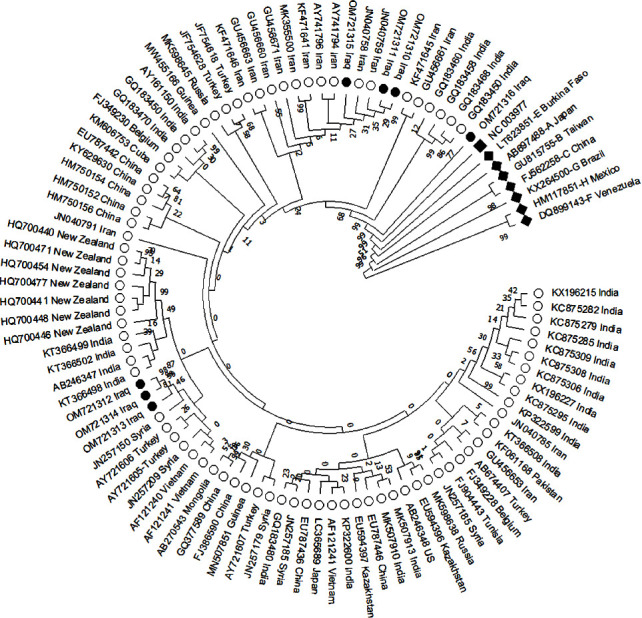
A phylogenetic tree created using the UPGMA method. The bootstrap consensus tree inferred from 1000 replicates. The percentage of replicate trees is shown next to the branches. The evolutionary distances were computed using the maximum composite likelihood method. This analysis involved 100 nucleotide sequences (3182 positions in the final dataset representing the full genome sequences of the HBV isolates). Evolutionary analyses were conducted in the MEGA X program. Filled diamond: HBV genotypes A, B, C, E, F, G, and H; filled circles: HBV Iraqi isolates (genotype D); open circles: HBV isolates from NCBI GenBank database (genotype D).

**Figure 3:**
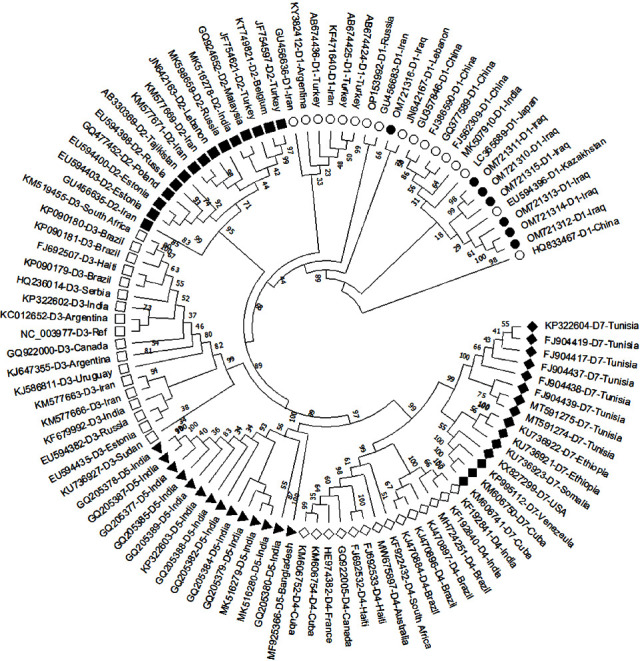
A phylogenetic tree created using the neighbour-joining method. The bootstrap consensus tree inferred from 1000 replicates. The percentage of replicate is shown next to the branches. The evolutionary distances were computed using the maximum composite likelihood method. This analysis involved 100 nucleotide sequences (3182 positions in the final dataset representing the full genome sequences of the HBV isolates). Evolutionary analyses were conducted in the MEGA X program. Open circles: HBV Iraqi isolates (subgenotype D1); filled circles: HBV isolates (subgenotype D1); filled squares: HBV isolates (subgenotype D2); open squares: HBV isolates (subgenotype D3); filled triangle: HBV isolates (subgenotype D5); open diamond: HBV isolates (subgenotype D4); filled diamond: HBV isolates (subgenotype D7).

The phylogenetic tree of 100 HBV genomes, including seven sequenced samples demonstrated that the HBV OM721312 and OM721314 strains are closely related and share a common ancestor with the OM721313 strain, which, in turn, shares a common ancestor with the Syrian HBV strain (JN257150). In addition, OM721310 and OM721311 were closely related, shared a common ancestor with OM721315, and shared a common ancestor with the Iranian strains (AY741794 and AY741796). The OM721316 strain has several mutations, diverges from other Iraqi strains, and shares a common ancestor with the other strains in genotype D (Figure 2). The phylogenetic tree in Figure 3 shows that all HBV Iraq strains (OM721310–OM721316) belonged to sub genotype D1 by clustering together with 18 strains of sub genotype D1 retrieved from the NCBI GenBank database.

### 3.4. Nucleotide Mutations in the Basal Core Promoter Region

Total length of this region is 108 nucleotide (1742–1849 nt). Seven mutations were detected in this region, representing a mutation frequency 6.4% of the total nucleotide (Table 2).

### 3.5. Amino Acid Substitution

Amino acid substitutions of the samples in the PreC-C region were found in ten loci out of 212 aa loci compared to the reference strain, representing a 4.7% substitution frequency. The first substituted aa, G29D, occurs in the precore region, whereas the other is within the core gene (Table 3).

In the X region, substitutions in amino acids (aa) occurred in thirteen aa loci out of 154 aa loci, representing a substitution frequency of 8.4% (Table 4).

Amino acid substitutions of the samples in the PreS1-S region were found in 20 out of 389 aa loci compared to the reference strain, representing a 5.1% substitution frequency (Table 5).

In the P region, aa substitutions of the samples with the reference strain were found in 39 out of 832 aa loci, representing a 4.6% substitution frequency. Eight mutations occurred in the terminal protein (TP) domain. In the spacer domain, thirteen mutations were found in this region. Sixteen mutations were present in the reverse transcriptase (RT) domain. One mutation in this domain (G961A) causing aa substitution (I278V) may lead to low viral replication and hence may lead to the progression of liver disease. In the RNase H domain, two mutations were present in which single highly variable location in which serine was substituted with leucine, arginine, and glutamine (S30/L/R/Q) as shown in Table 6.

## 4. Discussion

Determining the entire HBV genome is essential for clinical diagnosis and treatment by revealing the virus's genetic variations, genotype, subgenotype, serotype, and resistance to drugs. Many studies have reported that mutations in the basal core promoter and S gene are associated with variable antigen expression levels that affect immunogenicity. This variation correlates with liver disease progression and risk of developing hepatocellular carcinoma (HCC) [34]. Challenges in sequencing the HBV whole genome led to the use of many primers and nested PCR to amplify whole genome sequences. However, only one or a few samples were sequenced completely [35]. In this study, the complete genomes of seven HBV isolates were successfully amplified using five primer sets and conventional PCR. Although we attempted to amplify and sequence twenty HPV samples during this study, amplification failed for thirteen samples due to the limited viral load [36]. According to our results, a viral load of ≥50000 IU/ml can be used for amplification and sequencing.

Genotypes are associated with the severity of liver disease and resistance to antiviral drugs [37]. Patients infected with genotype D have a higher likelihood of developing liver cirrhosis and HCC compared to those infected with other genotypes [38]. In this study, all the viral samples tested belonged to genotype D which was inferred using the HBVdb algorithm.

Genotype D contributes to approximately 22.1% of chronic HBV infections worldwide [8]. In Iraq, one study demonstrated that out of 134 tested samples, 99.22% belonged to genotype D [39]. Several studies from the countries surrounding Iraq showed the predominance of genotype D. Reports from Jordan, Iran, and Saudi Arabia demonstrated the prevalence of genotype D compared to other HBV genotypes, which were 100%, 95%, and 90%, respectively [40, 41]. In addition, the current study revealed that all HBV samples relevant to serotype ayw2 depend on HBsAg amino acids present at specific loci [13]. Other studies conducted in Iran and Turkey have shown the dominance of this serotype among the tested HBV samples [40, 42].

Phylogenetic analysis revealed that three strains (OM721312, OM721313, and OM721314) were closely related to the Syrian strain (JN257150). The other three strains (OM721310, OM721311, and OM721315) clustered with two Iranian strains (JN040758 and JN040759). After the Gulf War in 2003, cross-border farming occurred, which could explain the relatedness of our isolates with the Syrian and Iranian isolates. However, strain OM721316 contains more mutations than the other isolates, appears to be clustered alone, and shares a common ancestor with other genotype D isolates.

Mutations in both the basal core promoter (BCP) and precore (PC) regions lead to the reduction and cessation of HBeAg production. Consequently, this may lead to the virus escaping from the immune system, which increases the risk of liver diseases [43]. Five mutations (A1762T, C1766T, T1768A, C1773T, and C1799G) were found in local isolates and might be associated with liver cirrhosis [23], one mutation (A1752C) with no reported effect [21] whereas only G1757A mutation has a protective action against liver disease [22]. In the PC region, G1899A mutation results in G29D substitution that might confer a higher risk of HCC [24].

Studies have shown the importance of specific mutations in the core gene coding for a protein with 183 residues (HBcAg), which enhances the cytotoxic T lymphocyte immune response [25]. As a result, mutations in this region promote the viral invasion of the host immune response [26]. In this study, nine-point mutations in this region were detected in HBV isolates. Two research groups have reported five mutations [25, 26], namely, A2092T, G2129C, G2138A, A2174C, and T2363A, which correspond to E64D, E77Q, A80T, N92H, and S155T aa substitutions, respectively. The E77Q and A80T substitutions are located within the B-cell epitope recognition site, whereas E64D is located within the T-cell epitopes, which may be related to the severity of liver disease in CHB patients [26].

Several studies have demonstrated the importance of mutations in the X region coding for the HBx protein, which may increase tumorigenesis and liver cell death [27]. In this study, five mutations were observed (T1464C, A1479T, C1653T, A1752C, and A1762T) corresponding to the S31P, T36S, H94Y, I127L, and K130M aa substitutions, respectively [28]. They not only affect the HBx open reading frame (ORF) but also cis-acting overlapped regions, such as BCP, enhancer II, and microRNA-binding regions, which may be related to the severity of liver disease [28].

Many mutations in the S region have been detected in previous studies; most of them had aa substitutions due to point mutations; however, nucleotide deletions were also observed. Although some mutations were identified in the pre-S1 and pre-S2 regions, most were located within the S region. Certain mutations in this region may be related to primary clinical or biological events, such as failure to detect HBsAg immune escape and development of HCC [44]. Twenty-point mutations were identified in this study. The significance of these mutations could not be estimated because it was beyond the scope of this study, except 4 mutations that had been reported in the literature, namely, C105T, A162G, G225A, and T678C, which corresponds to the A39V, N3S, R24K, and M125T aa substitutions. These mutations are related to immune escape and HCC [29–32].

HBV polymerase contains the following four domains: a TP, essential for initiation of DNA synthesis; a spacer domain; an RT domain, responsible for polymerase activity; and an RNase H domain, essential for template RNA removal [45]. Thirty-nine locations in the polymerase region had point mutations. Antiviral drug resistance was also attributed to mutations in the RT domain, which comprises 344 aa in genotype D and between 131 and 1161 nucleotides. This domain was further divided into seven conserved boxes from A to G, which are essential for the catalytic activity of the polymerase enzyme. Point mutations cause aa substitutions and consequently antiviral drug resistance. These mutations include A181T and M204V/I/S, which are associated with resistance to lamivudine, while A181T/V and N236T are linked to resistance to adefovir dipivoxil. Moreover, S202G/C, M250V, and M204I may mediate resistance to telbivudine [46]. Mutations in the polymerase region of viral isolates in this study were compared to those in previous studies. Only one mutation (G961A) which leads to I278V aa substitution was reported in a previous study, which may cause low viral replication and hence could lead to the development of liver disease [33].

In this study, the impact of HBV gnome mutations seems not to be correlated with the disease severity or complications and it is more likely to be correlated with the viral load status where isolates with high viral loads ended up with mutations in many sites, particularly isolate no. 7, which ended up with highest mutation rates.

This is the first report of whole genome sequencing of HBV isolated from Iraqi patients with acute hepatitis. However, more samples should be sequenced from different regions in Iraq using next generation sequencing (NGS), a comprehensive study of the virus recombination and its epidemiology, and the novel-uncharacterized missense mutations in the viral genomes and their effect will need to be addressed in the future.

## 5. Conclusions

In conclusion, the genome of HBV samples isolated from patients living in Baghdad-Iraq were sequenced and analysed using online tools, demonstrating that all samples belonged to genotype D, subgenotype D1, and serotype ayw2 and might be sensitive to antiviral drugs. Several mutations resulted nucleotide changes in BCP and aa substitutions in all regions (X, PreC-C, PreS1-S, and P). Seven nucleotide mutations were found in the BCP region, which may be involved in HCC. Eight mutations within the PreC-C region may be linked to the lack of HBeAg production, leading to a high risk of HCC. Nine mutations were located within the X region, which may be related to the severity of liver disease. In the PreS1-S region, three mutations had been reported in the literature related to virus immune escape and HCC. Within the P region, 41-point mutations were detected; however, only one mutation may be related to low viral replication and could lead to liver disease.

## Figures and Tables

**Table 1:** Primers used for amplification of the whole HBV genome.

Primer	Sequence (5′–3′)	Genome Location^*∗*1^	Region	Ta^*∗*2^ (°C)	Amplicon size (bp)	Reference
F1	CATACTGCGGAACTCCTAGC	1267–1286	X	61	681	Current study
R1	AGTAACTCCACAGTAGCTCC	1947–1928				
F2	GAGGCATACTTCAAAGACTG	1698–1717	PreC-C	59	826	
R2	GAGGGTTAAAGACAGGTACAG	2523–2503				
F3	GGAAGAGAAACGGTCATAGAG	2231–2251	P	60	941	
R3	GGCCTGAGGATGAGTGTTTC	3171–3152				

F4	ATGGGGCAGAATCTTTCC	2848–2865	PreS1-S	59	1265	[11]
R4	CTGTATGATGTGATCTTGTGGC	930–909				Current study

F5	ATGGAGAACATCACATCAGG	155–174	P1	60	1540	[12]
R5	GTCGGTCGTTGACATTACAG	1694–1675				Current study

^*∗*1^Consensus DNA sequence created from aligning of 1094 genome sequences of HBV genotype D isolates found in the HBVdb, ^*∗*2^Ta: annealing temperature.

**Table 2:** Mutations in the BCP region.

Region	Mutation	Mutation possible effect	Reference
BCP (1742–1849 nt)	**A1752C**	U	[21]
	**G1757A**	Protective	[22]
	**A1762T**	HCC	[23]
	**C1766T**	HCC	[23]
	**T1768A**	HCC	[23]
	**C1773T**	HCC	[23]
	**C1799G**	HCC	[23]

Bold font mutation: indicated known referenced mutation, HCC: hepatocellular carcinoma, U: uncharacterized effect.

**Table 3:** Mutations and amino acid substitutions in the PreC-C region.

Region	Mutation	aa substitution	Mutation possible effect	Reference
PreC (1814–1900 nt)	**G1899A**	**G29D**	HCC	[24]
Core (1901–2452 nt)	**G1985C**	**D29H**	U	[25]
	**A2092T**	**E64D**	CHB	[26]
	**T2121G**	**V74G**	HCC	[26]
	**G2129C**	**E77Q**	CHB	[26]
	**G2138A**	**A80T**	CHB	[26]
	G2140A	A80I	U	Current study
	A2174C	N92H	U	Current study
	**C2289A**	**P130Q**	HCC	[26]
	**T2363A**	**S155T**	U	[26]

Bold font mutation: indicated known referenced mutation, aa: amino acid, CHB: chronic hepatitis, HCC: hepatocellular carcinoma, U: uncharacterized effect.

**Table 4:** Mutations and amino acid substitutions in the X region.

Region	Mutation	aa substitution	Mutation possible effect	Reference
X (1374–1838 nt)	**T1449C**	**C26R**	CHB, LC	[27]
	**T1464C**	**S31P**	CHB, LC	[27]
	T1470C	S33P	U	Current study
	**A1479T**	**T36S**	LC	[28]
	T1494A	S41T	U	Current study
	**C1509T**	**P46S**	CHB, LC	[27]
	A1634C	Q87H	U	Current study
	A1637C	I88F	U	Current study
	**C1653T**	**H94Y**	CHB, HCC	[27]
	**C1678T**	**A102V**	CHB, LC	[27]
	**A1752C**	**I127L**	CHB, LC	[27]
	**A1762T**	**K130M**	CHB, LC	[27]
	**T1768A**	**F132Y**	LC	[28]

Bold font mutation: indicated known referenced mutation, aa: amino acid, CHB: chronic hepatitis, HCC: hepatocellular carcinoma, LC: liver cirrhosis, U: uncharacterized effect.

**Table 5:** Mutations and amino acid substitutions in the PreS1-S region.

Region	Mutation	aa substitution	Mutation possible effect	Reference
PreS1 (2848–3171 nt)	A2965C	T40P	U	Current study
	G3082A	A79T	U	Current study
	T3100G	S85A	U	Current study
	A3154G	N103D	U	Current study

PreS2 (3172−154 nt)	T96C	L36P	U	Current study
	**C105T**	**A39V**	HCC	[29]
	C111A	P41H	U	Current study
	T113A	L43I	U	Current study
	A141G	D51G	U	Current study

S (155–835 nt)	**A162G**	**N3S**	U	[30]
	G183A	G10R	U	Current study
	T213C	F20S	U	Current study
	**G225A**	**R24K**	HCC	[31]
	C242A	Q30K	U	Current study
	T279G	L42R	U	Current study
	**T528C**	**M125T**	IE	[32]
	A533C	T127P	U	Current study
	T678C	L175S	U	Current study
	C732T	S193L	U	Current study
	C762G	P203R	U	Current study

Bold font mutation: indicated known referenced mutation, aa: amino acid, HCC: hepatocellular carcinoma, IE: immune escape, U: uncharacterized effect.

**Table 6:** Mutations and amino acid substitutions in the P region.

Region	Mutation	aa substitution	Mutation effect	Reference
TP domain (2307–2837 nt)	A2326G	H7R	U	Current study
	C2354G	D16E	U	Current study
	G2565A	D87N	U	Current study
	A2588C	Q94H	U	Current study
	T2660G	K118N	U	Current study
	G2661A	V119I	U	Current study
	A2797G	H164R	U	Current study
	G2812A	C169Y	U	Current study

Spacer domain (2838–129 nt)	G2840A	D178E	U	Current study
	C2928T	R31C	U	Current study
	A2965C	H43P	U	Current study
	C2976T	R47C	U	Current study
	T3012A	F59I	U	Current study
	T3075C	F80L	U	Current study
	G3082A	S82N	U	Current study
	T3100G	L88R	U	Current study
	C3102T	H89Y	U	Current study
	G3135T	A100S	U	Current study
	A3154G	K106R	U	Current study
	T1C	F116L	U	Current study
	T113A	L153H	U	Current study

RT domain (130–1161 nt)	G182A	R18K	U	Current study
	T190G	S21A	U	Current study
	C242A	A38E	U	Current study
	C493T	L122F	U	Current study
	C493G	L122V	U	Current study
	A499C	N124H	U	Current study
	A511C	T128P	U	Current study
	A514T	M129L	U	Current study
	C518A	P130Q	U	Current study
	A520G	D131N	U	Current study
	A533C	Y135S	U	Current study
	C787A	L220I	U	Current study
	A871C	N249H	U	Current study
	A918T	E264D	U	Current study
	**G961A**	**I278V**	LVR	[33]
	C1084A	Q319L	U	Current study

RNase H domain (1162–1620 nt)	T1249C C1250T	S30L	U	Current study
	T1249C C1250G	S30R	U	Current study
	T1249C C1250A	S30Q	U	Current study
	A1324C C1326T	I55L	U	Current study

Bold font mutation: indicated known referenced mutation, aa: amino acid, LVR: low viral replication, U: uncharacterized effect.

## Data Availability

The data used to support the findings of this study are included within the article. Raw data are available from the corresponding author upon request.

## Supplementary Materials

Supplementary Materials The metadata of HBV samples is shown in S1, Supplementary Materials, as a PDF file. Alignment of the obtained nucleotide sequences (OM721310–OM721316) to the reference sequence (NC_003977) in the NCBI GenBank database using the Clustal W method integrated into the SnapGene software is shown in S2, Supplementary Materials, as a PDF file. Determination of genotypes of HBV isolates (OM721310–OM721316) using the annotation algorithm in the HBVdb is shown in S3, Supplementary Materials, as a PDF file. Determination of genotypes, subgenotypes of HBV isolates (OM721310–OM721316), and their sensitivity to antiviral drugs using Geno2pheno are shown in S4, Supplementary Materials, as PDF files. Serotype determination of HBV isolates (OM721310–OM721316) using the HBV Serotyper tool is shown in S5, Supplementary Materials, as a PDF file.
