# Supplementary material for: Full-Length Genome Sequencing and Analysis of Hepatitis B Viruses Isolated from Iraqi Patients
Source: Int J Microbiol. 2024 Apr 29;2024:6826495. doi: 10.1155/2024/6826495 (PMC11074772; doi:10.1155/2024/6826495)
Supplement: Supplementary Materials — The metadata of HBV samples is shown in S1, Supplementary Materials, as a PDF file. Alignment of the obtained nucleotide sequences (OM721310–OM721316) to the reference sequence (NC_003977) in the NCBI GenBank database using the Clustal W method integrated into the SnapGene software is shown in S2, Supplementary Materials, as a PDF file. Determination of genotypes of HBV isolates (OM721310–OM721316) using the annotation algorithm in the HBVdb is shown in S3, Supplementary Materials, as a PDF file. Determination of genotypes, subgenotypes of HBV isolates (OM721310–OM721316), and their sensitivity to antiviral drugs using Geno2pheno are shown in S4, Supplementary Materials, as PDF files. Serotype determination of HBV isolates (OM721310–OM721316) using the HBV Serotyper tool is shown in S5, Supplementary Materials, as a PDF file. [file 6826495.f1.zip › S1.Metadata of HBV samples.pdf]

S1. Metadata of HBV samples

Sample No.	Gender	Viral load (IU/ml)	Complication	Location of samples
1	Female	1401000	Acute hepatitis	Baghdad
2	Female	4582000	Acute hepatitis	Baghdad
3	Male	1636500	Acute hepatitis	Baghdad
4	Female	644100	Acute hepatitis	Baghdad
5	Male	4772400	Acute hepatitis	Baghdad
6	Male	6287000	Acute hepatitis	Baghdad
7	Male	9527000	Acute hepatitis	Baghdad
