# Supplementary material for: Full-Length Genome Sequencing and Analysis of Hepatitis B Viruses Isolated from Iraqi Patients
Source: Int J Microbiol. 2024 Apr 29;2024:6826495. doi: 10.1155/2024/6826495 (PMC11074772; doi:10.1155/2024/6826495)
Supplement: Supplementary Materials — The metadata of HBV samples is shown in S1, Supplementary Materials, as a PDF file. Alignment of the obtained nucleotide sequences (OM721310–OM721316) to the reference sequence (NC_003977) in the NCBI GenBank database using the Clustal W method integrated into the SnapGene software is shown in S2, Supplementary Materials, as a PDF file. Determination of genotypes of HBV isolates (OM721310–OM721316) using the annotation algorithm in the HBVdb is shown in S3, Supplementary Materials, as a PDF file. Determination of genotypes, subgenotypes of HBV isolates (OM721310–OM721316), and their sensitivity to antiviral drugs using Geno2pheno are shown in S4, Supplementary Materials, as PDF files. Serotype determination of HBV isolates (OM721310–OM721316) using the HBV Serotyper tool is shown in S5, Supplementary Materials, as a PDF file. [file 6826495.f1.zip › S2. Alignment of the obtained nucleotide sequences (OM721310-OM721316) to the reference sequence (NC_003977).pdf]

```
           CTCCACAACCTTCCACCAAACTCTGCAAGATCCCAGAGTGAGAGGCCTGTATTTCCCTGCTGGTGGCTCCAGTTCAGGAA
NC 003977  T...............................................................................  80
OM721310   ................................................................................  80
OM721311   ................................................................................  80
OM721312   ................................................................................  80
OM721313   ................................................................................  80
OM721314   ................................................................................  80
OM721315   ................................................................................  80
OM721316   ................................................................................  80

           CAGTAAACCCTGTTCCGACTACTGTCTCTCACATATCGTCAATCTTCTCGAGGATTGGGGACCCTGCGCTGAACATGGAG
NC 003977  ...............T........C.....C.T...............................................  160
OM721310   ................................................................................  160
OM721311   ................................................................................  160
OM721312   ................................................................................  160
OM721313   ................................................................................  160
OM721314   ................................................................................  160
OM721315   ................................................................................  160
OM721316   ............................................................G...................  160

           AACATCACATCAGGATTCCTAGGACCCCTGCTCGTGTTACAGGCGGGGTTTTTCTTGTTGACAAGAATCCTCACAATACC
NC 003977  .............................T..................................................  240
OM721310   .G..............................................................................  240
OM721311   .G..............................................................................  240
OM721312   ................................................................................  240
OM721313   ................................................................................  240
OM721314   ................................................................................  240
OM721315   ................................................................................  240
OM721316   .....................A..............................C...........A...............  240

           GCAGAGTCTAGACTCGTGGTGGACTTCTCTCAATTTTCTAGGGGGAACTACCGTGTGTCTTGGCCAAAATTCGCAGTCCC
NC 003977  ................................................................................  320
OM721310   ................................................................................  320
OM721311   ......................................G.........................................  320
OM721312   ................................................................................  320
OM721313   ................................................................................  320
OM721314   ................................................................................  320
OM721315   ................................................................................  320
OM721316   .A..............................................................................  320

           CAACCTCCAATCACTCACCAACCTCCTGTCCTCCAACTTGTCCTGGTTATCGCTGGATGTGTCTGCGGCGTTTTATCATC
NC 003977  .........................T......................................................  400
OM721310   ................................................................................  400
OM721311   ................................................................................  400
OM721312   ................................................................................  400
OM721313   ................................................................................  400
OM721314   ................................................................................  400
OM721315   ................................................................................  400
OM721316   ................................................................................  400
```

```
          TTCCTCTTCATCCTGCTGCTATGCCTCATCTTCTTGTTGGTTCTTCTGGACTATCAAGGTATGTTGCCCGTTTGTCCTCT
NC 003977 ................................................................................ 480
OM721310  ................................................................................ 480
OM721311  ................................................................................ 480
OM721312  ................................................................................ 480
OM721313  ................................................................................ 480
OM721314  ................................................................................ 480
OM721315  ................................................................................ 480
OM721316  ................................................................................ 480

          AATTCCAGGATCTTCAACCACCAGCACGGGACCATGCAGAACCTGCACGACTCCTGCTCAAGGAACCTCTATGTATCCCT
NC 003977 ............C.....A..................C.G.......T....A........................... 560
OM721310  ................................................................................ 560
OM721311  ................................................................................ 560
OM721312  ................................................................................ 560
OM721313  ................................................................................ 560
OM721314  ................................................................................ 560
OM721315  ............G.................C................................................. 560
OM721316  .................................C.....G........................................ 560

          CCTGTTGCTGTACCAAACCTTCGGACGGAAATTGCACCTGTATTCCCATCCCATCATCCTGGGCTTTCGGAAAATTCCTA
NC 003977 ................................................................................ 640
OM721310  ................................................................................ 640
OM721311  ................................................................................ 640
OM721312  ....C........................................................................... 640
OM721313  ....C........................................................................... 640
OM721314  ....C........................................................................... 640
OM721315  ................................................................................ 640
OM721316  ................................................................................ 640

          TGGGAGTGGGCCTCAGCCCGTTTCTCCTGGCTCAGTTTACTAGTGCCATTTGTTCAGTGGTTCGTAGGGCTTTCCCCCAC
NC 003977 ................................................................................ 720
OM721310  ................................................................................ 720
OM721311  .....................................C.......................................... 720
OM721312  ................................................................................ 720
OM721313  ................................................................................ 720
OM721314  ................................................................................ 720
OM721315  .....................................C.......................................... 720
OM721316  ................................................................................ 720

          TGTTTGGCTTTCAGTTATATGGATGATGTGGTATTGGGGGCCAAGTCTGTACAGCATCTTGAGTCCCTTTTTACCGCTGT
NC 003977 ................................................................................ 800
OM721310  ................................................................................ 800
OM721311  ................................................................................ 800
OM721312  ..................................................................A............. 800
OM721313  ..................................................................A............. 800
OM721314  ..................................................................A............. 800
OM721315  ................................................................................ 800
OM721316  ...........T.............................G...................................... 800
```

```
           TACCAATTTTCTTTTGTCTTTGGGTATACATTTAAACCCTAACAAAACAAAAAGATGGGGTTACTCTTTACATTTCATGG
NC 003977  ...................................................G...............C..A....T....  880
OM721310   ............................................................G.....C.............  880
OM721311   ............................................................G.....C.............  880
OM721312   ...................C............................................................  880
OM721313   ...................C............................................................  880
OM721314   ...................C............................................................  880
OM721315   ............................................................G...................  880
OM721316   ....................................T...........................................  880

           GCTATGTCATTGGATGTTATGGGTCATTGCCACAAGATCACATCATACAGAAAATCAAAGAATGTTTTAGAAAACTTCCT
NC 003977  .T.......................C...........A...........A..............................  960
OM721310   ......................T.........................................................  960
OM721311   ......................T.........................................................  960
OM721312   ................................................................................  960
OM721313   ................................................................................  960
OM721314   ................................................................................  960
OM721315   ......................T.........................................................  960
OM721316   .........................C.......................A....................G.........  960

           GTTAACAGGCCTATTGATTGGAAAGTCTGTCAACGTATTGTGGGTCTTTTGGGTTTTGCTGCCCCTTTTACACAATGTGG
NC 003977  A.........................A........A............................................  1040
OM721310   ................................................................................  1040
OM721311   ................................................................................  1040
OM721312   ................................................................................  1040
OM721313   ................................................................................  1040
OM721314   ................................................................................  1040
OM721315   A.........................................................................G..C..  1040
OM721316   ................................................................................  1040

           TTATCCTGCTTTAATGCCTTTGTATGCATGTATTCAATCTAAGCAGGCTTTCACTTTCTCGCCAACTTACAAGGCCTTTC
NC 003977  .........G..G.....T.............................................................  1120
OM721310   ..................T.............................................................  1120
OM721311   ..................T.............................................................  1120
OM721312   ..................C.........................T...................................  1120
OM721313   ..................C.........................T...................................  1120
OM721314   ..................C.........................T...................................  1120
OM721315   ..................T..A.....C....................................................  1120
OM721316   ..................C.............................................................  1120

           TGTGTAAACAATACCTGAACCTTTACCCCGTTGCCCGGCAACGGCCAGGTCTGTGCCAAGTGTTTGCTGACGCAACCCCC
NC 003977  ................................................................................  1200
OM721310   ................................................................................  1200
OM721311   ................................................................................  1200
OM721312   ................................................................................  1200
OM721313   ................................................................................  1200
OM721314   ................................................................................  1200
OM721315   ................................................................................  1200
OM721316   ................................................................................  1200
```

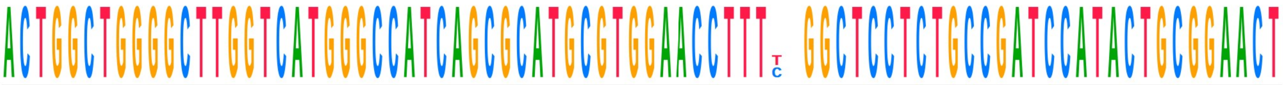

```
NC 003977  ................................................TC..............................  1280
OM721310   ................................................CT..............................  1280
OM721311   ................................................CA..............................  1280
OM721312   ................................................TC..............................  1280
OM721313   ................................................TC..............................  1280
OM721314   ................................................TC..............................  1280
OM721315   ................................................CG..............................  1280
OM721316   ................................................CG..............................  1280
```

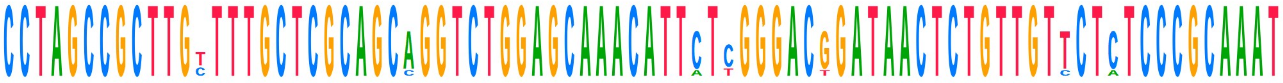

```
NC 003977  ...........................................A.......T..............C..A..........  1360
OM721310   ............C................................T..................................  1360
OM721311   ............C................................T..................................  1360
OM721312   ................................................................................  1360
OM721313   ................................................................................  1360
OM721314   ................................................................................  1360
OM721315   ............C............C......................................................  1360
OM721316   ................................................................................  1360
```

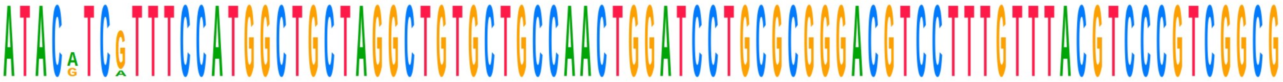

```
NC 003977  ................................................................................  1440
OM721310   .......A........................................................................  1440
OM721311   ................................................................................  1440
OM721312   ....G...........................................................................  1440
OM721313   ....G...........................................................................  1440
OM721314   ....G...........................................................................  1440
OM721315   ................................................................................  1440
OM721316   ................................................................................  1440
```

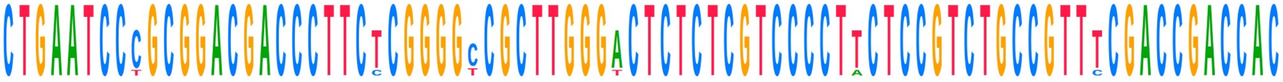

```
NC 003977  ........T....................T......................................C...........  1520
OM721310   ................................................................................  1520
OM721311   ................................................................................  1520
OM721312   ................................................................................  1520
OM721313   ................................................................................  1520
OM721314   ................................................................................  1520
OM721315   ......................................T.........................................  1520
OM721316   .......................C.....T.......................A..........................  1520
```

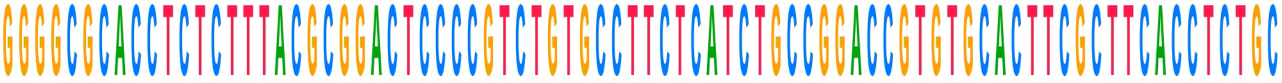

```
NC 003977  ................................................................................  1600
OM721310   ................................................................................  1600
OM721311   ................................................................................  1600
OM721312   ................................................................................  1600
OM721313   ................................................................................  1600
OM721314   ................................................................................  1600
OM721315   ................................................................................  1600
OM721316   ................................................................................  1600
```

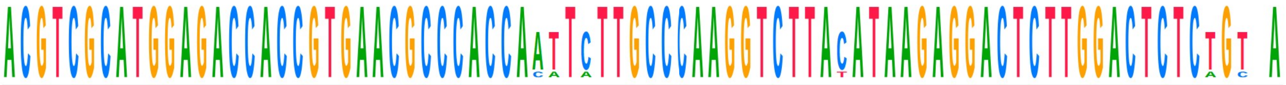

```
NC 003977  ..................................A.A......................................A.CA.  1680
OM721310   ..............................................................................A.  1680
OM721311   ..............................................................................A.  1680
OM721312   ..............................................................................G.  1680
OM721313   ..............................................................................G.  1680
OM721314   ..............................................................................G.  1680
OM721315   ..............................................................................C.  1680
OM721316   .................................C..................T.........................A.  1680
```

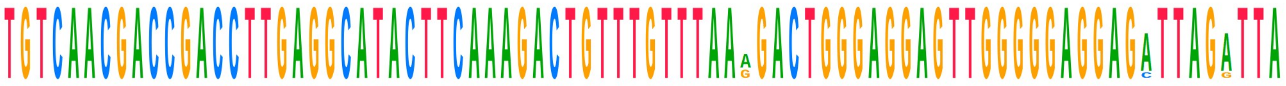

```
NC 003977  ............................................................................G...  1760
OM721310   ..............................................G.................................  1760
OM721311   ..............................................G.................................  1760
OM721312   ................................................................................  1760
OM721313   ................................................................................  1760
OM721314   ................................................................................  1760
OM721315   ..............................................G........................C........  1760
OM721316   ................................................................................  1760
```

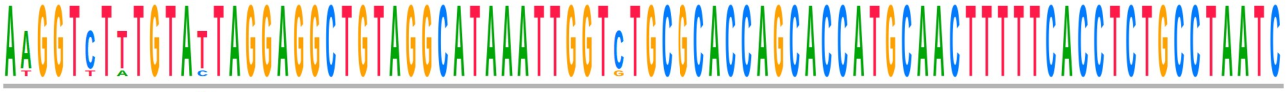

```
NC 003977  ............C...................................................................  1840
OM721310   ................................................................................  1840
OM721311   ................................................................................  1840
OM721312   ................................................................................  1840
OM721313   ................................................................................  1840
OM721314   ................................................................................  1840
OM721315   ................................................................................  1840
OM721316   .T...T.A..............................G.........................................  1840
```

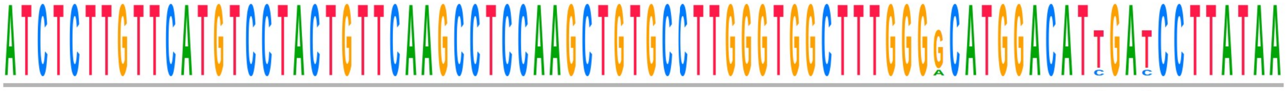

```
NC 003977  ....................................................................C..C........  1920
OM721310   ................................................................................  1920
OM721311   ................................................................................  1920
OM721312   ................................................................................  1920
OM721313   ................................................................................  1920
OM721314   ................................................................................  1920
OM721315   ................................................................................  1920
OM721316   ..........................................................A.....................  1920
```

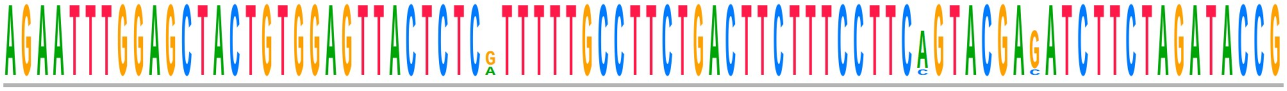

```
NC 003977  ................................................................................  2000
OM721310   ................................................................................  2000
OM721311   ...............................................................C................  2000
OM721312   ..............................A.................................................  2000
OM721313   ..............................A.................................................  2000
OM721314   ..............................A.................................................  2000
OM721315   ................................................................................  2000
OM721316   .........................................................C......................  2000
```

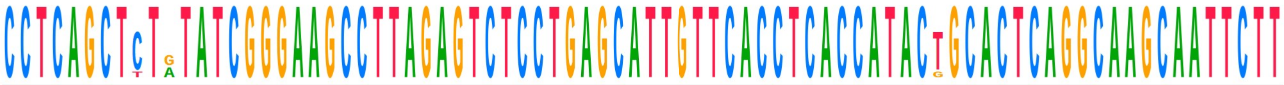

```
NC 003977  ..........G.....................................................................  2080
OM721310   ..........G.....................................................................  2080
OM721311   ..........G.....................................................................  2080
OM721312   ..........A.....................................................................  2080
OM721313   ..........A.....................................................................  2080
OM721314   ..........A.....................................................................  2080
OM721315   ..........G.....................................................................  2080
OM721316   ........T.A...............................................G.....................  2080
```

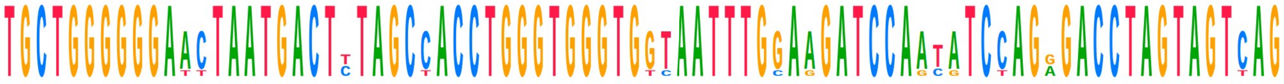

```
NC 003977  .....................C....T.............T................GCG..T..A..............  2160
OM721310   .....................C...........................................A..............  2160
OM721311   .....................C...........................................A..............  2160
OM721312   .....................T...........................................G..............  2160
OM721313   .....................T...................C.......................G..............  2160
OM721314   .....................T...........................................G..............  2160
OM721315   .....................T...........................................A..............  2160
OM721316   ...........TT........C..........................C.G.......C......G...........T..  2160
```

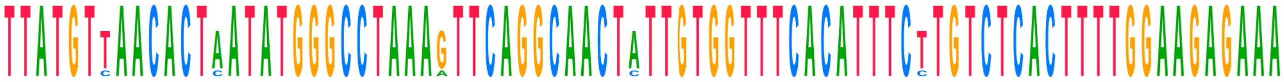

```
NC 003977  ......C................................C........................................  2240
OM721310   ................................................................................  2240
OM721311   ................................................................................  2240
OM721312   ................................................................................  2240
OM721313   ................................................................................  2240
OM721314   ................................................................................  2240
OM721315   ................................................................................  2240
OM721316   .............C.............A.............................C......................  2240
```

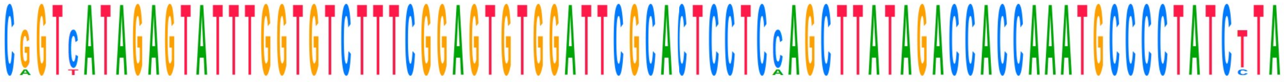

```
NC 003977  .A..T........................................................................C..  2320
OM721310   ................................................................................  2320
OM721311   ................................................................................  2320
OM721312   ................................................................................  2320
OM721313   ................................................................................  2320
OM721314   ................................................................................  2320
OM721315   ................................................................................  2320
OM721316   ................................................A...............................  2320
```

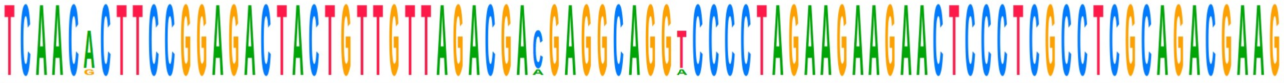

```
NC 003977  ................................................................................  2400
OM721310   ................................................................................  2400
OM721311   ................................................................................  2400
OM721312   ................................................................................  2400
OM721313   ................................................................................  2400
OM721314   ................................................................................  2400
OM721315   ................................................................................  2400
OM721316   .....G...........................A........A.....................................  2400
```

```
           GTCTCAATCGCCGCGTCGCAGAAGATCTCAATCTCGGGAATCTCAATGTTAGTATTCCTTGGACTCATAAGGTGGGAAAC
NC 003977  ............................................................................G...  2480
OM721310   ................................................................................  2480
OM721311   ................................................................................  2480
OM721312   ................................................................................  2480
OM721313   ................................................................................  2480
OM721314   ................................................................................  2480
OM721315   ................................................................................  2480
OM721316   ................................................................................  2480

           TTTACGGGGCTTTATTCTTCTACTGTTCCTGTCTTTAACCCTCATTGGAAAACACCCTCTTTTCCTAATATACATTTACA
NC 003977  .....T....................A...........T.................A.......................  2560
OM721310   ..............C.................................................................  2560
OM721311   ..............C.................................................................  2560
OM721312   .................................................................C........C.....  2560
OM721313   .................................................................C........C.....  2560
OM721314   .................................................................C........C.....  2560
OM721315   ..............C.........................................T.......................  2560
OM721316   ..........................G.....................................................  2560

           CCAAGACATTATCAAAAAATGTGAACA-TTTGTAGGCCCACTCACAGTCAATGAGAAAAGAAGACTGCAATTGATTATGC
NC 003977  ...........................G....................T...............T...............  2640
OM721310   ........................................................................A.......  2640
OM721311   ........................................................................A.......  2640
OM721312   ...........................T....................................................  2640
OM721313   ................................................................................  2640
OM721314   ...........................T....................................................  2640
OM721315   ....A................................................................G..........  2640
OM721316   ......T...................................T.....................................  2640

           CTGCTAGGTTTTATCCAAATGTTACCAAATATTTGCCATTGGATAAGGGTATTAAACCTTATTATCCAGAACATCTAGTT
NC 003977  ....C..............G..............A.............................................  2720
OM721310   ...................TA............................C........C.....................  2720
OM721311   ...................TA............................C........C.....................  2720
OM721312   ..........C........G............................................................  2720
OM721313   ..........C........G............................................................  2720
OM721314   ..........C........G............................................................  2720
OM721315   ...................T....................A........C..............................  2720
OM721316   ...................T............................................................  2720

           AATCATTACTTCCAAACCAGACATTATTTACACACTCTATGGAAGGCGGGTATATTATATAAGAGAGAAACAACACATAG
NC 003977  .................T.....C........................................................  2800
OM721310   ................................................................................  2800
OM721311   ................................................................................  2800
OM721312   ................................................................................  2800
OM721313   ................................................................................  2800
OM721314   ................................................................................  2800
OM721315   .......................................................................T........  2800
OM721316   ............................................................................G...  2800
```

```
                CGCCTCATTTTGTGGGTCACCATATTCTTGGGAACAAGAGCTACAGCATGGGGCAGAATCTTTCCACCAGCAATCCTCTG
NC 003977       .......................................T........................................ 2880
OM721310        ................................................................................ 2880
OM721311        ................................................................................ 2880
OM721312        ...........A.................................................................... 2880
OM721313        ................................................................................ 2880
OM721314        ...........A.................................................................... 2880
OM721315        ................................................................................ 2880
OM721316        ................................................................................ 2880

                GGATTCTTTCCCGACCACCAGTTGGATCCAGCCTTCAGAGCAAACACCGCAAATCCAGATTGGGACTTCAATCCCAACAA
NC 003977       ................................................................................ 2960
OM721310        ................................................................................ 2960
OM721311        ................................................................................ 2960
OM721312        ................................................................................ 2960
OM721313        ................................................................................ 2960
OM721314        ................................................................................ 2960
OM721315        ...............................................T................................ 2960
OM721316        ................................................................................ 2960

                GGACACCTGGCCAGACGCCAACAAGGTAGGAGCTGGAGCATTCGGGCTGGGATTCACCCCACCGCACGGAGGCCTTTTGG
NC 003977       ...................................................T............................ 3040
OM721310        ................................................................................ 3040
OM721311        ....C........................................................................... 3040
OM721312        ................................................................................ 3040
OM721313        ................................................................................ 3040
OM721314        ................................................................................ 3040
OM721315        ................................................................................ 3040
OM721316        ...............T.....................................................C.......... 3040

                GGTGGAGCCCTCAGGCTCAGGGCATACTACAAACCTTGCCAGCAAATCCGCCTCCTGCCTCTACCAATCGCCAGTCAGGA
NC 003977       ..................................T..........................C.................. 3120
OM721310        .........................................A...................................... 3120
OM721311        .........................................A...................................... 3120
OM721312        ...........................................................G.................... 3120
OM721313        ................................................................................ 3120
OM721314        ...........................................................G.................... 3120
OM721315        .........................................A...................................... 3120
OM721316        ................................................................................ 3120

                AGGCAGCCTACCCCTCTGTCTCCACCTTTGAGAAACACTCATCCTCAGGCCATGCAGTGGAA
NC 003977       ..............G............................................... 3182
OM721310        .............................................................. 3182
OM721311        .............................................................. 3182
OM721312        .............................................................. 3182
OM721313        .............................................................. 3182
OM721314        .............................................................. 3182
OM721315        .............................................................. 3182
OM721316        .................................G............................ 3182
```

Sequence Logo: 50% GC base composition

Compare to: the consensus

Bases that match the reference are marked with dots.

Created: Dec 2, 2023

Last Modified: Dec 2, 2023

S2. Alignment of the obtained nucleotide sequences (OM721310-OM721316) to the reference sequence (NC_003977) in the NCBI GenBank Database using the Clustal W method integrated into the Snapgene software.
