# Supplementary material for: Full-Length Genome Sequencing and Analysis of Hepatitis B Viruses Isolated from Iraqi Patients
Source: Int J Microbiol. 2024 Apr 29;2024:6826495. doi: 10.1155/2024/6826495 (PMC11074772; doi:10.1155/2024/6826495)
Supplement: Supplementary Materials — The metadata of HBV samples is shown in S1, Supplementary Materials, as a PDF file. Alignment of the obtained nucleotide sequences (OM721310–OM721316) to the reference sequence (NC_003977) in the NCBI GenBank database using the Clustal W method integrated into the SnapGene software is shown in S2, Supplementary Materials, as a PDF file. Determination of genotypes of HBV isolates (OM721310–OM721316) using the annotation algorithm in the HBVdb is shown in S3, Supplementary Materials, as a PDF file. Determination of genotypes, subgenotypes of HBV isolates (OM721310–OM721316), and their sensitivity to antiviral drugs using Geno2pheno are shown in S4, Supplementary Materials, as PDF files. Serotype determination of HBV isolates (OM721310–OM721316) using the HBV Serotyper tool is shown in S5, Supplementary Materials, as a PDF file. [file 6826495.f1.zip › S3. Determination of genotypes of HBV isolates (OM721310-OM721316) using the annotation algorithm in the HBVdb (1).pdf]

S3. Determination of genotypes of HBV isolates (OM721310-OM721316) using the annotation algorithm in the HBVdb

HBVdb

* Home <https://hbvdb.lyon.inserm.fr/HBVdb/HBVdbIndex>
* HBV
  o Genome <https://hbvdb.lyon.inserm.fr/HBVdb/HBVdbGenome>
  o Nomenclature <https://hbvdb.lyon.inserm.fr/HBVdb/HBVdbNomenclature>
  o Proteins
    + Core
      <https://hbvdb.lyon.inserm.fr/HBVdb/HBVdbProteins?protein=Core>
    + HBx
      <https://hbvdb.lyon.inserm.fr/HBVdb/HBVdbProteins?protein=HBx>
    + Surface
      <https://hbvdb.lyon.inserm.fr/HBVdb/HBVdbProteins?protein=Surface>
    + Polymerase
      <https://hbvdb.lyon.inserm.fr/HBVdb/HBVdbProteins?protein=Polymerase>
* Query
  o Dataset
    + Nucleotide
      <https://hbvdb.lyon.inserm.fr/HBVdb/HBVdbDataset?seqtype=0>
    + Protein
      <https://hbvdb.lyon.inserm.fr/HBVdb/HBVdbDataset?seqtype=2>
* Analysis
  o Generic N
    + Blast N

<https://npsa.lyon.inserm.fr/cgi-bin/npsa_automat.pl?page=/NPSA/npsa_blastan.html>
    + ClustalW N

<https://npsa.lyon.inserm.fr/cgi-bin/npsa_automat.pl?page=/NPSA/npsa_clustalwan.html
>
    + FASTA N

<https://npsa.lyon.inserm.fr/cgi-bin/npsa_automat.pl?page=/NPSA/npsa_fastaan.html>
  o Generic P
    + Blast P

<https://npsa.lyon.inserm.fr/cgi-bin/npsa_automat.pl?page=/NPSA/npsa_blast.html>
    + ClustalW P

<https://npsa.lyon.inserm.fr/cgi-bin/npsa_automat.pl?page=/NPSA/npsa_clustalw.html>
    + FASTA P

<https://npsa.lyon.inserm.fr/cgi-bin/npsa_automat.pl?page=/NPSA/npsa_fasta.html>
  o Specialized
    + Annotate <https://hbvdb.lyon.inserm.fr/HBVdb/HBVdbAnnotate>
    + Genotype <https://hbvdb.lyon.inserm.fr/HBVdb/HBVdbGenotype>
    + Resistance <https://hbvdb.lyon.inserm.fr/HBVdb/HBVdbResistance>
* HBVdb
  o About <https://hbvdb.lyon.inserm.fr/HBVdb/HBVdbAbout>
  o Contact <https://hbvdb.lyon.inserm.fr/HBVdb/HBVdbContact>

```
      o Help
          + Annotate
            <https://hbvdb.lyon.inserm.fr/HBVdb/HBVdbHelp?userhelp=Annotate>
          + Genotype
            <https://hbvdb.lyon.inserm.fr/HBVdb/HBVdbHelp?userhelp=Genotype>
          + Home <https://hbvdb.lyon.inserm.fr/HBVdb/HBVdbHelp>
          + Resistance
            <https://hbvdb.lyon.inserm.fr/HBVdb/HBVdbHelp?userhelp=Resistance>
      o News <https://hbvdb.lyon.inserm.fr/HBVdb/HBVdbNews>
      o Statistics <https://hbvdb.lyon.inserm.fr/HBVdb/HBVdbStats>
  * Links
      o PRABI
          + databases
              # BCL2DB <https://bcl2db.lyon.inserm.fr/>
              # BYKdb <https://bykdb.lyon.inserm.fr/>
              # euHCVdb <https://euhcvdb.lyon.inserm.fr/>
          + centers
              # all <http://www.prabi.fr/>
              # Gerland <https://prabi.lyon.inserm.fr/>
          + tools
              # GENO3D <https://geno3d.lyon.inserm.fr/>
              # NPS@ <https://npsa.lyon.inserm.fr/>
              # QueBio <https://quebio.lyon.inserm.fr/>
      o external
          + databases
              # ENA <http://www.ebi.ac.uk/ena/>
              # PDB <http://www.wwpdb.org/>
              # UniProtKB <http://www.uniprot.org/>
          + centers
              # EBI <http://www.ebi.ac.uk/>
              # NCBI <http://www.ncbi.nlm.nih.gov/>
              # RCSB <http://www.pdb.org/>
              # SIB <http://www.isb-sib.ch/>
```

Annotate results for OM721310: text entry in EMBL format

```
ID   OM721310; SV 1; circular; genomic DNA; STD; VRL; 3182 BP.
XX
AC   OM721310;
XX
XX
DT   11-FEB-2022 (Rel. 0, Created)
DT   11-FEB-2022 (Rel. 0, Last updated, Version 0)
XX
DE   Hepatitis B Virus genotype D. Complete genome.
XX
KW   HBe; HBc; HBx; LHBs; MHBs; SHBs; Pol; HBSP; complete genome; Sensitive to
drugs.
```

```
XX
OS   Hepatitis B Virus genotype D
OC   Viruses; Retro-transcribing viruses; Hepadnaviridae; Orthohepadnavirus.
XX
RN   1
RP   1-3182
RA   Unknown M;
RL   Submitted (11-FEB-2022) to FR-IBCP-PRABI-ISA.
XX
CC   The data provided in this entry have been computed thanks to
CC   the Hepatitis B Virus Database (HBVdb) annotation alogrithms.
CC   HBVdb is available at http://hbvdb.ibcp.fr.
XX
FH   Key             Location/Qualifiers
FH
FT   source          1..3182
FT                   /mol_type="genomic DNA"
FT                   /organism="Hepatitis B Virus"
FT                   /db_xref="taxon:10407"
FT                   /db_xref="HBVdb:AF121240"
FT                   /PRABI_genotype="n.a.:D:n.a."
FT   CDS             1814..2452
FT                   /PRABI_name="PreC"
FT                   /locus_tag="HBVORF02"
FT                   /codon_start="1"
FT                   /translation="MQLFHLCLIISCSCPTVQASKLCLGWLWGMDIDPYKEFGATVELL
FT                   SFLPSDFFPSVRDLLDTASALYREALESPEHCSPHHTALRQAILCWGELMTLATWVGGN
FT                   LEDPISRDLVVSYVNTNMGLKFRQLLWFHISCLTFGRETVIEYLVSFGVWIRTPPAYRP
FT                   PNAPILSTLPETTVVRRRGRSPRRRTPSPRRRRSQSPRRRRSQSRESQC"
FT   mat_peptide     1871..2275
FT                   /function="HBe/External core antigen coding sequence"
FT                   /locus_tag="HBVORF02"
FT                   /product="External core antigen"
FT                   /PRABI_name="HBe"
FT                   /PRABI_prodft=(pos:20..154, CHAIN, "HBe antigen")
FT   CDS             1901..2452
FT                   /PRABI_name="C"
FT                   /locus_tag="HBVORF12"
FT                   /codon_start="1"
FT                   /translation="MDIDPYKEFGATVELLSFLPSDFFPSVRDLLDTASALYREALESP
FT                   EHCSPHHTALRQAILCWGELMTLATWVGGNLEDPISRDLVVSYVNTNMGLKFRQLLWFH
FT                   ISCLTFGRETVIEYLVSFGVWIRTPPAYRPPNAPILSTLPETTVVRRRGRSPRRRTPSP
FT                   RRRRSQSPRRRRSQSRESQC"
FT   mat_peptide     1901..2449
FT                   /function="Core protein coding sequence"
FT                   /locus_tag="HBVORF12"
FT                   /product="Core protein"
FT                   /PRABI_name="HBc"
FT                   /PRABI_prodft=(pos:1..183, CHAIN, "Core protein")
FT   CDS             1374..1838
```

```
FT                   /PRABI_name="X"
FT                   /locus_tag="HBVORF03"
FT                   /codon_start="1"
FT                   /translation="MAARLCCQLDPARDVLCLRPVGAESRGRPFSGPLGTLSSPSPSAV
FT                   STDHGAHLSLRGLPVCAFSSAGPCALRFTSARRMETTVNAHQFLPKVLHKRTLGLSVMS
FT                   TTDLEAYFKDCLFKDWEELGEEIRLKVFVLGGCRHKLVCAPAPCNFFTSA"
FT   mat_peptide     1374..1835
FT                   /function="X protein coding sequence"
FT                   /locus_tag="HBVORF03"
FT                   /product="X protein"
FT                   /PRABI_name="HBx"
FT                   /PRABI_prodft=(pos:1..154, CHAIN, "X protein")
FT   CDS             join(2848..3182,1..835)
FT                   /PRABI_name="PreS1"
FT                   /locus_tag="HBVORF01"
FT                   /codon_start="1"
FT                   /translation="MGQNLSTSNPLGFFPDHQLDPAFRANTANPDWDFNPNKDTWPDAN
FT                   KVGAGAFGLGFTPPHGGLLGWSPQAQGILQTLPTNPPPASTNRQSGRQPTPLSPPLRNT
FT                   HPQAMQWNSTTFHQTLQDPRVRGLYFPAGGSSSGTVNPVPTTVSHISSIFSRIGDPALN
FT                   MESITSGFLGPLLVLQAGFFLLTRILTIPQSLDSWWTSLNFLGGTTVCLGQNSQSPTSN
FT                   HSPTSCPPTCPGYRWMCLRRFIIFLFILLLCLIFLLVLLDYQGMLPVCPLIPGSSTTST
FT                   GPCRTCTTPAQGTSMYPSCCCTKPSDGNCTCIPIPSSWAFGKFLWEWASARFSWLSLLV
FT                   PFVQWFVGLSPTVWLSVIWMMWYWGPSLYSILSPFLPLLPIFFCLWVYI"
FT   mat_peptide     join(2848..3182,1..832)
FT                   /function="PreS1/Large Surface protein coding sequence"
FT                   /locus_tag="HBVORF01"
FT                   /product="PreS1 Surface protein"
FT                   /PRABI_name="LHBs"
FT                   /PRABI_prodft=(pos:1..389, CHAIN, "Large Surface protein")
FT   CDS             join(3172..3182,1..835)
FT                   /PRABI_name="PreS2"
FT                   /locus_tag="HBVORF11"
FT                   /codon_start="1"
FT                   /translation="MQWNSTTFHQTLQDPRVRGLYFPAGGSSSGTVNPVPTTVSHISSI
FT                   FSRIGDPALNMESITSGFLGPLLVLQAGFFLLTRILTIPQSLDSWWTSLNFLGGTTVCL
FT                   GQNSQSPTSNHSPTSCPPTCPGYRWMCLRRFIIFLFILLLCLIFLLVLLDYQGMLPVCP
FT                   LIPGSSTTSTGPCRTCTTPAQGTSMYPSCCCTKPSDGNCTCIPIPSSWAFGKFLWEWAS
FT                   ARFSWLSLLVPFVQWFVGLSPTVWLSVIWMMWYWGPSLYSILSPFLPLLPIFFCLWVYI
FT                   "
FT   mat_peptide     join(3172..3182,1..832)
FT                   /function="PreS2/Middle Surface protein coding sequence"
FT                   /locus_tag="HBVORF11"
FT                   /product="PreS2 Surface protein"
FT                   /PRABI_name="MHBs"
FT                   /PRABI_prodft=(pos:1..281, CHAIN, "Middle Surface protein")
FT   CDS             155..835
FT                   /PRABI_name="S"
FT                   /locus_tag="HBVORF21"
FT                   /codon_start="1"
FT                   /translation="MESITSGFLGPLLVLQAGFFLLTRILTIPQSLDSWWTSLNFLGGT
```

```
FT                   TVCLGQNSQSPTSNHSPTSCPPTCPGYRWMCLRRFIIFLFILLLCLIFLLVLLDYQGML
FT                   PVCPLIPGSSTTSTGPCRTCTTPAQGTSMYPSCCCTKPSDGNCTCIPIPSSWAFGKFLW
FT                   EWASARFSWLSLLVPFVQWFVGLSPTVWLSVIWMMWYWGPSLYSILSPFLPLLPIFFCL
FT                   WVYI"
FT   mat_peptide     155..832
FT                   /function="S protein coding sequence"
FT                   /locus_tag="HBVORF21"
FT                   /product="Surface protein S"
FT                   /PRABI_name="SHBs"
FT                   /PRABI_prodft=(pos:1..226, CHAIN, "Small Surface protein")
FT   CDS             join(2307..3182,1..1623)
FT                   /PRABI_name="P"
FT                   /locus_tag="HBVORF13"
FT                   /codon_start="1"
FT                   /translation="MPLSYQHFRRLLLLDDEAGPLEEELPRLADEGLNRRVAEDLNLGN
FT                   LNVSIPWTHKVGNFTGLYSSTVPVFNPHWKTPSFPNIHLHQDIIKKCEQFVGPLTVNEK
FT                   RRLQLIMPARFYPNITKYLPLDKGIKPYYPEHLVNHYFQTRHYLHTLWKAGILYKRETT
FT                   HSASFCGSPYSWEQELQHGAESFHQQSSGILSRPPVGSSLQSKHRKSRLGLQSQQGHLA
FT                   RRQQGRSWSIRAGIHPTARRPFGVEPSGSGHTTNLANKSASCLYQSPVRKAAYPSVSTF
FT                   EKHSSSGHAVELHNLPPNSARSQSERPVFPCWWLQFRNSKPCSDYCLSHIVNLLEDWGP
FT                   CAEHGEHHIRIPRTPARVTGGVFLVDKNPHNTAESRLVVDFSQFSRGNYRVSWPKFAVP
FT                   NLQSLTNLLSSNLSWLSLDVSAAFYHLPLHPAAMPHLLVGSSGLSRYVARLSSNSRIFN
FT                   HQHGTMQNLHDSCSRNLYVSLLLLYQTFGRKLHLYSHPIILGFRKIPMGVGLSPFLLAQ
FT                   FTSAICSVVRRAFPHCLAFSYMDDVVLGAKSVQHLESLFTAVTNFLLSLGIHLNPNKTK
FT                   RWGYSLHFMGYVIGCYGSLPQDHIIQKIKECFRKLPVNRPIDWKVCQRIVGLLGFAAPF
FT                   TQCGYPALMPLYACIQSKQAFTFSPTYKAFLCKQYLNLYPVARQRPGLCQVFADATPTG
FT                   WGLVMGHQRMRGTFLAPLPIHTAELLAACFARSRSGANILGTDNSVVLSRKYTSFPWLL
FT                   GCAANWILRGTSFVYVPSALNPADDPSRGRLGLSRPLLRLPFRPTTGRTSLYADSPSVP
FT                   SHLPDRVHFASPLHVAWRPP"
FT   mat_peptide     join(2307..3182,1..1620)
FT                   /function="DNA-polymerase/Reverse Transcriptase coding
FT                   sequence"
FT                   /locus_tag="HBVORF13"
FT                   /product="Polymerase/Reverse Transcriptase"
FT                   /PRABI_name="Pol"
FT                   /PRABI_prodft=(pos:1..832, CHAIN, "Polymerase/Reverse
FT                   transcriptase")
FT                   /PRABI_prodft=(pos:1..180, DOMAIN, "Terminal Protein (TP)/
FT                   Primase domain")
FT                   /PRABI_prodft=(pos:181..335, DOMAIN, "Spacer")
FT                   /PRABI_prodft=(pos:336..679, DOMAIN, "Reverse Transcriptase
FT                   (RT) domain")
FT                   /PRABI_prodft=(pos:418..418, ACT_SITE, "RT catalytic Asp")
FT                   /PRABI_prodft=(pos:540..540, ACT_SITE, "RT catalytic Asp")
FT                   /PRABI_prodft=(pos:541..541, ACT_SITE, "RT catalytic Asp")
FT                   /PRABI_prodft=(pos:680..832, DOMAIN, "Ribonuclease H
FT                   (RNaseH) domain")
FT                   /PRABI_prodft=(pos:689..689, ACT_SITE, "RNaseH catalytic
FT                   Asp")
FT                   /PRABI_prodft=(pos:718..718, ACT_SITE, "RNaseH catalytic
```

```
FT                   Glu")
FT                   /PRABI_prodft=(pos:737..737, ACT_SITE, "RNaseH catalytic
FT                   Asp")
FT   CDS             join(2307..2447,489..683)
FT                   /PRABI_name="SP"
FT                   /locus_tag="HBVORF04"
FT                   /codon_start="1"
FT                   /translation="MPLSYQHFRRLLLLDDEAGPLEEELPRLADEGLNRRVAEDLNLGN
FT                   LNDLQPPARDHAEPARLLLKEPLCIPPVAVPNLRTEIAPVFPSHHPGLSENSYGSGPQP
FT                   VSPGSVY"
FT   mat_peptide     join(2307..2447,489..680)
FT                   /function="HBSP coding sequence"
FT                   /locus_tag="HBVORF04"
FT                   /product="Hepatitis B Spliced Protein"
FT                   /PRABI_name="HBSP"
FT                   /PRABI_prodft=(pos:1..111, CHAIN, "HBV Spliced Protein")
XX
SQ   Sequence 3182 BP; 730 A; 860 C; 688 G; 904 T; 0 other;
     ctccacaacc ttccaccaaa ctctgcaaga tcccagagtg agaggcctgt atttccctgc        60
     tggtggctcc agttcaggaa cagtaaaccc tgttccgact actgtctctc acatatcgtc       120
     aatcttctcg aggattgggg accctgcgct gaacatggag agcatcacat caggattcct       180
     aggacccctg ctcgtgttac aggcggggtt tttcttgttg acaagaatcc tcacaatacc       240
     gcagagtcta gactcgtggt ggacttctct caattttcta gggggaacta ccgtgtgtct       300
     tggccaaaat tcgcagtccc caacctccaa tcactcacca acctcctgtc ctccaacttg       360
     tcctggttat cgctggatgt gtctgcggcg ttttatcatc ttcctcttca tcctgctgct       420
     atgcctcatc ttcttgttgg ttcttctgga ctatcaaggt atgttgcccg tttgtcctct       480
     aattccagga tcttcaacca ccagcacggg accatgcaga acctgcacga ctcctgctca       540
     aggaacctct atgtatccct cctgttgctg taccaaacct tcggacggaa attgcacctg       600
     tattcccatc ccatcatcct gggctttcgg aaaattccta tgggagtggg cctcagcccg       660
     tttctcctgg ctcagtttac tagtgccatt tgttcagtgg ttcgtagggc tttcccccac       720
     tgtttggctt tcagttatat ggatgatgtg gtattggggg ccaagtctgt acagcatctt       780
     gagtcccttt ttaccgctgt taccaatttt cttttgtctt tgggtataca tttaaaccct       840
     aacaaaacaa aaagatgggg gtactcctta catttcatgg gctatgtcat tggatgttat       900
     ggttcattgc cacaagatca catcatacag aaaatcaaag aatgttttag aaaacttcct       960
     gttaacaggc ctattgattg gaaagtctgt caacgtattg tgggtctttt gggttttgct      1020
     gccccttta cacaatgtgg ttatcctgct ttaatgcctt tgtatgcatg tattcaatct      1080
     aagcaggctt tcactttctc gccaacttac aaggcctttc tgtgtaaaca atacctgaac      1140
     ctttaccccg ttgcccggca acggccaggt ctgtgccaag tgtttgctga cgcaaccccc      1200
     actggctggg gcttggtcat gggccatcag cgcatgcgtg gaacctttct ggctcctctg      1260
     ccgatccata ctgcggaact cctagccgct tgctttgctc gcagcaggtc tggagcaaac      1320
     attcttggga cggataactc tgttgttctc tcccgcaaat atacatcatt tccatggctg      1380
     ctaggctgtg ctgccaactg gatcctgcgc gggacgtcct ttgtttacgt cccgtcggcg      1440
     ctgaatcccg cggacgaccc ttctcggggc cgcttgggac tctctcgtcc ccttctccgt      1500
     ctgccgtttc gaccgaccac ggggcgcacc tctctttacg cggactcccc gtctgtgcct      1560
     tctcatctgc cggaccgtgt gcacttcgct tcacctctgc acgtcgcatg gagaccaccg      1620
     tgaacgccca ccaattcttg cccaaggtct tacataagag gactcttgga ctctctgtaa      1680
     tgtcaacgac cgaccttgag gcatacttca aagactgttt gtttaaggac tgggaggagt      1740
     tgggggagga gattagatta aaggtctttg tattaggagg ctgtaggcat aaattggtct      1800
     gcgcaccagc accatgcaac tttttcacct ctgcctaatc atctcttgtt catgtcctac      1860
     tgttcaagcc tccaagctgt gccttgggtg gctttggggc atggacattg atccttataa      1920
```

```
     agaatttgga gctactgtgg agttactctc gtttttgcct tctgacttct ttccttcagt      1980
     acgagatctt ctagataccg cctcagctct gtatcgggaa gccttagagt ctcctgagca      2040
     ttgttcacct caccatactg cactcaggca agcaattctt tgctgggggg aactaatgac      2100
     tctagccacc tgggtgggtg gtaatttgga agatccaata tccagagacc tagtagtcag      2160
     ttatgttaac actaatatgg gcctaaagtt caggcaacta ttgtggtttc acatttcttg      2220
     tctcactttt ggaagagaaa cggtcataga gtatttggtg tctttcggag tgtggattcg      2280
     cactcctcca gcttatagac caccaaatgc ccctatctta tcaacacttc cggagactac      2340
     tgttgttaga cgacgaggca ggtcccctag aagaagaact ccctcgcctc gcagacgaag      2400
     gtctcaatcg ccgcgtcgca gaagatctca atctcgggaa tctcaatgtt agtattcctt      2460
     ggactcataa ggtgggaaac tttacggggc tttactcttc tactgttcct gtctttaacc      2520
     ctcattggaa aacaccctct tttcctaata tacatttaca ccaagacatt atcaaaaaat      2580
     gtgaacaatt tgtaggccca ctcacagtca atgagaaaag aagactgcaa ttaattatgc      2640
     ctgctaggtt ttatccaaat attaccaaat atttgccatt ggataagggc attaaaccct      2700
     attatccaga acatctagtt aatcattact tccaaaccag acattattta cacactctat      2760
     ggaaggcggg tatattatat aagagagaaa caacacatag cgcctcattt tgtgggtcac      2820
     catattcttg ggaacaagag ctacagcatg gggcagaatc tttccaccag caatcctctg      2880
     ggattctttc ccgaccacca gttggatcca gccttcagag caaacaccgc aaatccagat      2940
     tgggacttca atcccaacaa ggacacctgg ccagacgcca acaaggtagg agctggagca      3000
     ttcgggctgg gattcacccc accgcacgga ggccttttgg ggtggagccc tcaggctcag      3060
     ggcatactac aaaccttgcc aacaaatccg cctcctgcct ctaccaatcg ccagtcagga      3120
     aggcagccta cccctctgtc tccacctttg agaaacactc atcctcaggc catgcagtgg      3180
     aa                                                                    3182
//
```

© 1998-2022 Centre de Recherche en Cancerologie de Lyon logo <http://www.crcl.fr/> Pole Rhone-Alpes de BioInformatique logo <http://prabi.ibcp.fr/> Centre National de la Recherche Scientifique logo <http://www.cnrs.fr/> Institut national de la sante et de la recherche medicale logo <http://www.inserm.fr/> Universite Claude Bernard Lyon 1 logo <http://www.univ-lyon1.fr/> Legal notice <https://hbvdb.lyon.inserm.fr/HBVdb/HBVdbAbout#legalnotice>

HBVdb

* Home <https://hbvdb.lyon.inserm.fr/HBVdb/HBVdbIndex>
* HBV
    o Genome <https://hbvdb.lyon.inserm.fr/HBVdb/HBVdbGenome>
    o Nomenclature <https://hbvdb.lyon.inserm.fr/HBVdb/HBVdbNomenclature>
    o Proteins
        + Core
          <https://hbvdb.lyon.inserm.fr/HBVdb/HBVdbProteins?protein=Core>
        + HBx
          <https://hbvdb.lyon.inserm.fr/HBVdb/HBVdbProteins?protein=HBx>
        + Surface
          <https://hbvdb.lyon.inserm.fr/HBVdb/HBVdbProteins?protein=Surface>
        + Polymerase
          <https://hbvdb.lyon.inserm.fr/HBVdb/HBVdbProteins?protein=Polymerase>
* Query
    o Dataset
        + Nucleotide
          <https://hbvdb.lyon.inserm.fr/HBVdb/HBVdbDataset?seqtype=0>
        + Protein
          <https://hbvdb.lyon.inserm.fr/HBVdb/HBVdbDataset?seqtype=2>
* Analysis
    o Generic N
        + Blast N

<https://npsa.lyon.inserm.fr/cgi-bin/npsa_automat.pl?page=/NPSA/npsa_blastan.html>
        + ClustalW N

<https://npsa.lyon.inserm.fr/cgi-bin/npsa_automat.pl?page=/NPSA/npsa_clustalwan.html
>
        + FASTA N

<https://npsa.lyon.inserm.fr/cgi-bin/npsa_automat.pl?page=/NPSA/npsa_fastaan.html>
    o Generic P
        + Blast P

<https://npsa.lyon.inserm.fr/cgi-bin/npsa_automat.pl?page=/NPSA/npsa_blast.html>
        + ClustalW P

<https://npsa.lyon.inserm.fr/cgi-bin/npsa_automat.pl?page=/NPSA/npsa_clustalw.html>
        + FASTA P

<https://npsa.lyon.inserm.fr/cgi-bin/npsa_automat.pl?page=/NPSA/npsa_fasta.html>
    o Specialized
        + Annotate <https://hbvdb.lyon.inserm.fr/HBVdb/HBVdbAnnotate>
        + Genotype <https://hbvdb.lyon.inserm.fr/HBVdb/HBVdbGenotype>
        + Resistance <https://hbvdb.lyon.inserm.fr/HBVdb/HBVdbResistance>
* HBVdb
    o About <https://hbvdb.lyon.inserm.fr/HBVdb/HBVdbAbout>
    o Contact <https://hbvdb.lyon.inserm.fr/HBVdb/HBVdbContact>

Annotate results for OM721311: text entry in EMBL format

```
ID   OM721311; SV 1; circular; genomic DNA; STD; VRL; 3182 BP.
XX
AC   OM721311;
XX
XX
DT   11-FEB-2022 (Rel. 0, Created)
DT   11-FEB-2022 (Rel. 0, Last updated, Version 0)
XX
DE   Hepatitis B Virus genotype D. Complete genome.
XX
KW   HBe; HBc; HBx; LHBs; MHBs; SHBs; Pol; HBSP; complete genome; Sensitive to
drugs.
```

```
XX
OS   Hepatitis B Virus genotype D
OC   Viruses; Retro-transcribing viruses; Hepadnaviridae; Orthohepadnavirus.
XX
RN   1
RP   1-3182
RA   Unknown M;
RL   Submitted (11-FEB-2022) to FR-IBCP-PRABI-ISA.
XX
CC   The data provided in this entry have been computed thanks to
CC   the Hepatitis B Virus Database (HBVdb) annotation alogrithms.
CC   HBVdb is available at http://hbvdb.ibcp.fr.
XX
FH   Key             Location/Qualifiers
FH
FT   source          1..3182
FT                   /mol_type="genomic DNA"
FT                   /organism="Hepatitis B Virus"
FT                   /db_xref="taxon:10407"
FT                   /db_xref="HBVdb:AF121240"
FT                   /PRABI_genotype="n.a.:D:n.a."
FT   CDS             1814..2452
FT                   /PRABI_name="PreC"
FT                   /locus_tag="HBVORF02"
FT                   /codon_start="1"
FT                   /translation="MQLFHLCLIISCSCPTVQASKLCLGWLWGMDIDPYKEFGATVELL
FT                   SFLPSDFFPSVRHLLDTASALYREALESPEHCSPHHTALRQAILCWGELMTLATWVGGN
FT                   LEDPISRDLVVSYVNTNMGLKFRQLLWFHISCLTFGRETVIEYLVSFGVWIRTPPAYRP
FT                   PNAPILSTLPETTVVRRRGRSPRRRTPSPRRRRSQSPRRRRSQSRESQC"
FT   mat_peptide     1871..2275
FT                   /function="HBe/External core antigen coding sequence"
FT                   /locus_tag="HBVORF02"
FT                   /product="External core antigen"
FT                   /PRABI_name="HBe"
FT                   /PRABI_prodft=(pos:20..154, CHAIN, "HBe antigen")
FT   CDS             1901..2452
FT                   /PRABI_name="C"
FT                   /locus_tag="HBVORF12"
FT                   /codon_start="1"
FT                   /translation="MDIDPYKEFGATVELLSFLPSDFFPSVRHLLDTASALYREALESP
FT                   EHCSPHHTALRQAILCWGELMTLATWVGGNLEDPISRDLVVSYVNTNMGLKFRQLLWFH
FT                   ISCLTFGRETVIEYLVSFGVWIRTPPAYRPPNAPILSTLPETTVVRRRGRSPRRRTPSP
FT                   RRRRSQSPRRRRSQSRESQC"
FT   mat_peptide     1901..2449
FT                   /function="Core protein coding sequence"
FT                   /locus_tag="HBVORF12"
FT                   /product="Core protein"
FT                   /PRABI_name="HBc"
FT                   /PRABI_prodft=(pos:1..183, CHAIN, "Core protein")
FT   CDS             1374..1838
```

```
FT                   /PRABI_name="X"
FT                   /locus_tag="HBVORF03"
FT                   /codon_start="1"
FT                   /translation="MAARLCCQLDPARDVLCLRPVGAESRGRPFSGPLGTLSSPSPSAV
FT                   STDHGAHLSLRGLPVCAFSSAGPCALRFTSARRMETTVNAHQFLPKVLHKRTLGLSVMS
FT                   TTDLEAYFKDCLFKDWEELGEEIRLKVFVLGGCRHKLVCAPAPCNFFTSA"
FT   mat_peptide     1374..1835
FT                   /function="X protein coding sequence"
FT                   /locus_tag="HBVORF03"
FT                   /product="X protein"
FT                   /PRABI_name="HBx"
FT                   /PRABI_prodft=(pos:1..154, CHAIN, "X protein")
FT   CDS             join(2848..3182,1..835)
FT                   /PRABI_name="PreS1"
FT                   /locus_tag="HBVORF01"
FT                   /codon_start="1"
FT                   /translation="MGQNLSTSNPLGFFPDHQLDPAFRANTANPDWDFNPNKDPWPDAN
FT                   KVGAGAFGLGFTPPHGGLLGWSPQAQGILQTLPTNPPPASTNRQSGRQPTPLSPPLRNT
FT                   HPQAMQWNSTTFHQTLQDPRVRGLYFPAGGSSSGTVNPVPTTVSHISSIFSRIGDPALN
FT                   MESITSGFLGPLLVLQAGFFLLTRILTIPQSLDSWWTSLNFRGGTTVCLGQNSQSPTSN
FT                   HSPTSCPPTCPGYRWMCLRRFIIFLFILLLCLIFLLVLLDYQGMLPVCPLIPGSSTTST
FT                   GPCRTCTTPAQGTSMYPSCCCTKPSDGNCTCIPIPSSWAFGKFLWEWASARFSWLSSLV
FT                   PFVQWFVGLSPTVWLSVIWMMWYWGPSLYSILSPFLPLLPIFFCLWVYI"
FT   mat_peptide     join(2848..3182,1..832)
FT                   /function="PreS1/Large Surface protein coding sequence"
FT                   /locus_tag="HBVORF01"
FT                   /product="PreS1 Surface protein"
FT                   /PRABI_name="LHBs"
FT                   /PRABI_prodft=(pos:1..389, CHAIN, "Large Surface protein")
FT   CDS             join(3172..3182,1..835)
FT                   /PRABI_name="PreS2"
FT                   /locus_tag="HBVORF11"
FT                   /codon_start="1"
FT                   /translation="MQWNSTTFHQTLQDPRVRGLYFPAGGSSSGTVNPVPTTVSHISSI
FT                   FSRIGDPALNMESITSGFLGPLLVLQAGFFLLTRILTIPQSLDSWWTSLNFRGGTTVCL
FT                   GQNSQSPTSNHSPTSCPPTCPGYRWMCLRRFIIFLFILLLCLIFLLVLLDYQGMLPVCP
FT                   LIPGSSTTSTGPCRTCTTPAQGTSMYPSCCCTKPSDGNCTCIPIPSSWAFGKFLWEWAS
FT                   ARFSWLSSLVPFVQWFVGLSPTVWLSVIWMMWYWGPSLYSILSPFLPLLPIFFCLWVYI
FT                   "
FT   mat_peptide     join(3172..3182,1..832)
FT                   /function="PreS2/Middle Surface protein coding sequence"
FT                   /locus_tag="HBVORF11"
FT                   /product="PreS2 Surface protein"
FT                   /PRABI_name="MHBs"
FT                   /PRABI_prodft=(pos:1..281, CHAIN, "Middle Surface protein")
FT   CDS             155..835
FT                   /PRABI_name="S"
FT                   /locus_tag="HBVORF21"
FT                   /codon_start="1"
FT                   /translation="MESITSGFLGPLLVLQAGFFLLTRILTIPQSLDSWWTSLNFRGGT
```

```
FT                   TVCLGQNSQSPTSNHSPTSCPPTCPGYRWMCLRRFIIFLFILLLCLIFLLVLLDYQGML
FT                   PVCPLIPGSSTTSTGPCRTCTTPAQGTSMYPSCCCTKPSDGNCTCIPIPSSWAFGKFLW
FT                   EWASARFSWLSSLVPFVQWFVGLSPTVWLSVIWMMWYWGPSLYSILSPFLPLLPIFFCL
FT                   WVYI"
FT   mat_peptide     155..832
FT                   /function="S protein coding sequence"
FT                   /locus_tag="HBVORF21"
FT                   /product="Surface protein S"
FT                   /PRABI_name="SHBs"
FT                   /PRABI_prodft=(pos:1..226, CHAIN, "Small Surface protein")
FT   CDS             join(2307..3182,1..1623)
FT                   /PRABI_name="P"
FT                   /locus_tag="HBVORF13"
FT                   /codon_start="1"
FT                   /translation="MPLSYQHFRRLLLLDDEAGPLEEELPRLADEGLNRRVAEDLNLGN
FT                   LNVSIPWTHKVGNFTGLYSSTVPVFNPHWKTPSFPNIHLHQDIIKKCEQFVGPLTVNEK
FT                   RRLQLIMPARFYPNITKYLPLDKGIKPYYPEHLVNHYFQTRHYLHTLWKAGILYKRETT
FT                   HSASFCGSPYSWEQELQHGAESFHQQSSGILSRPPVGSSLQSKHRKSRLGLQSQQGPLA
FT                   RRQQGRSWSIRAGIHPTARRPFGVEPSGSGHTTNLANKSASCLYQSPVRKAAYPSVSTF
FT                   EKHSSSGHAVELHNLPPNSARSQSERPVFPCWWLQFRNSKPCSDYCLSHIVNLLEDWGP
FT                   CAEHGEHHIRIPRTPARVTGGVFLVDKNPHNTAESRLVVDFSQFSRGNYRVSWPKFAVP
FT                   NLQSLTNLLSSNLSWLSLDVSAAFYHLPLHPAAMPHLLVGSSGLSRYVARLSSNSRIFN
FT                   HQHGTMQNLHDSCSRNLYVSLLLLYQTFGRKLHLYSHPIILGFRKIPMGVGLSPFLLAQ
FT                   FTSAICSVVRRAFPHCLAFSYMDDVVLGAKSVQHLESLFTAVTNFLLSLGIHLNPNKTK
FT                   RWGYSLHFMGYVIGCYGSLPQDHIIQKIKECFRKLPVNRPIDWKVCQRIVGLLGFAAPF
FT                   TQCGYPALMPLYACIQSKQAFTFSPTYKAFLCKQYLNLYPVARQRPGLCQVFADATPTG
FT                   WGLVMGHQRMRGTFQAPLPIHTAELLAACFARSRSGANILGTDNSVVLSRKYTSFPWLL
FT                   GCAANWILRGTSFVYVPSALNPADDPSRGRLGLSRPLLRLPFRPTTGRTSLYADSPSVP
FT                   SHLPDRVHFASPLHVAWRPP"
FT   mat_peptide     join(2307..3182,1..1620)
FT                   /function="DNA-polymerase/Reverse Transcriptase coding
FT                   sequence"
FT                   /locus_tag="HBVORF13"
FT                   /product="Polymerase/Reverse Transcriptase"
FT                   /PRABI_name="Pol"
FT                   /PRABI_prodft=(pos:1..832, CHAIN, "Polymerase/Reverse
FT                   transcriptase")
FT                   /PRABI_prodft=(pos:1..180, DOMAIN, "Terminal Protein (TP)/
FT                   Primase domain")
FT                   /PRABI_prodft=(pos:181..335, DOMAIN, "Spacer")
FT                   /PRABI_prodft=(pos:336..679, DOMAIN, "Reverse Transcriptase
FT                   (RT) domain")
FT                   /PRABI_prodft=(pos:418..418, ACT_SITE, "RT catalytic Asp")
FT                   /PRABI_prodft=(pos:540..540, ACT_SITE, "RT catalytic Asp")
FT                   /PRABI_prodft=(pos:541..541, ACT_SITE, "RT catalytic Asp")
FT                   /PRABI_prodft=(pos:680..832, DOMAIN, "Ribonuclease H
FT                   (RNaseH) domain")
FT                   /PRABI_prodft=(pos:689..689, ACT_SITE, "RNaseH catalytic
FT                   Asp")
FT                   /PRABI_prodft=(pos:718..718, ACT_SITE, "RNaseH catalytic
```

```
FT                   Glu")
FT                   /PRABI_prodft=(pos:737..737, ACT_SITE, "RNaseH catalytic
FT                   Asp")
FT   CDS             join(2307..2447,489..683)
FT                   /PRABI_name="SP"
FT                   /locus_tag="HBVORF04"
FT                   /codon_start="1"
FT                   /translation="MPLSYQHFRRLLLLDDEAGPLEEELPRLADEGLNRRVAEDLNLGN
FT                   LNDLQPPARDHAEPARLLLKEPLCIPPVAVPNLRTEIAPVFPSHHPGLSENSYGSGPQP
FT                   VSPGSVH"
FT   mat_peptide     join(2307..2447,489..680)
FT                   /function="HBSP coding sequence"
FT                   /locus_tag="HBVORF04"
FT                   /product="Hepatitis B Spliced Protein"
FT                   /PRABI_name="HBSP"
FT                   /PRABI_prodft=(pos:1..111, CHAIN, "HBV Spliced Protein")
XX
SQ   Sequence 3182 BP; 729 A; 863 C; 689 G; 901 T; 0 other;
     ctccacaacc ttccaccaaa ctctgcaaga tcccagagtg agaggcctgt atttccctgc        60
     tggtggctcc agttcaggaa cagtaaaccc tgttccgact actgtctctc acatatcgtc       120
     aatcttctcg aggattgggg accctgcgct gaacatggag agcatcacat caggattcct       180
     aggacccctg ctcgtgttac aggcggggtt tttcttgttg acaagaatcc tcacaatacc       240
     gcagagtcta gactcgtggt ggacttctct caattttcga gggggaacta ccgtgtgtct       300
     tggccaaaat tcgcagtccc caacctccaa tcactcacca acctcctgtc ctccaacttg       360
     tcctggttat cgctggatgt gtctgcggcg ttttatcatc ttcctcttca tcctgctgct       420
     atgcctcatc ttcttgttgg ttcttctgga ctatcaaggt atgttgcccg tttgtcctct       480
     aattccagga tcttcaacca ccagcacggg accatgcaga acctgcacga ctcctgctca       540
     aggaacctct atgtatccct cctgttgctg taccaaacct tcggacggaa attgcacctg       600
     tattcccatc ccatcatcct gggctttcgg aaaattccta tgggagtggg cctcagcccg       660
     tttctcctgg ctcagttcac tagtgccatt tgttcagtgg ttcgtagggc tttcccccac       720
     tgtttggctt tcagttatat ggatgatgtg gtattggggg ccaagtctgt acagcatctt       780
     gagtcccttt ttaccgctgt taccaatttt cttttgtctt tgggtataca tttaaaccct       840
     aacaaaacaa aaagatgggg gtactcctta catttcatgg gctatgtcat tggatgttat       900
     ggttcattgc cacaagatca catcatacag aaaatcaaag aatgttttag aaaacttcct       960
     gttaacaggc ctattgattg gaaagtctgt caacgtattg tgggtctttt gggttttgct      1020
     gcccctttta cacaatgtgg ttatcctgct ttaatgcctt tgtatgcatg tattcaatct      1080
     aagcaggctt tcactttctc gccaacttac aaggcctttc tgtgtaaaca atacctgaac      1140
     ctttaccccg ttgcccggca acggccaggt ctgtgccaag tgtttgctga cgcaaccccc      1200
     actggctggg gcttggtcat gggccatcag cgcatgcgtg gaacctttca ggctcctctg      1260
     ccgatccata ctgcggaact cctagccgct tgctttgctc gcagcaggtc tggagcaaac      1320
     attcttggga cggataactc tgttgttctc tcccgcaaat atacatcgtt tccatggctg      1380
     ctaggctgtg ctgccaactg gatcctgcgc gggacgtcct ttgtttacgt cccgtcggcg      1440
     ctgaatcccg cggacgaccc ttctcggggc cgcttgggac tctctcgtcc ccttctccgt      1500
     ctgccgtttc gaccgaccac ggggcgcacc tctctttacg cggactcccc gtctgtgcct      1560
     tctcatctgc cggaccgtgt gcacttcgct tcacctctgc acgtcgcatg gagaccaccg      1620
     tgaacgccca ccaattcttg cccaaggtct tacataagag gactcttgga ctctctgtaa      1680
     tgtcaacgac cgaccttgag gcatacttca aagactgttt gtttaaggac tgggaggagt      1740
     tgggggagga gattagatta aaggtctttg tattaggagg ctgtaggcat aaattggtct      1800
     gcgcaccagc accatgcaac tttttcacct ctgcctaatc atctcttgtt catgtcctac      1860
     tgttcaagcc tccaagctgt gccttgggtg gctttggggc atggacattg atccttataa      1920
```

```
     agaatttgga gctactgtgg agttactctc gtttttgcct tctgacttct ttccttcagt      1980
     acgacatctt ctagataccg cctcagctct gtatcgggaa gccttagagt ctcctgagca      2040
     ttgttcacct caccatactg cactcaggca agcaattctt tgctgggggg aactaatgac      2100
     tctagccacc tgggtgggtg gtaatttgga agatccaata tccagagacc tagtagtcag      2160
     ttatgttaac actaatatgg gcctaaagtt caggcaacta ttgtggtttc acatttcttg      2220
     tctcactttt ggaagagaaa cggtcataga gtatttggtg tctttcggag tgtggattcg      2280
     cactcctcca gcttatagac caccaaatgc ccctatctta tcaacacttc cggagactac      2340
     tgttgttaga cgacgaggca ggtcccctag aagaagaact ccctcgcctc gcagacgaag      2400
     gtctcaatcg ccgcgtcgca gaagatctca atctcgggaa tctcaatgtt agtattcctt      2460
     ggactcataa ggtgggaaac tttacggggc tttactcttc tactgttcct gtctttaacc      2520
     ctcattggaa aacaccctct tttcctaata tacatttaca ccaagacatt atcaaaaaat      2580
     gtgaacaatt tgtaggccca ctcacagtca atgagaaaag aagactgcaa ttaattatgc      2640
     ctgctaggtt ttatccaaat attaccaaat atttgccatt ggataagggc attaaaccct      2700
     attatccaga acatctagtt aatcattact tccaaaccag acattattta cacactctat      2760
     ggaaggcggg tatattatat aagagagaaa caacacatag cgcctcattt tgtgggtcac      2820
     catattcttg ggaacaagag ctacagcatg gggcagaatc tttccaccag caatcctctg      2880
     ggattctttc ccgaccacca gttggatcca gccttcagag caaacaccgc aaatccagat      2940
     tgggacttca atcccaacaa ggacccctgg ccagacgcca acaaggtagg agctggagca      3000
     ttcgggctgg gattcacccc accgcacgga ggccttttgg ggtggagccc tcaggctcag      3060
     ggcatactac aaaccttgcc aacaaatccg cctcctgcct ctaccaatcg ccagtcagga      3120
     aggcagccta cccctctgtc tccacctttg agaaacactc atcctcaggc catgcagtgg      3180
     aa                                                                     3182
//
```

© 1998-2022 Centre de Recherche en Cancerologie de Lyon logo <http://www.crcl.fr/> Pole Rhone-Alpes de BioInformatique logo <http://prabi.ibcp.fr/> Centre National de la Recherche Scientifique logo <http://www.cnrs.fr/> Institut national de la sante et de la recherche medicale logo <http://www.inserm.fr/> Universite Claude Bernard Lyon 1 logo <http://www.univ-lyon1.fr/> Legal notice <https://hbvdb.lyon.inserm.fr/HBVdb/HBVdbAbout#legalnotice>

HBVdb

  * Home <https://hbvdb.lyon.inserm.fr/HBVdb/HBVdbIndex>
  * HBV
      o Genome <https://hbvdb.lyon.inserm.fr/HBVdb/HBVdbGenome>
      o Nomenclature <https://hbvdb.lyon.inserm.fr/HBVdb/HBVdbNomenclature>
      o Proteins
          + Core
            <https://hbvdb.lyon.inserm.fr/HBVdb/HBVdbProteins?protein=Core>
          + HBx
            <https://hbvdb.lyon.inserm.fr/HBVdb/HBVdbProteins?protein=HBx>
          + Surface
            <https://hbvdb.lyon.inserm.fr/HBVdb/HBVdbProteins?protein=Surface>
          + Polymerase
            <https://hbvdb.lyon.inserm.fr/HBVdb/HBVdbProteins?protein=Polymerase>
  * Query
      o Dataset
          + Nucleotide
            <https://hbvdb.lyon.inserm.fr/HBVdb/HBVdbDataset?seqtype=0>
          + Protein
            <https://hbvdb.lyon.inserm.fr/HBVdb/HBVdbDataset?seqtype=2>
  * Analysis
      o Generic N
          + Blast N

<https://npsa.lyon.inserm.fr/cgi-bin/npsa_automat.pl?page=/NPSA/npsa_blastan.html>
          + ClustalW N

<https://npsa.lyon.inserm.fr/cgi-bin/npsa_automat.pl?page=/NPSA/npsa_clustalwan.html
>
          + FASTA N

<https://npsa.lyon.inserm.fr/cgi-bin/npsa_automat.pl?page=/NPSA/npsa_fastaan.html>
      o Generic P
          + Blast P

<https://npsa.lyon.inserm.fr/cgi-bin/npsa_automat.pl?page=/NPSA/npsa_blast.html>
          + ClustalW P

<https://npsa.lyon.inserm.fr/cgi-bin/npsa_automat.pl?page=/NPSA/npsa_clustalw.html>
          + FASTA P

<https://npsa.lyon.inserm.fr/cgi-bin/npsa_automat.pl?page=/NPSA/npsa_fasta.html>
      o Specialized
          + Annotate <https://hbvdb.lyon.inserm.fr/HBVdb/HBVdbAnnotate>
          + Genotype <https://hbvdb.lyon.inserm.fr/HBVdb/HBVdbGenotype>
          + Resistance <https://hbvdb.lyon.inserm.fr/HBVdb/HBVdbResistance>
  * HBVdb
      o About <https://hbvdb.lyon.inserm.fr/HBVdb/HBVdbAbout>
      o Contact <https://hbvdb.lyon.inserm.fr/HBVdb/HBVdbContact>

Annotate results for OM721312: text entry in EMBL format

```
ID   OM721312; SV 1; circular; genomic DNA; STD; VRL; 3182 BP.
XX
AC   OM721312;
XX
XX
DT   11-FEB-2022 (Rel. 0, Created)
DT   11-FEB-2022 (Rel. 0, Last updated, Version 0)
XX
DE   Hepatitis B Virus genotype D. Complete genome.
XX
KW   HBe; HBc; HBx; LHBs; MHBs; SHBs; Pol; HBSP; complete genome; Sensitive to
drugs.
```

```
XX
OS   Hepatitis B Virus genotype D
OC   Viruses; Retro-transcribing viruses; Hepadnaviridae; Orthohepadnavirus.
XX
RN   1
RP   1-3182
RA   Unknown M;
RL   Submitted (11-FEB-2022) to FR-IBCP-PRABI-ISA.
XX
CC   The data provided in this entry have been computed thanks to
CC   the Hepatitis B Virus Database (HBVdb) annotation alogrithms.
CC   HBVdb is available at http://hbvdb.ibcp.fr.
XX
FH   Key             Location/Qualifiers
FH
FT   source          1..3182
FT                   /mol_type="genomic DNA"
FT                   /organism="Hepatitis B Virus"
FT                   /db_xref="taxon:10407"
FT                   /db_xref="HBVdb:AF121240"
FT                   /PRABI_genotype="n.a.:D:n.a."
FT   CDS             1814..2452
FT                   /PRABI_name="PreC"
FT                   /locus_tag="HBVORF02"
FT                   /codon_start="1"
FT                   /translation="MQLFHLCLIISCSCPTVQASKLCLGWLWGMDIDPYKEFGATVELL
FT                   SFLPSDFFPSVRDLLDTASALYREALESPEHCSPHHTALRQAILCWGELMTLATWVGGN
FT                   LEDPISRDLVVSYVNTNMGLKFRQLLWFHISCLTFGRETVIEYLVSFGVWIRTPPAYRP
FT                   PNAPILSTLPETTVVRRRGRSPRRRTPSPRRRRSQSPRRRRSQSRESQC"
FT   mat_peptide     1871..2275
FT                   /function="HBe/External core antigen coding sequence"
FT                   /locus_tag="HBVORF02"
FT                   /product="External core antigen"
FT                   /PRABI_name="HBe"
FT                   /PRABI_prodft=(pos:20..154, CHAIN, "HBe antigen")
FT   CDS             1901..2452
FT                   /PRABI_name="C"
FT                   /locus_tag="HBVORF12"
FT                   /codon_start="1"
FT                   /translation="MDIDPYKEFGATVELLSFLPSDFFPSVRDLLDTASALYREALESP
FT                   EHCSPHHTALRQAILCWGELMTLATWVGGNLEDPISRDLVVSYVNTNMGLKFRQLLWFH
FT                   ISCLTFGRETVIEYLVSFGVWIRTPPAYRPPNAPILSTLPETTVVRRRGRSPRRRTPSP
FT                   RRRRSQSPRRRRSQSRESQC"
FT   mat_peptide     1901..2449
FT                   /function="Core protein coding sequence"
FT                   /locus_tag="HBVORF12"
FT                   /product="Core protein"
FT                   /PRABI_name="HBc"
FT                   /PRABI_prodft=(pos:1..183, CHAIN, "Core protein")
FT   CDS             1374..1838
```

```
FT                   /PRABI_name="X"
FT                   /locus_tag="HBVORF03"
FT                   /codon_start="1"
FT                   /translation="MAARLCCQLDPARDVLCLRPVGAESRGRPFSGPLGTLSSPSPSAV
FT                   STDHGAHLSLRGLPVCAFSSAGPCALRFTSARRMETTVNAHQFLPKVLHKRTLGLSVMS
FT                   TTDLEAYFKDCLFKDWEELGEEIRLKVFVLGGCRHKLVCAPAPCNFFTSA"
FT   mat_peptide     1374..1835
FT                   /function="X protein coding sequence"
FT                   /locus_tag="HBVORF03"
FT                   /product="X protein"
FT                   /PRABI_name="HBx"
FT                   /PRABI_prodft=(pos:1..154, CHAIN, "X protein")
FT   CDS             join(2848..3182,1..835)
FT                   /PRABI_name="PreS1"
FT                   /locus_tag="HBVORF01"
FT                   /codon_start="1"
FT                   /translation="MGQNLSTSNPLGFFPDHQLDPAFRANTANPDWDFNPNKDTWPDAN
FT                   KVGAGAFGLGFTPPHGGLLGWSPQAQGILQTLPANPPPAATNRQSGRQPTPLSPPLRNT
FT                   HPQAMQWNSTTFHQTLQDPRVRGLYFPAGGSSSGTVNPVPTTVSHISSIFSRIGDPALN
FT                   MENITSGFLGPLLVLQAGFFLLTRILTIPQSLDSWWTSLNFLGGTTVCLGQNSQSPTSN
FT                   HSPTSCPPTCPGYRWMCLRRFIIFLFILLLCLIFLLVLLDYQGMLPVCPLIPGSSTTST
FT                   GPCRTCTTPAQGTSMYPSCCCTKPSDGNCTCIPIPSSWAFGKFLWEWASARFSWLSLLV
FT                   PFVQWFVGLSPTVWLSVIWMMWYWGPSLYSILSPFLPLLPIFFCLWVYI"
FT   mat_peptide     join(2848..3182,1..832)
FT                   /function="PreS1/Large Surface protein coding sequence"
FT                   /locus_tag="HBVORF01"
FT                   /product="PreS1 Surface protein"
FT                   /PRABI_name="LHBs"
FT                   /PRABI_prodft=(pos:1..389, CHAIN, "Large Surface protein")
FT   CDS             join(3172..3182,1..835)
FT                   /PRABI_name="PreS2"
FT                   /locus_tag="HBVORF11"
FT                   /codon_start="1"
FT                   /translation="MQWNSTTFHQTLQDPRVRGLYFPAGGSSSGTVNPVPTTVSHISSI
FT                   FSRIGDPALNMENITSGFLGPLLVLQAGFFLLTRILTIPQSLDSWWTSLNFLGGTTVCL
FT                   GQNSQSPTSNHSPTSCPPTCPGYRWMCLRRFIIFLFILLLCLIFLLVLLDYQGMLPVCP
FT                   LIPGSSTTSTGPCRTCTTPAQGTSMYPSCCCTKPSDGNCTCIPIPSSWAFGKFLWEWAS
FT                   ARFSWLSLLVPFVQWFVGLSPTVWLSVIWMMWYWGPSLYSILSPFLPLLPIFFCLWVYI
FT                   "
FT   mat_peptide     join(3172..3182,1..832)
FT                   /function="PreS2/Middle Surface protein coding sequence"
FT                   /locus_tag="HBVORF11"
FT                   /product="PreS2 Surface protein"
FT                   /PRABI_name="MHBs"
FT                   /PRABI_prodft=(pos:1..281, CHAIN, "Middle Surface protein")
FT   CDS             155..835
FT                   /PRABI_name="S"
FT                   /locus_tag="HBVORF21"
FT                   /codon_start="1"
FT                   /translation="MENITSGFLGPLLVLQAGFFLLTRILTIPQSLDSWWTSLNFLGGT
```

```
FT                   TVCLGQNSQSPTSNHSPTSCPPTCPGYRWMCLRRFIIFLFILLLCLIFLLVLLDYQGML
FT                   PVCPLIPGSSTTSTGPCRTCTTPAQGTSMYPSCCCTKPSDGNCTCIPIPSSWAFGKFLW
FT                   EWASARFSWLSLLVPFVQWFVGLSPTVWLSVIWMMWYWGPSLYSILSPFLPLLPIFFCL
FT                   WVYI"
FT   mat_peptide     155..832
FT                   /function="S protein coding sequence"
FT                   /locus_tag="HBVORF21"
FT                   /product="Surface protein S"
FT                   /PRABI_name="SHBs"
FT                   /PRABI_prodft=(pos:1..226, CHAIN, "Small Surface protein")
FT   CDS             join(2307..3182,1..1623)
FT                   /PRABI_name="P"
FT                   /locus_tag="HBVORF13"
FT                   /codon_start="1"
FT                   /translation="MPLSYQHFRRLLLLDDEAGPLEEELPRLADEGLNRRVAEDLNLGN
FT                   LNVSIPWTHKVGNFTGLYSSTVPVFNPHWKTPSFPNIHLHQDIIKKCEHFVGPLTVNEK
FT                   RRLQLIMPARFYPKVTKYLPLDKGIKPYYPEHLVNHYFQTRHYLHTLWKAGILYKRETT
FT                   HSASFYGSPYSWEQELQHGAESFHQQSSGILSRPPVGSSLQSKHRKSRLGLQSQQGHLA
FT                   RRQQGRSWSIRAGIHPTARRPFGVEPSGSGHTTNLASKSASCRYQSPVRKAAYPSVSTF
FT                   EKHSSSGHAVELHNLPPNSARSQSERPVFPCWWLQFRNSKPCSDYCLSHIVNLLEDWGP
FT                   CAEHGEHHIRIPRTPARVTGGVFLVDKNPHNTAESRLVVDFSQFSRGNYRVSWPKFAVP
FT                   NLQSLTNLLSSNLSWLSLDVSAAFYHLPLHPAAMPHLLVGSSGLSRYVARLSSNSRIFN
FT                   HQHGTMQNLHDSCSRNLYVSLLLLYQTFGRKLHLYSHPIILGFRKIPMGVGLSPFLLAQ
FT                   FTSAICSVVRRAFPHCLAFSYMDDVVLGAKSVQHLESIFTAVTNFLLSLGIHLNPNKTK
FT                   RWGYSLHFMGYVIGCYGSLPQDHIIQKIKECFRKLPVNRPIDWKVCQRIVGLLGFAAPF
FT                   TQCGYPALMPLYACIQSKLAFTFSPTYKAFLCKQYLNLYPVARQRPGLCQVFADATPTG
FT                   WGLVMGHQRMRGTFSAPLPIHTAELLAACFARSRSGANILGTDNSVVLSRKYTSFPWLL
FT                   GCAANWILRGTSFVYVPSALNPADDPSRGRLGLSRPLLRLPFRPTTGRTSLYADSPSVP
FT                   SHLPDRVHFASPLHVAWRPP"
FT   mat_peptide     join(2307..3182,1..1620)
FT                   /function="DNA-polymerase/Reverse Transcriptase coding
FT                   sequence"
FT                   /locus_tag="HBVORF13"
FT                   /product="Polymerase/Reverse Transcriptase"
FT                   /PRABI_name="Pol"
FT                   /PRABI_prodft=(pos:1..832, CHAIN, "Polymerase/Reverse
FT                   transcriptase")
FT                   /PRABI_prodft=(pos:1..180, DOMAIN, "Terminal Protein (TP)/
FT                   Primase domain")
FT                   /PRABI_prodft=(pos:181..335, DOMAIN, "Spacer")
FT                   /PRABI_prodft=(pos:336..679, DOMAIN, "Reverse Transcriptase
FT                   (RT) domain")
FT                   /PRABI_prodft=(pos:418..418, ACT_SITE, "RT catalytic Asp")
FT                   /PRABI_prodft=(pos:540..540, ACT_SITE, "RT catalytic Asp")
FT                   /PRABI_prodft=(pos:541..541, ACT_SITE, "RT catalytic Asp")
FT                   /PRABI_prodft=(pos:680..832, DOMAIN, "Ribonuclease H
FT                   (RNaseH) domain")
FT                   /PRABI_prodft=(pos:689..689, ACT_SITE, "RNaseH catalytic
FT                   Asp")
FT                   /PRABI_prodft=(pos:718..718, ACT_SITE, "RNaseH catalytic
```

```
FT                   Glu")
FT                   /PRABI_prodft=(pos:737..737, ACT_SITE, "RNaseH catalytic
FT                   Asp")
FT   CDS             join(2307..2447,489..683)
FT                   /PRABI_name="SP"
FT                   /locus_tag="HBVORF04"
FT                   /codon_start="1"
FT                   /translation="MPLSYQHFRRLLLLDDEAGPLEEELPRLADEGLNRRVAEDLNLGN
FT                   LNDLQPPARDHAEPARLLLKEPLCIPPAAVPNLRTEIAPVFPSHHPGLSENSYGSGPQP
FT                   VSPGSVY"
FT   mat_peptide     join(2307..2447,489..680)
FT                   /function="HBSP coding sequence"
FT                   /locus_tag="HBVORF04"
FT                   /product="Hepatitis B Spliced Protein"
FT                   /PRABI_name="HBSP"
FT                   /PRABI_prodft=(pos:1..111, CHAIN, "HBV Spliced Protein")
XX
SQ   Sequence 3182 BP; 727 A; 860 C; 692 G; 903 T; 0 other;
     ctccacaacc ttccaccaaa ctctgcaaga tcccagagtg agaggcctgt atttccctgc        60
     tggtggctcc agttcaggaa cagtaaaccc tgttccgact actgtctctc acatatcgtc       120
     aatcttctcg aggattgggg accctgcgct gaacatggag aacatcacat caggattcct       180
     aggacccctg ctcgtgttac aggcggggtt tttcttgttg acaagaatcc tcacaatacc       240
     gcagagtcta gactcgtggt ggacttctct caattttcta gggggaacta ccgtgtgtct       300
     tggccaaaat tcgcagtccc caacctccaa tcactcacca acctcctgtc ctccaacttg       360
     tcctggttat cgctggatgt gtctgcggcg ttttatcatc ttcctcttca tcctgctgct       420
     atgcctcatc ttcttgttgg ttcttctgga ctatcaaggt atgttgcccg tttgtcctct       480
     aattccagga tcttcaacca ccagcacggg accatgcaga acctgcacga ctcctgctca       540
     aggaacctct atgtatccct cctgctgctg taccaaacct tcggacggaa attgcacctg       600
     tattcccatc ccatcatcct gggctttcgg aaaattccta tgggagtggg cctcagcccg       660
     tttctcctgg ctcagtttac tagtgccatt tgttcagtgg ttcgtagggc tttcccccac       720
     tgtttggctt tcagttatat ggatgatgtg gtattggggg ccaagtctgt acagcatctt       780
     gagtccattt ttaccgctgt taccaatttt cttttgtctc tgggtataca tttaaaccct       840
     aacaaaacaa aaagatgggg ttactcttta catttcatgg gctatgtcat tggatgttat       900
     gggtcattgc cacaagatca catcatacag aaaatcaaag aatgttttag aaaacttcct       960
     gttaacaggc ctattgattg gaaagtctgt caacgtattg tgggtctttt gggttttgct      1020
     gccccttta cacaatgtgg ttatcctgct ttaatgccct tgtatgcatg tattcaatct      1080
     aagctggctt tcactttctc gccaacttac aaggcctttc tgtgtaaaca atacctgaac      1140
     ctttaccccg ttgcccggca acggccaggt ctgtgccaag tgtttgctga cgcaaccccc      1200
     actggctggg gcttggtcat gggccatcag cgcatgcgtg gaacctttc ggctcctctg      1260
     ccgatccata ctgcggaact cctagccgct tgttttgctc gcagcaggtc tggagcaaac      1320
     attctcggga cggataactc tgttgttctc tcccgcaaat atacgtcgtt tccatggctg      1380
     ctaggctgtg ctgccaactg gatcctgcgc gggacgtcct ttgtttacgt cccgtcggcg      1440
     ctgaatcccg cggacgaccc ttctcggggc cgcttgggac tctctcgtcc ccttctccgt      1500
     ctgccgtttc gaccgaccac ggggcgcacc tctctttacg cggactcccc gtctgtgcct      1560
     tctcatctgc cggaccgtgt gcacttcgct tcacctctgc acgtcgcatg gagaccaccg      1620
     tgaacgccca ccaattcttg cccaaggtct tacataagag gactcttgga ctctctgtga      1680
     tgtcaacgac cgaccttgag gcatacttca aagactgttt gtttaaagac tgggaggagt      1740
     tgggggagga gattagatta aaggtctttg tattaggagg ctgtaggcat aaattggtct      1800
     gcgcaccagc accatgcaac tttttcacct ctgcctaatc atctcttgtt catgtcctac      1860
     tgttcaagcc tccaagctgt gccttgggtg gctttggggc atggacattg atccttataa      1920
```

```
     agaatttgga gctactgtgg agttactctc atttttgcct tctgacttct ttccttcagt      1980
     acgagatctt ctagataccg cctcagctct atatcgggaa gccttagagt ctcctgagca      2040
     ttgttcacct caccatactg cactcaggca agcaattctt tgctgggggg aactaatgac      2100
     tttagccacc tgggtgggtg gtaatttgga agatccaata tccagggacc tagtagtcag      2160
     ttatgttaac actaatatgg gcctaaagtt caggcaacta ttgtggtttc acatttcttg      2220
     tctcactttt ggaagagaaa cggtcataga gtatttggtg tctttcggag tgtggattcg      2280
     cactcctcca gcttatagac caccaaatgc ccctatctta tcaacacttc cggagactac      2340
     tgttgttaga cgacgaggca ggtcccctag aagaagaact ccctcgcctc gcagacgaag      2400
     gtctcaatcg ccgcgtcgca gaagatctca atctcgggaa tctcaatgtt agtattcctt      2460
     ggactcataa ggtgggaaac tttacggggc tttattcttc tactgttcct gtctttaacc      2520
     ctcattggaa aacaccctct ttcccaata tacacttaca ccaagacatt atcaaaaaat      2580
     gtgaacattt tgtaggccca ctcacagtca atgagaaaag aagactgcaa ttgattatgc      2640
     ctgctaggtt ctatccaaag gttaccaaat atttgccatt ggataagggt attaaacctt      2700
     attatccaga acatctagtt aatcattact tccaaaccag acattattta cacactctat      2760
     ggaaggcggg tatattatat aagagagaaa caacacatag cgcctcattt tatgggtcac      2820
     catattcttg ggaacaagag ctacagcatg gggcagaatc tttccaccag caatcctctg      2880
     ggattctttc ccgaccacca gttggatcca gccttcagag caaacaccgc aaatccagat      2940
     tgggacttca atcccaacaa ggacacctgg ccagacgcca acaaggtagg agctggagca      3000
     ttcgggctgg gattcacccc accgcacgga ggccttttgg ggtggagccc tcaggctcag      3060
     ggcatactac aaaccttgcc agcaaatccg cctcctgccg ctaccaatcg ccagtcagga      3120
     aggcagccta cccctctgtc tccacctttg agaaacactc atcctcaggc catgcagtgg      3180
     aa                                                                     3182
//
```

© 1998-2022 Centre de Recherche en Cancerologie de Lyon logo <http://www.crcl.fr/> Pole Rhone-Alpes de BioInformatique logo <http://prabi.ibcp.fr/> Centre National de la Recherche Scientifique logo <http://www.cnrs.fr/> Institut national de la sante et de la recherche medicale logo <http://www.inserm.fr/> Universite Claude Bernard Lyon 1 logo <http://www.univ-lyon1.fr/> Legal notice <https://hbvdb.lyon.inserm.fr/HBVdb/HBVdbAbout#legalnotice>

HBVdb

* Home <https://hbvdb.lyon.inserm.fr/HBVdb/HBVdbIndex>
* HBV
    o Genome <https://hbvdb.lyon.inserm.fr/HBVdb/HBVdbGenome>
    o Nomenclature <https://hbvdb.lyon.inserm.fr/HBVdb/HBVdbNomenclature>
    o Proteins
        + Core
          <https://hbvdb.lyon.inserm.fr/HBVdb/HBVdbProteins?protein=Core>
        + HBx
          <https://hbvdb.lyon.inserm.fr/HBVdb/HBVdbProteins?protein=HBx>
        + Surface
          <https://hbvdb.lyon.inserm.fr/HBVdb/HBVdbProteins?protein=Surface>
        + Polymerase
          <https://hbvdb.lyon.inserm.fr/HBVdb/HBVdbProteins?protein=Polymerase>
* Query
    o Dataset
        + Nucleotide
          <https://hbvdb.lyon.inserm.fr/HBVdb/HBVdbDataset?seqtype=0>
        + Protein
          <https://hbvdb.lyon.inserm.fr/HBVdb/HBVdbDataset?seqtype=2>
* Analysis
    o Generic N
        + Blast N

<https://npsa.lyon.inserm.fr/cgi-bin/npsa_automat.pl?page=/NPSA/npsa_blastan.html>
        + ClustalW N

<https://npsa.lyon.inserm.fr/cgi-bin/npsa_automat.pl?page=/NPSA/npsa_clustalwan.html
>
        + FASTA N

<https://npsa.lyon.inserm.fr/cgi-bin/npsa_automat.pl?page=/NPSA/npsa_fastaan.html>
    o Generic P
        + Blast P

<https://npsa.lyon.inserm.fr/cgi-bin/npsa_automat.pl?page=/NPSA/npsa_blast.html>
        + ClustalW P

<https://npsa.lyon.inserm.fr/cgi-bin/npsa_automat.pl?page=/NPSA/npsa_clustalw.html>
        + FASTA P

<https://npsa.lyon.inserm.fr/cgi-bin/npsa_automat.pl?page=/NPSA/npsa_fasta.html>
    o Specialized
        + Annotate <https://hbvdb.lyon.inserm.fr/HBVdb/HBVdbAnnotate>
        + Genotype <https://hbvdb.lyon.inserm.fr/HBVdb/HBVdbGenotype>
        + Resistance <https://hbvdb.lyon.inserm.fr/HBVdb/HBVdbResistance>
* HBVdb
    o About <https://hbvdb.lyon.inserm.fr/HBVdb/HBVdbAbout>
    o Contact <https://hbvdb.lyon.inserm.fr/HBVdb/HBVdbContact>

o Help
+ Annotate <https://hbvdb.lyon.inserm.fr/HBVdb/HBVdbHelp?userhelp=Annotate>
+ Genotype <https://hbvdb.lyon.inserm.fr/HBVdb/HBVdbHelp?userhelp=Genotype>
+ Home <https://hbvdb.lyon.inserm.fr/HBVdb/HBVdbHelp>
+ Resistance <https://hbvdb.lyon.inserm.fr/HBVdb/HBVdbHelp?userhelp=Resistance>

o News <https://hbvdb.lyon.inserm.fr/HBVdb/HBVdbNews>
o Statistics <https://hbvdb.lyon.inserm.fr/HBVdb/HBVdbStats>

* Links

o PRABI
+ databases
# BCL2DB <https://bcl2db.lyon.inserm.fr/>
# BYKdb <https://bykdb.lyon.inserm.fr/>
# euHCVdb <https://euhcvdb.lyon.inserm.fr/>
+ centers
# all <http://www.prabi.fr/>
Gerland <https://prabi.lyon.inserm.fr/>
+ tools
# GENO3D <https://geno3d.lyon.inserm.fr/>
# NPS@ <https://npsa.lyon.inserm.fr/>
# QueBio <https://quebio.lyon.inserm.fr/>

o external
+ databases
# ENA <http://www.ebi.ac.uk/ena/>
# PDB <http://www.wwpdb.org/>
# UniProtKB <http://www.uniprot.org/>
+ centers
# EBI <http://www.ebi.ac.uk/>
# NCBI <http://www.ncbi.nlm.nih.gov/>
# RCSB <http://www.pdb.org/>
# SIB <http://www.isb-sib.ch/>

Annotate results for OM721313: text entry in EMBL format

```
ID   OM721313; SV 1; circular; genomic DNA; STD; VRL; 3182 BP.
XX
AC   OM721313;
XX
XX
DT   11-FEB-2022 (Rel. 0, Created)
DT   11-FEB-2022 (Rel. 0, Last updated, Version 0)
XX
DE   Hepatitis B Virus genotype D. Complete genome.
XX
KW   HBe; HBc; HBx; LHBs; MHBs; SHBs; Pol; HBSP; complete genome; Sensitive to
drugs.
```

```
XX
OS   Hepatitis B Virus genotype D
OC   Viruses; Retro-transcribing viruses; Hepadnaviridae; Orthohepadnavirus.
XX
RN   1
RP   1-3182
RA   Unknown M;
RL   Submitted (11-FEB-2022) to FR-IBCP-PRABI-ISA.
XX
CC   The data provided in this entry have been computed thanks to
CC   the Hepatitis B Virus Database (HBVdb) annotation alogrithms.
CC   HBVdb is available at http://hbvdb.ibcp.fr.
XX
FH   Key             Location/Qualifiers
FH
FT   source          1..3182
FT                   /mol_type="genomic DNA"
FT                   /organism="Hepatitis B Virus"
FT                   /db_xref="taxon:10407"
FT                   /db_xref="HBVdb:AF121240"
FT                   /PRABI_genotype="n.a.:D:n.a."
FT   CDS             1814..2452
FT                   /PRABI_name="PreC"
FT                   /locus_tag="HBVORF02"
FT                   /codon_start="1"
FT                   /translation="MQLFHLCLIISCSCPTVQASKLCLGWLWGMDIDPYKEFGATVELL
FT                   SFLPSDFFPSVRDLLDTASALYREALESPEHCSPHHTALRQAILCWGELMTLATWVGGN
FT                   LEDPISRDLVVSYVNTNMGLKFRQLLWFHISCLTFGRETVIEYLVSFGVWIRTPPAYRP
FT                   PNAPILSTLPETTVVRRRGRSPRRRTPSPRRRRSQSPRRRRSQSRESQC"
FT   mat_peptide     1871..2275
FT                   /function="HBe/External core antigen coding sequence"
FT                   /locus_tag="HBVORF02"
FT                   /product="External core antigen"
FT                   /PRABI_name="HBe"
FT                   /PRABI_prodft=(pos:20..154, CHAIN, "HBe antigen")
FT   CDS             1901..2452
FT                   /PRABI_name="C"
FT                   /locus_tag="HBVORF12"
FT                   /codon_start="1"
FT                   /translation="MDIDPYKEFGATVELLSFLPSDFFPSVRDLLDTASALYREALESP
FT                   EHCSPHHTALRQAILCWGELMTLATWVGGNLEDPISRDLVVSYVNTNMGLKFRQLLWFH
FT                   ISCLTFGRETVIEYLVSFGVWIRTPPAYRPPNAPILSTLPETTVVRRRGRSPRRRTPSP
FT                   RRRRSQSPRRRRSQSRESQC"
FT   mat_peptide     1901..2449
FT                   /function="Core protein coding sequence"
FT                   /locus_tag="HBVORF12"
FT                   /product="Core protein"
FT                   /PRABI_name="HBc"
FT                   /PRABI_prodft=(pos:1..183, CHAIN, "Core protein")
FT   CDS             1374..1838
```

```
FT                   /PRABI_name="X"
FT                   /locus_tag="HBVORF03"
FT                   /codon_start="1"
FT                   /translation="MAARLCCQLDPARDVLCLRPVGAESRGRPFSGPLGTLSSPSPSAV
FT                   STDHGAHLSLRGLPVCAFSSAGPCALRFTSARRMETTVNAHQFLPKVLHKRTLGLSVMS
FT                   TTDLEAYFKDCLFKDWEELGEEIRLKVFVLGGCRHKLVCAPAPCNFFTSA"
FT   mat_peptide     1374..1835
FT                   /function="X protein coding sequence"
FT                   /locus_tag="HBVORF03"
FT                   /product="X protein"
FT                   /PRABI_name="HBx"
FT                   /PRABI_prodft=(pos:1..154, CHAIN, "X protein")
FT   CDS             join(2848..3182,1..835)
FT                   /PRABI_name="PreS1"
FT                   /locus_tag="HBVORF01"
FT                   /codon_start="1"
FT                   /translation="MGQNLSTSNPLGFFPDHQLDPAFRANTANPDWDFNPNKDTWPDAN
FT                   KVGAGAFGLGFTPPHGGLLGWSPQAQGILQTLPANPPPASTNRQSGRQPTPLSPPLRNT
FT                   HPQAMQWNSTTFHQTLQDPRVRGLYFPAGGSSSGTVNPVPTTVSHISSIFSRIGDPALN
FT                   MENITSGFLGPLLVLQAGFFLLTRILTIPQSLDSWWTSLNFLGGTTVCLGQNSQSPTSN
FT                   HSPTSCPPTCPGYRWMCLRRFIIFLFILLLCLIFLLVLLDYQGMLPVCPLIPGSSTTST
FT                   GPCRTCTTPAQGTSMYPSCCCTKPSDGNCTCIPIPSSWAFGKFLWEWASARFSWLSLLV
FT                   PFVQWFVGLSPTVWLSVIWMMWYWGPSLYSILSPFLPLLPIFFCLWVYI"
FT   mat_peptide     join(2848..3182,1..832)
FT                   /function="PreS1/Large Surface protein coding sequence"
FT                   /locus_tag="HBVORF01"
FT                   /product="PreS1 Surface protein"
FT                   /PRABI_name="LHBs"
FT                   /PRABI_prodft=(pos:1..389, CHAIN, "Large Surface protein")
FT   CDS             join(3172..3182,1..835)
FT                   /PRABI_name="PreS2"
FT                   /locus_tag="HBVORF11"
FT                   /codon_start="1"
FT                   /translation="MQWNSTTFHQTLQDPRVRGLYFPAGGSSSGTVNPVPTTVSHISSI
FT                   FSRIGDPALNMENITSGFLGPLLVLQAGFFLLTRILTIPQSLDSWWTSLNFLGGTTVCL
FT                   GQNSQSPTSNHSPTSCPPTCPGYRWMCLRRFIIFLFILLLCLIFLLVLLDYQGMLPVCP
FT                   LIPGSSTTSTGPCRTCTTPAQGTSMYPSCCCTKPSDGNCTCIPIPSSWAFGKFLWEWAS
FT                   ARFSWLSLLVPFVQWFVGLSPTVWLSVIWMMWYWGPSLYSILSPFLPLLPIFFCLWVYI
FT                   "
FT   mat_peptide     join(3172..3182,1..832)
FT                   /function="PreS2/Middle Surface protein coding sequence"
FT                   /locus_tag="HBVORF11"
FT                   /product="PreS2 Surface protein"
FT                   /PRABI_name="MHBs"
FT                   /PRABI_prodft=(pos:1..281, CHAIN, "Middle Surface protein")
FT   CDS             155..835
FT                   /PRABI_name="S"
FT                   /locus_tag="HBVORF21"
FT                   /codon_start="1"
FT                   /translation="MENITSGFLGPLLVLQAGFFLLTRILTIPQSLDSWWTSLNFLGGT
```

```
FT                   TVCLGQNSQSPTSNHSPTSCPPTCPGYRWMCLRRFIIFLFILLLCLIFLLVLLDYQGML
FT                   PVCPLIPGSSTTSTGPCRTCTTPAQGTSMYPSCCCTKPSDGNCTCIPIPSSWAFGKFLW
FT                   EWASARFSWLSLLVPFVQWFVGLSPTVWLSVIWMMWYWGPSLYSILSPFLPLLPIFFCL
FT                   WVYI"
FT   mat_peptide     155..832
FT                   /function="S protein coding sequence"
FT                   /locus_tag="HBVORF21"
FT                   /product="Surface protein S"
FT                   /PRABI_name="SHBs"
FT                   /PRABI_prodft=(pos:1..226, CHAIN, "Small Surface protein")
FT   CDS             join(2307..3182,1..1623)
FT                   /PRABI_name="P"
FT                   /locus_tag="HBVORF13"
FT                   /codon_start="1"
FT                   /translation="MPLSYQHFRRLLLLDDEAGPLEEELPRLADEGLNRRVAEDLNLGN
FT                   LNVSIPWTHKVGNFTGLYSSTVPVFNPHWKTPSFPNIHLHQDIIKKCEQFVGPLTVNEK
FT                   RRLQLIMPARFYPKVTKYLPLDKGIKPYYPEHLVNHYFQTRHYLHTLWKAGILYKRETT
FT                   HSASFCGSPYSWEQELQHGAESFHQQSSGILSRPPVGSSLQSKHRKSRLGLQSQQGHLA
FT                   RRQQGRSWSIRAGIHPTARRPFGVEPSGSGHTTNLASKSASCLYQSPVRKAAYPSVSTF
FT                   EKHSSSGHAVELHNLPPNSARSQSERPVFPCWWLQFRNSKPCSDYCLSHIVNLLEDWGP
FT                   CAEHGEHHIRIPRTPARVTGGVFLVDKNPHNTAESRLVVDFSQFSRGNYRVSWPKFAVP
FT                   NLQSLTNLLSSNLSWLSLDVSAAFYHLPLHPAAMPHLLVGSSGLSRYVARLSSNSRIFN
FT                   HQHGTMQNLHDSCSRNLYVSLLLLYQTFGRKLHLYSHPIILGFRKIPMGVGLSPFLLAQ
FT                   FTSAICSVVRRAFPHCLAFSYMDDVVLGAKSVQHLESIFTAVTNFLLSLGIHLNPNKTK
FT                   RWGYSLHFMGYVIGCYGSLPQDHIIQKIKECFRKLPVNRPIDWKVCQRIVGLLGFAAPF
FT                   TQCGYPALMPLYACIQSKLAFTFSPTYKAFLCKQYLNLYPVARQRPGLCQVFADATPTG
FT                   WGLVMGHQRMRGTFSAPLPIHTAELLAACFARSRSGANILGTDNSVVLSRKYTSFPWLL
FT                   GCAANWILRGTSFVYVPSALNPADDPSRGRLGLSRPLLRLPFRPTTGRTSLYADSPSVP
FT                   SHLPDRVHFASPLHVAWRPP"
FT   mat_peptide     join(2307..3182,1..1620)
FT                   /function="DNA-polymerase/Reverse Transcriptase coding
FT                   sequence"
FT                   /locus_tag="HBVORF13"
FT                   /product="Polymerase/Reverse Transcriptase"
FT                   /PRABI_name="Pol"
FT                   /PRABI_prodft=(pos:1..832, CHAIN, "Polymerase/Reverse
FT                   transcriptase")
FT                   /PRABI_prodft=(pos:1..180, DOMAIN, "Terminal Protein (TP)/
FT                   Primase domain")
FT                   /PRABI_prodft=(pos:181..335, DOMAIN, "Spacer")
FT                   /PRABI_prodft=(pos:336..679, DOMAIN, "Reverse Transcriptase
FT                   (RT) domain")
FT                   /PRABI_prodft=(pos:418..418, ACT_SITE, "RT catalytic Asp")
FT                   /PRABI_prodft=(pos:540..540, ACT_SITE, "RT catalytic Asp")
FT                   /PRABI_prodft=(pos:541..541, ACT_SITE, "RT catalytic Asp")
FT                   /PRABI_prodft=(pos:680..832, DOMAIN, "Ribonuclease H
FT                   (RNaseH) domain")
FT                   /PRABI_prodft=(pos:689..689, ACT_SITE, "RNaseH catalytic
FT                   Asp")
FT                   /PRABI_prodft=(pos:718..718, ACT_SITE, "RNaseH catalytic
```

```
FT                   Glu")
FT                   /PRABI_prodft=(pos:737..737, ACT_SITE, "RNaseH catalytic
FT                   Asp")
FT   CDS             join(2307..2447,489..683)
FT                   /PRABI_name="SP"
FT                   /locus_tag="HBVORF04"
FT                   /codon_start="1"
FT                   /translation="MPLSYQHFRRLLLLDDEAGPLEEELPRLADEGLNRRVAEDLNLGN
FT                   LNDLQPPARDHAEPARLLLKEPLCIPPAAVPNLRTEIAPVFPSHHPGLSENSYGSGPQP
FT                   VSPGSVY"
FT   mat_peptide     join(2307..2447,489..680)
FT                   /function="HBSP coding sequence"
FT                   /locus_tag="HBVORF04"
FT                   /product="Hepatitis B Spliced Protein"
FT                   /PRABI_name="HBSP"
FT                   /PRABI_prodft=(pos:1..111, CHAIN, "HBV Spliced Protein")
XX
SQ   Sequence 3182 BP; 727 A; 861 C; 692 G; 902 T; 0 other;
     ctccacaacc ttccaccaaa ctctgcaaga tcccagagtg agaggcctgt atttccctgc        60
     tggtggctcc agttcaggaa cagtaaaccc tgttccgact actgtctctc acatatcgtc       120
     aatcttctcg aggattgggg accctgcgct gaacatggag aacatcacat caggattcct       180
     aggacccctg ctcgtgttac aggcggggtt tttcttgttg acaagaatcc tcacaatacc       240
     gcagagtcta gactcgtggt ggacttctct caattttcta gggggaacta ccgtgtgtct       300
     tggccaaaat tcgcagtccc caacctccaa tcactcacca acctcctgtc ctccaacttg       360
     tcctggttat cgctggatgt gtctgcggcg ttttatcatc ttcctcttca tcctgctgct       420
     atgcctcatc ttcttgttgg ttcttctgga ctatcaaggt atgttgcccg tttgtcctct       480
     aattccagga tcttcaacca ccagcacggg accatgcaga acctgcacga ctcctgctca       540
     aggaacctct atgtatccct cctgctgctg taccaaacct tcggacggaa attgcacctg       600
     tattcccatc ccatcatcct gggctttcgg aaaattccta tgggagtggg cctcagcccg       660
     tttctcctgg ctcagtttac tagtgccatt tgttcagtgg ttcgtagggc tttcccccac       720
     tgtttggctt tcagttatat ggatgatgtg gtattggggg ccaagtctgt acagcatctt       780
     gagtccattt ttaccgctgt taccaatttt cttttgtctc tgggtataca tttaaaccct       840
     aacaaaacaa aaagatgggg ttactcttta catttcatgg gctatgtcat tggatgttat       900
     gggtcattgc cacaagatca catcatacag aaaatcaaag aatgttttag aaaacttcct       960
     gttaacaggc ctattgattg gaaagtctgt caacgtattg tgggtctttt gggttttgct      1020
     gccccttttta cacaatgtgg ttatcctgct ttaatgccct tgtatgcatg tattcaatct      1080
     aagctggctt tcactttctc gccaacttac aaggcctttc tgtgtaaaca atacctgaac      1140
     ctttaccccg ttgcccggca acggccaggt ctgtgccaag tgtttgctga cgcaaccccc      1200
     actggctggg gcttggtcat gggccatcag cgcatgcgtg gaaccttttc ggctcctctg      1260
     ccgatccata ctgcggaact cctagccgct tgttttgctc gcagcaggtc tggagcaaac      1320
     attctcggga cggataactc tgttgttctc tcccgcaaat atacgtcgtt tccatggctg      1380
     ctaggctgtg ctgccaactg gatcctgcgc gggacgtcct ttgtttacgt cccgtcggcg      1440
     ctgaatcccg cggacgaccc ttctcggggc cgcttgggac tctctcgtcc ccttctccgt      1500
     ctgccgtttc gaccgaccac ggggcgcacc tctctttacg cggactcccc gtctgtgcct      1560
     tctcatctgc cggaccgtgt gcacttcgct tcacctctgc acgtcgcatg gagaccaccg      1620
     tgaacgccca ccaattcttg cccaaggtct tacataagag gactcttgga ctctctgtga      1680
     tgtcaacgac cgaccttgag gcatacttca aagactgttt gtttaaagac tgggaggagt      1740
     tgggggagga gattagatta aaggtctttg tattaggagg ctgtaggcat aaattggtct      1800
     gcgcaccagc accatgcaac tttttcacct ctgcctaatc atctcttgtt catgtcctac      1860
     tgttcaagcc tccaagctgt gccttgggtg gctttggggc atggacattg atccttataa      1920
```

```
     agaatttgga gctactgtgg agttactctc atttttgcct tctgacttct ttccttcagt     1980
     acgagatctt ctagataccg cctcagctct atatcgggaa gccttagagt ctcctgagca     2040
     ttgttcacct caccatactg cactcaggca agcaattctt tgctgggggg aactaatgac     2100
     tttagccacc tgggtgggtg gcaatttgga agatccaata tccagggacc tagtagtcag     2160
     ttatgttaac actaatatgg gcctaaagtt caggcaacta ttgtggtttc acatttcttg     2220
     tctcactttt ggaagagaaa cggtcataga gtatttggtg tctttcggag tgtggattcg     2280
     cactcctcca gcttatagac caccaaatgc ccctatctta tcaacacttc cggagactac     2340
     tgttgttaga cgacgaggca ggtcccctag aagaagaact ccctcgcctc gcagacgaag     2400
     gtctcaatcg ccgcgtcgca gaagatctca atctcgggaa tctcaatgtt agtattcctt     2460
     ggactcataa ggtgggaaac tttacggggc tttattcttc tactgttcct gtctttaacc     2520
     ctcattggaa aacaccctct tttcccaata tacacttaca ccaagacatt atcaaaaaat     2580
     gtgaacaatt tgtaggccca ctcacagtca atgagaaaag aagactgcaa ttgattatgc     2640
     ctgctaggtt ctatccaaag gttaccaaat atttgccatt ggataagggt attaaacctt     2700
     attatccaga acatctagtt aatcattact tccaaaccag acattattta cacactctat     2760
     ggaaggcggg tatattatat aagagagaaa caacacatag cgcctcattt tgtgggtcac     2820
     catattcttg ggaacaagag ctacagcatg gggcagaatc tttccaccag caatcctctg     2880
     ggattctttc ccgaccacca gttggatcca gccttcagag caaacaccgc aaatccagat     2940
     tgggacttca atcccaacaa ggacacctgg ccagacgcca acaaggtagg agctggagca     3000
     ttcgggctgg gattcacccc accgcacgga ggccttttgg ggtggagccc tcaggctcag     3060
     ggcatactac aaaccttgcc agcaaatccg cctcctgcct ctaccaatcg ccagtcagga     3120
     aggcagccta cccctctgtc tccacctttg agaaacactc atcctcaggc catgcagtgg     3180
     aa                                                                    3182
//
```

© 1998-2022 Centre de Recherche en Cancerologie de Lyon logo <http://www.crcl.fr/> Pole Rhone-Alpes de BioInformatique logo <http://prabi.ibcp.fr/> Centre National de la Recherche Scientifique logo <http://www.cnrs.fr/> Institut national de la sante et de la recherche medicale logo <http://www.inserm.fr/> Universite Claude Bernard Lyon 1 logo <http://www.univ-lyon1.fr/> Legal notice <https://hbvdb.lyon.inserm.fr/HBVdb/HBVdbAbout#legalnotice>

HBVdb

* Home <https://hbvdb.lyon.inserm.fr/HBVdb/HBVdbIndex>
* HBV
    o Genome <https://hbvdb.lyon.inserm.fr/HBVdb/HBVdbGenome>
    o Nomenclature <https://hbvdb.lyon.inserm.fr/HBVdb/HBVdbNomenclature>
    o Proteins
        + Core <https://hbvdb.lyon.inserm.fr/HBVdb/HBVdbProteins?protein=Core>
        + HBx <https://hbvdb.lyon.inserm.fr/HBVdb/HBVdbProteins?protein=HBx>
        + Surface <https://hbvdb.lyon.inserm.fr/HBVdb/HBVdbProteins?protein=Surface>
        + Polymerase <https://hbvdb.lyon.inserm.fr/HBVdb/HBVdbProteins?protein=Polymerase>
* Query
    o Dataset
        + Nucleotide <https://hbvdb.lyon.inserm.fr/HBVdb/HBVdbDataset?seqtype=0>
        + Protein <https://hbvdb.lyon.inserm.fr/HBVdb/HBVdbDataset?seqtype=2>
* Analysis
    o Generic N
        + Blast N <https://npsa.lyon.inserm.fr/cgi-bin/npsa_automat.pl?page=/NPSA/npsa_blastan.html>
        + ClustalW N <https://npsa.lyon.inserm.fr/cgi-bin/npsa_automat.pl?page=/NPSA/npsa_clustalwan.html>
        + FASTA N <https://npsa.lyon.inserm.fr/cgi-bin/npsa_automat.pl?page=/NPSA/npsa_fastaan.html>
    o Generic P
        + Blast P <https://npsa.lyon.inserm.fr/cgi-bin/npsa_automat.pl?page=/NPSA/npsa_blast.html>
        + ClustalW P <https://npsa.lyon.inserm.fr/cgi-bin/npsa_automat.pl?page=/NPSA/npsa_clustalw.html>
        + FASTA P <https://npsa.lyon.inserm.fr/cgi-bin/npsa_automat.pl?page=/NPSA/npsa_fasta.html>
    o Specialized
        + Annotate <https://hbvdb.lyon.inserm.fr/HBVdb/HBVdbAnnotate>
        + Genotype <https://hbvdb.lyon.inserm.fr/HBVdb/HBVdbGenotype>
        + Resistance <https://hbvdb.lyon.inserm.fr/HBVdb/HBVdbResistance>
* HBVdb
    o About <https://hbvdb.lyon.inserm.fr/HBVdb/HBVdbAbout>
    o Contact <https://hbvdb.lyon.inserm.fr/HBVdb/HBVdbContact>

o Help
+ Annotate <https://hbvdb.lyon.inserm.fr/HBVdb/HBVdbHelp?userhelp=Annotate>
+ Genotype <https://hbvdb.lyon.inserm.fr/HBVdb/HBVdbHelp?userhelp=Genotype>
+ Home <https://hbvdb.lyon.inserm.fr/HBVdb/HBVdbHelp>
+ Resistance <https://hbvdb.lyon.inserm.fr/HBVdb/HBVdbHelp?userhelp=Resistance>
o News <https://hbvdb.lyon.inserm.fr/HBVdb/HBVdbNews>
o Statistics <https://hbvdb.lyon.inserm.fr/HBVdb/HBVdbStats>
* Links
o PRABI
+ databases
# BCL2DB <https://bcl2db.lyon.inserm.fr/>
# BYKdb <https://bykdb.lyon.inserm.fr/>
# euHCVdb <https://euhcvdb.lyon.inserm.fr/>
+ centers
# all <http://www.prabi.fr/>
Gerland <https://prabi.lyon.inserm.fr/>
+ tools
# GENO3D <https://geno3d.lyon.inserm.fr/>
# NPS@ <https://npsa.lyon.inserm.fr/>
# QueBio <https://quebio.lyon.inserm.fr/>
o external
+ databases
# ENA <http://www.ebi.ac.uk/ena/>
# PDB <http://www.wwpdb.org/>
# UniProtKB <http://www.uniprot.org/>
+ centers
# EBI <http://www.ebi.ac.uk/>
# NCBI <http://www.ncbi.nlm.nih.gov/>
# RCSB <http://www.pdb.org/>
# SIB <http://www.isb-sib.ch/>

Annotate results for OM721314: text entry in EMBL format

```
ID   OM721314; SV 1; circular; genomic DNA; STD; VRL; 3182 BP.
XX
AC   OM721314;
XX
XX
DT   11-FEB-2022 (Rel. 0, Created)
DT   11-FEB-2022 (Rel. 0, Last updated, Version 0)
XX
DE   Hepatitis B Virus genotype D. Complete genome.
XX
KW   HBc; HBx; LHBs; MHBs; SHBs; Pol; HBSP; complete genome; Sensitive to drugs.
XX
```

```
OS   Hepatitis B Virus genotype D
OC   Viruses; Retro-transcribing viruses; Hepadnaviridae; Orthohepadnavirus.
XX
RN   1
RP   1-3182
RA   Unknown M;
RL   Submitted (11-FEB-2022) to FR-IBCP-PRABI-ISA.
XX
CC   The data provided in this entry have been computed thanks to
CC   the Hepatitis B Virus Database (HBVdb) annotation alogrithms.
CC   HBVdb is available at http://hbvdb.ibcp.fr.
XX
FH   Key             Location/Qualifiers
FH
FT   source          1..3182
FT                   /mol_type="genomic DNA"
FT                   /organism="Hepatitis B Virus"
FT                   /db_xref="taxon:10407"
FT                   /db_xref="HBVdb:AF121240"
FT                   /PRABI_genotype="n.a.:D:n.a."
FT   CDS             1814..2452
FT                   /PRABI_name="PreC"
FT                   /locus_tag="HBVORF02"
FT                   /note="non-functional"
FT   CDS             1901..2452
FT                   /PRABI_name="C"
FT                   /locus_tag="HBVORF12"
FT                   /codon_start="1"
FT                   /translation="MDIDPYKEFGATVELLSFLPSDFFPSVRDLLDTASALYREALESP
FT                   EHCSPHHTALRQAILCWGELMTLATWVGGNLEDPISRDLVVSYVNTNMGLKFRQLLWFH
FT                   ISCLTFGRETVIEYLVSFGVWIRTPPAYRPPNAPILSTLPETTVVRRRGRSPRRRTPSP
FT                   RRRRSQSPRRRRSQSRESQC"
FT   mat_peptide     1901..2449
FT                   /function="Core protein coding sequence"
FT                   /locus_tag="HBVORF12"
FT                   /product="Core protein"
FT                   /PRABI_name="HBc"
FT                   /PRABI_prodft=(pos:1..183, CHAIN, "Core protein")
FT   CDS             1374..1838
FT                   /PRABI_name="X"
FT                   /locus_tag="HBVORF03"
FT                   /codon_start="1"
FT                   /translation="MAARLCCQLDPARDVLCLRPVGAESRGRPFSGPLGTLSSPSPSAV
FT                   STDHGAHLSLRGLPVCAFSSAGPCALRFTSARRMETTVNAHQFLPKVLHKRTLGLSVMS
FT                   TTDLEAYFKDCLFKDWEELGEEIRLKVFVLGGCRHKLVCAPAPCNFFTSA"
FT   mat_peptide     1374..1835
FT                   /function="X protein coding sequence"
FT                   /locus_tag="HBVORF03"
FT                   /product="X protein"
FT                   /PRABI_name="HBx"
```

```
FT                   /PRABI_prodft=(pos:1..154, CHAIN, "X protein")
FT   CDS             join(2848..3182,1..835)
FT                   /PRABI_name="PreS1"
FT                   /locus_tag="HBVORF01"
FT                   /codon_start="1"
FT                   /translation="MGQNLSTSNPLGFFPDHQLDPAFRANTANPDWDFNPNKDTWPDAN
FT                   KVGAGAFGLGFTPPHGGLLGWSPQAQGILQTLPANPPPAATNRQSGRQPTPLSPPLRNT
FT                   HPQAMQWNSTTFHQTLQDPRVRGLYFPAGGSSSGTVNPVPTTVSHISSIFSRIGDPALN
FT                   MENITSGFLGPLLVLQAGFFLLTRILTIPQSLDSWWTSLNFLGGTTVCLGQNSQSPTSN
FT                   HSPTSCPPTCPGYRWMCLRRFIIFLFILLLCLIFLLVLLDYQGMLPVCPLIPGSSTTST
FT                   GPCRTCTTPAQGTSMYPSCCCTKPSDGNCTCIPIPSSWAFGKFLWEWASARFSWLSLLV
FT                   PFVQWFVGLSPTVWLSVIWMMWYWGPSLYSILSPFLPLLPIFFCLWVYI"
FT   mat_peptide     join(2848..3182,1..832)
FT                   /function="PreS1/Large Surface protein coding sequence"
FT                   /locus_tag="HBVORF01"
FT                   /product="PreS1 Surface protein"
FT                   /PRABI_name="LHBs"
FT                   /PRABI_prodft=(pos:1..389, CHAIN, "Large Surface protein")
FT   CDS             join(3172..3182,1..835)
FT                   /PRABI_name="PreS2"
FT                   /locus_tag="HBVORF11"
FT                   /codon_start="1"
FT                   /translation="MQWNSTTFHQTLQDPRVRGLYFPAGGSSSGTVNPVPTTVSHISSI
FT                   FSRIGDPALNMENITSGFLGPLLVLQAGFFLLTRILTIPQSLDSWWTSLNFLGGTTVCL
FT                   GQNSQSPTSNHSPTSCPPTCPGYRWMCLRRFIIFLFILLLCLIFLLVLLDYQGMLPVCP
FT                   LIPGSSTTSTGPCRTCTTPAQGTSMYPSCCCTKPSDGNCTCIPIPSSWAFGKFLWEWAS
FT                   ARFSWLSLLVPFVQWFVGLSPTVWLSVIWMMWYWGPSLYSILSPFLPLLPIFFCLWVYI
FT                   "
FT   mat_peptide     join(3172..3182,1..832)
FT                   /function="PreS2/Middle Surface protein coding sequence"
FT                   /locus_tag="HBVORF11"
FT                   /product="PreS2 Surface protein"
FT                   /PRABI_name="MHBs"
FT                   /PRABI_prodft=(pos:1..281, CHAIN, "Middle Surface protein")
FT   CDS             155..835
FT                   /PRABI_name="S"
FT                   /locus_tag="HBVORF21"
FT                   /codon_start="1"
FT                   /translation="MENITSGFLGPLLVLQAGFFLLTRILTIPQSLDSWWTSLNFLGGT
FT                   TVCLGQNSQSPTSNHSPTSCPPTCPGYRWMCLRRFIIFLFILLLCLIFLLVLLDYQGML
FT                   PVCPLIPGSSTTSTGPCRTCTTPAQGTSMYPSCCCTKPSDGNCTCIPIPSSWAFGKFLW
FT                   EWASARFSWLSLLVPFVQWFVGLSPTVWLSVIWMMWYWGPSLYSILSPFLPLLPIFFCL
FT                   WVYI"
FT   mat_peptide     155..832
FT                   /function="S protein coding sequence"
FT                   /locus_tag="HBVORF21"
FT                   /product="Surface protein S"
FT                   /PRABI_name="SHBs"
FT                   /PRABI_prodft=(pos:1..226, CHAIN, "Small Surface protein")
FT   CDS             join(2307..3182,1..1623)
```

```
FT                   /PRABI_name="P"
FT                   /locus_tag="HBVORF13"
FT                   /codon_start="1"
FT                   /translation="MPLSYQHFRRLLLLDDEAGPLEEELPRLADEGLNRRVAEDLNLGN
FT                   LNVSIPWTHKVGNFTGLYSSTVPVFNPHWKTPSFPNIHLHQDIIKKCEHFVGPLTVNEK
FT                   RRLQLIMPARFYPKVTKYLPLDKGIKPYYPEHLVNHYFQTRHYLHTLWKAGILYKRETT
FT                   HSASFYGSPYSWEQELQHGAESFHQQSSGILSRPPVGSSLQSKHRKSRLGLQSQQGHLA
FT                   RRQQGRSWSIRAGIHPTARRPFGVEPSGSGHTTNLASKSASCRYQSPVRKAAYPSVSTF
FT                   EKHSSSGHAVELHNLPPNSARSQSERPVFPCWWLQFRNSKPCSDYCLSHIVNLLEDWGP
FT                   CAEHGEHHIRIPRTPARVTGGVFLVDKNPHNTAESRLVVDFSQFSRGNYRVSWPKFAVP
FT                   NLQSLTNLLSSNLSWLSLDVSAAFYHLPLHPAAMPHLLVGSSGLSRYVARLSSNSRIFN
FT                   HQHGTMQNLHDSCSRNLYVSLLLLYQTFGRKLHLYSHPIILGFRKIPMGVGLSPFLLAQ
FT                   FTSAICSVVRRAFPHCLAFSYMDDVVLGAKSVQHLESIFTAVTNFLLSLGIHLNPNKTK
FT                   RWGYSLHFMGYVIGCYGSLPQDHIIQKIKECFRKLPVNRPIDWKVCQRIVGLLGFAAPF
FT                   TQCGYPALMPLYACIQSKLAFTFSPTYKAFLCKQYLNLYPVARQRPGLCQVFADATPTG
FT                   WGLVMGHQRMRGTFSAPLPIHTAELLAACFARSRSGANILGTDNSVVLSRKYTSFPWLL
FT                   GCAANWILRGTSFVYVPSALNPADDPSRGRLGLSRPLLRLPFRPTTGRTSLYADSPSVP
FT                   SHLPDRVHFASPLHVAWRPP"
FT   mat_peptide     join(2307..3182,1..1620)
FT                   /function="DNA-polymerase/Reverse Transcriptase coding
FT                   sequence"
FT                   /locus_tag="HBVORF13"
FT                   /product="Polymerase/Reverse Transcriptase"
FT                   /PRABI_name="Pol"
FT                   /PRABI_prodft=(pos:1..832, CHAIN, "Polymerase/Reverse
FT                   transcriptase")
FT                   /PRABI_prodft=(pos:1..180, DOMAIN, "Terminal Protein (TP)/
FT                   Primase domain")
FT                   /PRABI_prodft=(pos:181..335, DOMAIN, "Spacer")
FT                   /PRABI_prodft=(pos:336..679, DOMAIN, "Reverse Transcriptase
FT                   (RT) domain")
FT                   /PRABI_prodft=(pos:418..418, ACT_SITE, "RT catalytic Asp")
FT                   /PRABI_prodft=(pos:540..540, ACT_SITE, "RT catalytic Asp")
FT                   /PRABI_prodft=(pos:541..541, ACT_SITE, "RT catalytic Asp")
FT                   /PRABI_prodft=(pos:680..832, DOMAIN, "Ribonuclease H
FT                   (RNaseH) domain")
FT                   /PRABI_prodft=(pos:689..689, ACT_SITE, "RNaseH catalytic
FT                   Asp")
FT                   /PRABI_prodft=(pos:718..718, ACT_SITE, "RNaseH catalytic
FT                   Glu")
FT                   /PRABI_prodft=(pos:737..737, ACT_SITE, "RNaseH catalytic
FT                   Asp")
FT   CDS             join(2307..2447,489..683)
FT                   /PRABI_name="SP"
FT                   /locus_tag="HBVORF04"
FT                   /codon_start="1"
FT                   /translation="MPLSYQHFRRLLLLDDEAGPLEEELPRLADEGLNRRVAEDLNLGN
FT                   LNDLQPPARDHAEPARLLLKEPLCIPPAAVPNLRTEIAPVFPSHHPGLSENSYGSGPQP
FT                   VSPGSVY"
FT   mat_peptide     join(2307..2447,489..680)
```

```
FT                   /function="HBSP coding sequence"
FT                   /locus_tag="HBVORF04"
FT                   /product="Hepatitis B Spliced Protein"
FT                   /PRABI_name="HBSP"
FT                   /PRABI_prodft=(pos:1..111, CHAIN, "HBV Spliced Protein")
XX
SQ   Sequence 3182 BP; 728 A; 860 C; 691 G; 903 T; 0 other;
     ctccacaacc ttccaccaaa ctctgcaaga tcccagagtg agaggcctgt atttccctgc        60
     tggtggctcc agttcaggaa cagtaaaccc tgttccgact actgtctctc acatatcgtc       120
     aatcttctcg aggattgggg accctgcgct gaacatggag aacatcacat caggattcct       180
     aggacccctg ctcgtgttac aggcggggtt tttcttgttg acaagaatcc tcacaatacc       240
     gcagagtcta gactcgtggt ggacttctct caattttcta gggggaacta ccgtgtgtct       300
     tggccaaaat tcgcagtccc caacctccaa tcactcacca acctcctgtc ctccaacttg       360
     tcctggttat cgctggatgt gtctgcggcg ttttatcatc ttcctcttca tcctgctgct       420
     atgcctcatc ttcttgttgg ttcttctgga ctatcaaggt atgttgcccg tttgtcctct       480
     aattccagga tcttcaacca ccagcacggg accatgcaga acctgcacga ctcctgctca       540
     aggaacctct atgtatccct cctgctgctg taccaaacct tcggacggaa attgcacctg       600
     tattcccatc ccatcatcct gggctttcgg aaaattccta tgggagtggg cctcagcccg       660
     tttctcctgg ctcagtttac tagtgccatt tgttcagtgg ttcgtagggc tttcccccac       720
     tgtttggctt tcagttatat ggatgatgtg gtattggggg ccaagtctgt acagcatctt       780
     gagtccattt ttaccgctgt taccaatttt cttttgtctc tgggtataca tttaaaccct       840
     aacaaaacaa aaagatgggg ttactcttta catttcatgg gctatgtcat tggatgttat       900
     gggtcattgc cacaagatca catcatacag aaaatcaaag aatgttttag aaaacttcct       960
     gttaacaggc ctattgattg gaaagtctgt caacgtattg tgggtctttt gggttttgct      1020
     gccccttta cacaatgtgg ttatcctgct ttaatgccct tgtatgcatg tattcaatct      1080
     aagctggctt tcactttctc gccaacttac aaggcctttc tgtgtaaaca atacctgaac      1140
     ctttaccccg ttgcccggca acggccaggt ctgtgccaag tgtttgctga cgcaaccccc      1200
     actggctggg gcttggtcat gggccatcag cgcatgcgtg gaacctttc ggctcctctg       1260
     ccgatccata ctgcggaact cctagccgct tgttttgctc gcagcaggtc tggagcaaac      1320
     attctcggga cggataactc tgttgttctc tcccgcaaat atacgtcgtt tccatggctg      1380
     ctaggctgtg ctgccaactg gatcctgcgc gggacgtcct ttgtttacgt cccgtcggcg      1440
     ctgaatcccg cggacgaccc ttctcggggc cgcttgggac tctctcgtcc ccttctccgt      1500
     ctgccgtttc gaccgaccac ggggcgcacc tctctttacg cggactcccc gtctgtgcct      1560
     tctcatctgc cggaccgtgt gcacttcgct tcacctctgc acgtcgcatg gagaccaccg      1620
     tgaacgccca ccaattcttg cccaaggtct tacataagag gactcttgga ctctctgtga      1680
     tgtcaacgac cgaccttgag gcatacttca aagactgttt gtttaaagac tgggaggagt      1740
     tgggggagga gattagatta aaggtctttg tattaggagg ctgtaggcat aaattggtct      1800
     gcgcaccagc accatgcaac tttttcacct ctgcctaatc atctcttgtt catgtcctac      1860
     tgttcaagcc tccaagctgt gccttgggtg gctttagggc atggacattg atccttataa      1920
     agaatttgga gctactgtgg agttactctc atttttgcct tctgacttct ttccttcagt      1980
     acgagatctt ctagataccg cctcagctct atatcgggaa gccttagagt ctcctgagca      2040
     ttgttcacct caccatactg cactcaggca agcaattctt tgctgggggg aactaatgac      2100
     tttagccacc tgggtgggtg gtaatttgga agatccaata tccagggacc tagtagtcag      2160
     ttatgttaac actaatatgg gcctaaagtt caggcaacta ttgtggtttc acatttcttg      2220
     tctcactttt ggaagagaaa cggtcataga gtatttggtg tctttcggag tgtggattcg      2280
     cactcctcca gcttatagac caccaaatgc ccctatctta tcaacacttc cggagactac      2340
     tgttgttaga cgacgaggca ggtcccctag aagaagaact ccctcgcctc gcagacgaag      2400
     gtctcaatcg ccgcgtcgca gaagatctca atctcgggaa tctcaatgtt agtattcctt      2460
     ggactcataa ggtgggaaac tttacggggc tttattcttc tactgttcct gtctttaacc      2520
     ctcattggaa aacaccctct ttcccaata tacacttaca ccaagacatt atcaaaaaat       2580
```

```
     gtgaacattt tgtaggccca ctcacagtca atgagaaaag aagactgcaa ttgattatgc      2640
     ctgctaggtt ctatccaaag gttaccaaat atttgccatt ggataagggt attaaacctt      2700
     attatccaga acatctagtt aatcattact tccaaaccag acattattta cacactctat      2760
     ggaaggcggg tatattatat aagagagaaa caacacatag cgcctcattt tatgggtcac      2820
     catattcttg ggaacaagag ctacagcatg gggcagaatc tttccaccag caatcctctg      2880
     ggattctttc ccgaccacca gttggatcca gccttcagag caaacaccgc aaatccagat      2940
     tgggacttca atcccaacaa ggacacctgg ccagacgcca acaaggtagg agctggagca      3000
     ttcgggctgg gattcacccc accgcacgga ggccttttgg ggtggagccc tcaggctcag      3060
     ggcatactac aaaccttgcc agcaaatccg cctcctgccg ctaccaatcg ccagtcagga      3120
     aggcagccta cccctctgtc tccacctttg agaaacactc atcctcaggc catgcagtgg      3180
     aa                                                                     3182
//
```

© 1998-2022 Centre de Recherche en Cancerologie de Lyon logo <http://www.crcl.fr/> Pole Rhone-Alpes de BioInformatique logo <http://prabi.ibcp.fr/> Centre National de la Recherche Scientifique logo <http://www.cnrs.fr/> Institut national de la sante et de la recherche medicale logo <http://www.inserm.fr/> Universite Claude Bernard Lyon 1 logo <http://www.univ-lyon1.fr/> Legal notice <https://hbvdb.lyon.inserm.fr/HBVdb/HBVdbAbout#legalnotice>

HBVdb

* Home <https://hbvdb.lyon.inserm.fr/HBVdb/HBVdbIndex>
* HBV
  o Genome <https://hbvdb.lyon.inserm.fr/HBVdb/HBVdbGenome>
  o Nomenclature <https://hbvdb.lyon.inserm.fr/HBVdb/HBVdbNomenclature>
  o Proteins
    + Core <https://hbvdb.lyon.inserm.fr/HBVdb/HBVdbProteins?protein=Core>
    + HBx <https://hbvdb.lyon.inserm.fr/HBVdb/HBVdbProteins?protein=HBx>
    + Surface <https://hbvdb.lyon.inserm.fr/HBVdb/HBVdbProteins?protein=Surface>
    + Polymerase <https://hbvdb.lyon.inserm.fr/HBVdb/HBVdbProteins?protein=Polymerase>
* Query
  o Dataset
    + Nucleotide <https://hbvdb.lyon.inserm.fr/HBVdb/HBVdbDataset?seqtype=0>
    + Protein <https://hbvdb.lyon.inserm.fr/HBVdb/HBVdbDataset?seqtype=2>
* Analysis
  o Generic N
    + Blast N

<https://npsa.lyon.inserm.fr/cgi-bin/npsa_automat.pl?page=/NPSA/npsa_blastan.html>
    + ClustalW N

<https://npsa.lyon.inserm.fr/cgi-bin/npsa_automat.pl?page=/NPSA/npsa_clustalwan.html>
    + FASTA N

<https://npsa.lyon.inserm.fr/cgi-bin/npsa_automat.pl?page=/NPSA/npsa_fastaan.html>
  o Generic P
    + Blast P

<https://npsa.lyon.inserm.fr/cgi-bin/npsa_automat.pl?page=/NPSA/npsa_blast.html>
    + ClustalW P

<https://npsa.lyon.inserm.fr/cgi-bin/npsa_automat.pl?page=/NPSA/npsa_clustalw.html>
    + FASTA P

<https://npsa.lyon.inserm.fr/cgi-bin/npsa_automat.pl?page=/NPSA/npsa_fasta.html>
  o Specialized
    + Annotate <https://hbvdb.lyon.inserm.fr/HBVdb/HBVdbAnnotate>
    + Genotype <https://hbvdb.lyon.inserm.fr/HBVdb/HBVdbGenotype>
    + Resistance <https://hbvdb.lyon.inserm.fr/HBVdb/HBVdbResistance>
* HBVdb
  o About <https://hbvdb.lyon.inserm.fr/HBVdb/HBVdbAbout>
  o Contact <https://hbvdb.lyon.inserm.fr/HBVdb/HBVdbContact>

```
      o Help
          + Annotate
            <https://hbvdb.lyon.inserm.fr/HBVdb/HBVdbHelp?userhelp=Annotate>
          + Genotype
            <https://hbvdb.lyon.inserm.fr/HBVdb/HBVdbHelp?userhelp=Genotype>
          + Home <https://hbvdb.lyon.inserm.fr/HBVdb/HBVdbHelp>
          + Resistance
            <https://hbvdb.lyon.inserm.fr/HBVdb/HBVdbHelp?userhelp=Resistance>
      o News <https://hbvdb.lyon.inserm.fr/HBVdb/HBVdbNews>
      o Statistics <https://hbvdb.lyon.inserm.fr/HBVdb/HBVdbStats>
  * Links
      o PRABI
          + databases
              # BCL2DB <https://bcl2db.lyon.inserm.fr/>
              # BYKdb <https://bykdb.lyon.inserm.fr/>
              # euHCVdb <https://euhcvdb.lyon.inserm.fr/>
          + centers
              # all <http://www.prabi.fr/>
              # Gerland <https://prabi.lyon.inserm.fr/>
          + tools
              # GENO3D <https://geno3d.lyon.inserm.fr/>
              # NPS@ <https://npsa.lyon.inserm.fr/>
              # QueBio <https://quebio.lyon.inserm.fr/>
      o external
          + databases
              # ENA <http://www.ebi.ac.uk/ena/>
              # PDB <http://www.wwpdb.org/>
              # UniProtKB <http://www.uniprot.org/>
          + centers
              # EBI <http://www.ebi.ac.uk/>
              # NCBI <http://www.ncbi.nlm.nih.gov/>
              # RCSB <http://www.pdb.org/>
              # SIB <http://www.isb-sib.ch/>
```

Annotate results for OM721315: text entry in EMBL format

```
ID   OM721315; SV 1; circular; genomic DNA; STD; VRL; 3182 BP.
XX
AC   OM721315;
XX
XX
DT   11-FEB-2022 (Rel. 0, Created)
DT   11-FEB-2022 (Rel. 0, Last updated, Version 0)
XX
DE   Hepatitis B Virus genotype D. Complete genome.
XX
KW   HBe; HBc; HBx; LHBs; MHBs; SHBs; Pol; HBSP; complete genome; Sensitive to
drugs.
```

```
XX
OS   Hepatitis B Virus genotype D
OC   Viruses; Retro-transcribing viruses; Hepadnaviridae; Orthohepadnavirus.
XX
RN   1
RP   1-3182
RA   Unknown M;
RL   Submitted (11-FEB-2022) to FR-IBCP-PRABI-ISA.
XX
CC   The data provided in this entry have been computed thanks to
CC   the Hepatitis B Virus Database (HBVdb) annotation alogrithms.
CC   HBVdb is available at http://hbvdb.ibcp.fr.
XX
FH   Key             Location/Qualifiers
FH
FT   source          1..3182
FT                   /mol_type="genomic DNA"
FT                   /organism="Hepatitis B Virus"
FT                   /db_xref="taxon:10407"
FT                   /db_xref="HBVdb:AF121240"
FT                   /PRABI_genotype="n.a.:D:n.a."
FT   CDS             1814..2452
FT                   /PRABI_name="PreC"
FT                   /locus_tag="HBVORF02"
FT                   /codon_start="1"
FT                   /translation="MQLFHLCLIISCSCPTVQASKLCLGWLWGMDIDPYKEFGATVELL
FT                   SFLPSDFFPSVRDLLDTASALYREALESPEHCSPHHTALRQAILCWGELMTLATWVGGN
FT                   LEDPISRDLVVSYVNTNMGLKFRQLLWFHISCLTFGRETVIEYLVSFGVWIRTPPAYRP
FT                   PNAPILSTLPETTVVRRRGRSPRRRTPSPRRRRSQSPRRRRSQSRESQC"
FT   mat_peptide     1871..2275
FT                   /function="HBe/External core antigen coding sequence"
FT                   /locus_tag="HBVORF02"
FT                   /product="External core antigen"
FT                   /PRABI_name="HBe"
FT                   /PRABI_prodft=(pos:20..154, CHAIN, "HBe antigen")
FT   CDS             1901..2452
FT                   /PRABI_name="C"
FT                   /locus_tag="HBVORF12"
FT                   /codon_start="1"
FT                   /translation="MDIDPYKEFGATVELLSFLPSDFFPSVRDLLDTASALYREALESP
FT                   EHCSPHHTALRQAILCWGELMTLATWVGGNLEDPISRDLVVSYVNTNMGLKFRQLLWFH
FT                   ISCLTFGRETVIEYLVSFGVWIRTPPAYRPPNAPILSTLPETTVVRRRGRSPRRRTPSP
FT                   RRRRSQSPRRRRSQSRESQC"
FT   mat_peptide     1901..2449
FT                   /function="Core protein coding sequence"
FT                   /locus_tag="HBVORF12"
FT                   /product="Core protein"
FT                   /PRABI_name="HBc"
FT                   /PRABI_prodft=(pos:1..183, CHAIN, "Core protein")
FT   CDS             1374..1838
```

```
FT                   /PRABI_name="X"
FT                   /locus_tag="HBVORF03"
FT                   /codon_start="1"
FT                   /translation="MAARLCCQLDPARDVLCLRPVGAESRGRPFSGPLGSLSSPSPSAV
FT                   STDHGAHLSLRGLPVCAFSSAGPCALRFTSARRMETTVNAHQFLPKVLHKRTLGLSVMS
FT                   TTDLEAYFKDCLFKDWEELGEELRLKVFVLGGCRHKLVCAPAPCNFFTSA"
FT   mat_peptide     1374..1835
FT                   /function="X protein coding sequence"
FT                   /locus_tag="HBVORF03"
FT                   /product="X protein"
FT                   /PRABI_name="HBx"
FT                   /PRABI_prodft=(pos:1..154, CHAIN, "X protein")
FT   CDS             join(2848..3182,1..835)
FT                   /PRABI_name="PreS1"
FT                   /locus_tag="HBVORF01"
FT                   /codon_start="1"
FT                   /translation="MGQNLSTSNPLGFFPDHQLDPAFRANTANPDWDFNPNKDTWPDAN
FT                   KVGAGAFGLGFTPPHGGLLGWSPQAQGILQTLPTNPPPASTNRQSGRQPTPLSPPLRNT
FT                   HPQAMQWNSTTFHQTLQDPRVRGLYFPAGGSSSGTVNPVPTTVSHISSIFSRIGDPALN
FT                   MENITSGFLGPLLVLQAGFFLLTRILTIPQSLDSWWTSLNFLGGTTVCLGQNSQSPTSN
FT                   HSPTSCPPTCPGYRWMCLRRFIIFLFILLLCLIFLLVLLDYQGMLPVCPLIPGSSTTST
FT                   GPCRTCTTPAQGTSMYPSCCCTKPSDGNCTCIPIPSSWAFGKFLWEWASARFSWLSSLV
FT                   PFVQWFVGLSPTVWLSVIWMMWYWGPSLYSILSPFLPLLPIFFCLWVYI"
FT   mat_peptide     join(2848..3182,1..832)
FT                   /function="PreS1/Large Surface protein coding sequence"
FT                   /locus_tag="HBVORF01"
FT                   /product="PreS1 Surface protein"
FT                   /PRABI_name="LHBs"
FT                   /PRABI_prodft=(pos:1..389, CHAIN, "Large Surface protein")
FT   CDS             join(3172..3182,1..835)
FT                   /PRABI_name="PreS2"
FT                   /locus_tag="HBVORF11"
FT                   /codon_start="1"
FT                   /translation="MQWNSTTFHQTLQDPRVRGLYFPAGGSSSGTVNPVPTTVSHISSI
FT                   FSRIGDPALNMENITSGFLGPLLVLQAGFFLLTRILTIPQSLDSWWTSLNFLGGTTVCL
FT                   GQNSQSPTSNHSPTSCPPTCPGYRWMCLRRFIIFLFILLLCLIFLLVLLDYQGMLPVCP
FT                   LIPGSSTTSTGPCRTCTTPAQGTSMYPSCCCTKPSDGNCTCIPIPSSWAFGKFLWEWAS
FT                   ARFSWLSSLVPFVQWFVGLSPTVWLSVIWMMWYWGPSLYSILSPFLPLLPIFFCLWVYI
FT                   "
FT   mat_peptide     join(3172..3182,1..832)
FT                   /function="PreS2/Middle Surface protein coding sequence"
FT                   /locus_tag="HBVORF11"
FT                   /product="PreS2 Surface protein"
FT                   /PRABI_name="MHBs"
FT                   /PRABI_prodft=(pos:1..281, CHAIN, "Middle Surface protein")
FT   CDS             155..835
FT                   /PRABI_name="S"
FT                   /locus_tag="HBVORF21"
FT                   /codon_start="1"
FT                   /translation="MENITSGFLGPLLVLQAGFFLLTRILTIPQSLDSWWTSLNFLGGT
```

```
FT                   TVCLGQNSQSPTSNHSPTSCPPTCPGYRWMCLRRFIIFLFILLLCLIFLLVLLDYQGML
FT                   PVCPLIPGSSTTSTGPCRTCTTPAQGTSMYPSCCCTKPSDGNCTCIPIPSSWAFGKFLW
FT                   EWASARFSWLSSLVPFVQWFVGLSPTVWLSVIWMMWYWGPSLYSILSPFLPLLPIFFCL
FT                   WVYI"
FT   mat_peptide     155..832
FT                   /function="S protein coding sequence"
FT                   /locus_tag="HBVORF21"
FT                   /product="Surface protein S"
FT                   /PRABI_name="SHBs"
FT                   /PRABI_prodft=(pos:1..226, CHAIN, "Small Surface protein")
FT   CDS             join(2307..3182,1..1623)
FT                   /PRABI_name="P"
FT                   /locus_tag="HBVORF13"
FT                   /codon_start="1"
FT                   /translation="MPLSYQHFRRLLLLDDEAGPLEEELPRLADEGLNRRVAEDLNLGN
FT                   LNVSIPWTHKVGNFTGLYSSTVPVFNPHWKTPSFPNIHLHQNIIKKCEQFVGPLTVNEK
FT                   RRLQLIMPARFYPNVTKYLPLDKGIKPYYPEHLVNHYFQTRHYLHTLWKAGILYKRETT
FT                   HSASFCGSPYSWEQELQHGAESFHQQSSGILSRPPVGSSLQSKHCKSRLGLQSQQGHLA
FT                   RRQQGRSWSIRAGIHPTARRPFGVEPSGSGHTTNLANKSASCLYQSPVRKAAYPSVSTF
FT                   EKHSSSGHAVELHNLPPNSARSQSERPVFPCWWLQFRNSKPCSDYCLSHIVNLLEDWGP
FT                   CAEHGEHHIRIPRTPARVTGGVFLVDKNPHNTAESRLVVDFSQFSRGNYRVSWPKFAVP
FT                   NLQSLTNLLSSNLSWLSLDVSAAFYHLPLHPAAMPHLLVGSSGLSRYVARLSSNSRIVN
FT                   HQHGPMQNLHDSCSRNLYVSLLLLYQTFGRKLHLYSHPIILGFRKIPMGVGLSPFLLAQ
FT                   FTSAICSVVRRAFPHCLAFSYMDDVVLGAKSVQHLESLFTAVTNFLLSLGIHLNPNKTK
FT                   RWGYSLHFMGYVIGCYGSLPQDHIIQKIKECFRKLPINRPIDWKVCQRIVGLLGFAAPF
FT                   TQCGYPALMPLYACIQSKQAFTFSPTYKAFLCKQYLNLYPVARQRPGLCQVFADATPTG
FT                   WGLVMGHQRMRGTFRAPLPIHTAELLAACFARSRSGANILGTDNSVVLSRKYTSFPWLL
FT                   GCAANWILRGTSFVYVPSALNPADDPSRGRLGLSRPLLRLPFRPTTGRTSLYADSPSVP
FT                   SHLPDRVHFASPLHVAWRPP"
FT   mat_peptide     join(2307..3182,1..1620)
FT                   /function="DNA-polymerase/Reverse Transcriptase coding
FT                   sequence"
FT                   /locus_tag="HBVORF13"
FT                   /product="Polymerase/Reverse Transcriptase"
FT                   /PRABI_name="Pol"
FT                   /PRABI_prodft=(pos:1..832, CHAIN, "Polymerase/Reverse
FT                   transcriptase")
FT                   /PRABI_prodft=(pos:1..180, DOMAIN, "Terminal Protein (TP)/
FT                   Primase domain")
FT                   /PRABI_prodft=(pos:181..335, DOMAIN, "Spacer")
FT                   /PRABI_prodft=(pos:336..679, DOMAIN, "Reverse Transcriptase
FT                   (RT) domain")
FT                   /PRABI_prodft=(pos:418..418, ACT_SITE, "RT catalytic Asp")
FT                   /PRABI_prodft=(pos:540..540, ACT_SITE, "RT catalytic Asp")
FT                   /PRABI_prodft=(pos:541..541, ACT_SITE, "RT catalytic Asp")
FT                   /PRABI_prodft=(pos:680..832, DOMAIN, "Ribonuclease H
FT                   (RNaseH) domain")
FT                   /PRABI_prodft=(pos:689..689, ACT_SITE, "RNaseH catalytic
FT                   Asp")
FT                   /PRABI_prodft=(pos:718..718, ACT_SITE, "RNaseH catalytic
```

```
FT                   Glu")
FT                   /PRABI_prodft=(pos:737..737, ACT_SITE, "RNaseH catalytic
FT                   Asp")
FT   CDS             join(2307..2447,489..683)
FT                   /PRABI_name="SP"
FT                   /locus_tag="HBVORF04"
FT                   /codon_start="1"
FT                   /translation="MPLSYQHFRRLLLLDDEAGPLEEELPRLADEGLNRRVAEDLNLGN
FT                   LNDRQPPARAHAEPARLLLKEPLCIPPVAVPNLRTEIAPVFPSHHPGLSENSYGSGPQP
FT                   VSPGSVH"
FT   mat_peptide     join(2307..2447,489..680)
FT                   /function="HBSP coding sequence"
FT                   /locus_tag="HBVORF04"
FT                   /product="Hepatitis B Spliced Protein"
FT                   /PRABI_name="HBSP"
FT                   /PRABI_prodft=(pos:1..111, CHAIN, "HBV Spliced Protein")
XX
SQ   Sequence 3182 BP; 723 A; 863 C; 690 G; 906 T; 0 other;
     ctccacaacc ttccaccaaa ctctgcaaga tcccagagtg agaggcctgt atttccctgc        60
     tggtggctcc agttcaggaa cagtaaaccc tgttccgact actgtctctc acatatcgtc       120
     aatcttctcg aggattgggg accctgcgct gaacatggag aacatcacat caggattcct       180
     aggacccctg ctcgtgttac aggcggggtt tttcttgttg acaagaatcc tcacaatacc       240
     gcagagtcta gactcgtggt ggacttctct caattttcta gggggaacta ccgtgtgtct       300
     tggccaaaat tcgcagtccc caacctccaa tcactcacca acctcctgtc ctccaacttg       360
     tcctggttat cgctggatgt gtctgcggcg ttttatcatc ttcctcttca tcctgctgct       420
     atgcctcatc ttcttgttgg ttcttctgga ctatcaaggt atgttgcccg tttgtcctct       480
     aattccagga tcgtcaacca ccagcacggg cccatgcaga acctgcacga ctcctgctca       540
     aggaacctct atgtatccct cctgttgctg taccaaacct tcggacggaa attgcacctg       600
     tattcccatc ccatcatcct gggctttcgg aaaattccta tgggagtggg cctcagcccg       660
     tttctcctgg ctcagttcac tagtgccatt tgttcagtgg ttcgtagggc tttcccccac       720
     tgtttggctt tcagttatat ggatgatgtg gtattggggg ccaagtctgt acagcatctt       780
     gagtcccttt ttaccgctgt taccaatttt cttttgtctt tgggtataca tttaaaccct       840
     aacaaaacaa aaagatgggg gtactcttta catttcatgg gctatgtcat tggatgttat       900
     ggttcattgc cacaagatca catcatacag aaaatcaaag aatgttttag aaaacttcct       960
     attaacaggc ctattgattg gaaagtctgt caacgtattg tgggtctttt gggttttgct      1020
     gccccttta cacagtgcgg ttatcctgct ttaatgcctt tatatgcctg tattcaatct      1080
     aagcaggctt tcactttctc gccaacttac aaggcctttc tgtgtaaaca atacctgaac      1140
     ctttaccccg ttgcccggca acggccaggt ctgtgccaag tgtttgctga cgcaaccccc      1200
     actggctggg gcttggtcat gggccatcag cgcatgcgtg gaacctttcg ggctcctctg      1260
     ccgatccata ctgcggaact cctagccgct tgctttgctc gcagccggtc tggagcaaac      1320
     attctcggga cggataactc tgttgttctc tcccgcaaat atacatcgtt tccatggctg      1380
     ctaggctgtg ctgccaactg gatcctgcgc gggacgtcct ttgtttacgt cccgtcggcg      1440
     ctgaatcccg cggacgaccc ttctcggggc cgcttgggtc tctctcgtcc ccttctccgt      1500
     ctgccgtttc gaccgaccac ggggcgcacc tctctttacg cggactcccc gtctgtgcct      1560
     tctcatctgc cggaccgtgt gcacttcgct tcacctctgc acgtcgcatg gagaccaccg      1620
     tgaacgccca ccaattcttg cccaaggtct tacataagag gactcttgga ctctctgtca      1680
     tgtcaacgac cgaccttgag gcatacttca aagactgttt gtttaaggac tgggaggagt      1740
     tgggggagga gcttagatta aaggtctttg tattaggagg ctgtaggcat aaattggtct      1800
     gcgcaccagc accatgcaac tttttcacct ctgcctaatc atctcttgtt catgtcctac      1860
     tgttcaagcc tccaagctgt gccttgggtg gctttggggc atggacattg atccttataa      1920
```

```
     agaatttgga gctactgtgg agttactctc gtttttgcct tctgacttct ttccttcagt      1980
     acgagatctt ctagataccg cctcagctct gtatcgggaa gccttagagt ctcctgagca      2040
     ttgttcacct caccatactg cactcaggca agcaattctt tgctgggggg aactaatgac      2100
     tttagccacc tgggtgggtg gtaatttgga agatccaata tccagagacc tagtagtcag      2160
     ttatgttaac actaatatgg gcctaaagtt caggcaacta ttgtggtttc acatttcttg      2220
     tctcactttt ggaagagaaa cggtcataga gtatttggtg tctttcggag tgtggattcg      2280
     cactcctcca gcttatagac caccaaatgc ccctatctta tcaacacttc cggagactac      2340
     tgttgttaga cgacgaggca ggtcccctag aagaagaact ccctcgcctc gcagacgaag      2400
     gtctcaatcg ccgcgtcgca gaagatctca atctcgggaa tctcaatgtt agtattcctt      2460
     ggactcataa ggtgggaaac tttacggggc tttactcttc tactgttcct gtctttaacc      2520
     ctcattggaa aacaccttct tttcctaata tacatttaca ccaaaacatt atcaaaaaat      2580
     gtgaacaatt tgtaggccca ctcacagtca atgagaaaag aagactgcag ttgattatgc      2640
     ctgctaggtt ttatccaaat gttaccaaat atttgccatt agataagggc attaaacctt      2700
     attatccaga acatctagtt aatcattact tccaaaccag acattattta cacactctat      2760
     ggaaggcggg tatattatat aagagagaaa ctacacatag cgcctcattt tgtgggtcac      2820
     catattcttg ggaacaagag ctacagcatg gggcagaatc tttccaccag caatcctctg      2880
     ggattctttc ccgaccacca gttggatcca gccttcagag caaacactgc aaatccagat      2940
     tgggacttca atcccaacaa ggacacctgg ccagacgcca acaaggtagg agctggagca      3000
     ttcgggctgg gattcacccc accgcacgga ggccttttgg ggtggagccc tcaggctcag      3060
     ggcatactac aaaccttgcc aacaaatccg cctcctgcct ctaccaatcg ccagtcagga      3120
     aggcagccta cccctctgtc tccacctttg agaaacactc atcctcaggc catgcagtgg      3180
     aa                                                                     3182
//
```

© 1998-2022 Centre de Recherche en Cancerologie de Lyon logo <http://www.crcl.fr/> Pole Rhone-Alpes de BioInformatique logo <http://prabi.ibcp.fr/> Centre National de la Recherche Scientifique logo <http://www.cnrs.fr/> Institut national de la sante et de la recherche medicale logo <http://www.inserm.fr/> Universite Claude Bernard Lyon 1 logo <http://www.univ-lyon1.fr/> Legal notice <https://hbvdb.lyon.inserm.fr/HBVdb/HBVdbAbout#legalnotice>

HBVdb

* Home <https://hbvdb.lyon.inserm.fr/HBVdb/HBVdbIndex>
* HBV
  o Genome <https://hbvdb.lyon.inserm.fr/HBVdb/HBVdbGenome>
  o Nomenclature <https://hbvdb.lyon.inserm.fr/HBVdb/HBVdbNomenclature>
  o Proteins
    + Core <https://hbvdb.lyon.inserm.fr/HBVdb/HBVdbProteins?protein=Core>
    + HBx <https://hbvdb.lyon.inserm.fr/HBVdb/HBVdbProteins?protein=HBx>
    + Surface <https://hbvdb.lyon.inserm.fr/HBVdb/HBVdbProteins?protein=Surface>
    + Polymerase <https://hbvdb.lyon.inserm.fr/HBVdb/HBVdbProteins?protein=Polymerase>
* Query
  o Dataset
    + Nucleotide <https://hbvdb.lyon.inserm.fr/HBVdb/HBVdbDataset?seqtype=0>
    + Protein <https://hbvdb.lyon.inserm.fr/HBVdb/HBVdbDataset?seqtype=2>
* Analysis
  o Generic N
    + Blast N <https://npsa.lyon.inserm.fr/cgi-bin/npsa_automat.pl?page=/NPSA/npsa_blastan.html>
    + ClustalW N <https://npsa.lyon.inserm.fr/cgi-bin/npsa_automat.pl?page=/NPSA/npsa_clustalwan.html>
    + FASTA N <https://npsa.lyon.inserm.fr/cgi-bin/npsa_automat.pl?page=/NPSA/npsa_fastaan.html>
  o Generic P
    + Blast P <https://npsa.lyon.inserm.fr/cgi-bin/npsa_automat.pl?page=/NPSA/npsa_blast.html>
    + ClustalW P <https://npsa.lyon.inserm.fr/cgi-bin/npsa_automat.pl?page=/NPSA/npsa_clustalw.html>
    + FASTA P <https://npsa.lyon.inserm.fr/cgi-bin/npsa_automat.pl?page=/NPSA/npsa_fasta.html>
  o Specialized
    + Annotate <https://hbvdb.lyon.inserm.fr/HBVdb/HBVdbAnnotate>
    + Genotype <https://hbvdb.lyon.inserm.fr/HBVdb/HBVdbGenotype>
    + Resistance <https://hbvdb.lyon.inserm.fr/HBVdb/HBVdbResistance>
* HBVdb
  o About <https://hbvdb.lyon.inserm.fr/HBVdb/HBVdbAbout>
  o Contact <https://hbvdb.lyon.inserm.fr/HBVdb/HBVdbContact>

```
      o Help
          + Annotate
            <https://hbvdb.lyon.inserm.fr/HBVdb/HBVdbHelp?userhelp=Annotate>
          + Genotype
            <https://hbvdb.lyon.inserm.fr/HBVdb/HBVdbHelp?userhelp=Genotype>
          + Home <https://hbvdb.lyon.inserm.fr/HBVdb/HBVdbHelp>
          + Resistance
            <https://hbvdb.lyon.inserm.fr/HBVdb/HBVdbHelp?userhelp=Resistance>
      o News <https://hbvdb.lyon.inserm.fr/HBVdb/HBVdbNews>
      o Statistics <https://hbvdb.lyon.inserm.fr/HBVdb/HBVdbStats>
  * Links
      o PRABI
          + databases
              # BCL2DB <https://bcl2db.lyon.inserm.fr/>
              # BYKdb <https://bykdb.lyon.inserm.fr/>
              # euHCVdb <https://euhcvdb.lyon.inserm.fr/>
          + centers
              # all <http://www.prabi.fr/>
              # Gerland <https://prabi.lyon.inserm.fr/>
          + tools
              # GENO3D <https://geno3d.lyon.inserm.fr/>
              # NPS@ <https://npsa.lyon.inserm.fr/>
              # QueBio <https://quebio.lyon.inserm.fr/>
      o external
          + databases
              # ENA <http://www.ebi.ac.uk/ena/>
              # PDB <http://www.wwpdb.org/>
              # UniProtKB <http://www.uniprot.org/>
          + centers
              # EBI <http://www.ebi.ac.uk/>
              # NCBI <http://www.ncbi.nlm.nih.gov/>
              # RCSB <http://www.pdb.org/>
              # SIB <http://www.isb-sib.ch/>
```

Annotate results for OM721316: text entry in EMBL format

```
ID   OM721316; SV 1; circular; genomic DNA; STD; VRL; 3182 BP.
XX
AC   OM721316;
XX
XX
DT   11-FEB-2022 (Rel. 0, Created)
DT   11-FEB-2022 (Rel. 0, Last updated, Version 0)
XX
DE   Hepatitis B Virus genotype D. Complete genome.
XX
KW   HBc; HBx; LHBs; MHBs; SHBs; Pol; HBSP; complete genome; Sensitive to drugs.
XX
```

```
OS   Hepatitis B Virus genotype D
OC   Viruses; Retro-transcribing viruses; Hepadnaviridae; Orthohepadnavirus.
XX
RN   1
RP   1-3182
RA   Unknown M;
RL   Submitted (11-FEB-2022) to FR-IBCP-PRABI-ISA.
XX
CC   The data provided in this entry have been computed thanks to
CC   the Hepatitis B Virus Database (HBVdb) annotation alogrithms.
CC   HBVdb is available at http://hbvdb.ibcp.fr.
XX
FH   Key             Location/Qualifiers
FH
FT   source          1..3182
FT                   /mol_type="genomic DNA"
FT                   /organism="Hepatitis B Virus"
FT                   /db_xref="taxon:10407"
FT                   /db_xref="HBVdb:AF121240"
FT                   /PRABI_genotype="n.a.:D:n.a."
FT   CDS             1814..2452
FT                   /PRABI_name="PreC"
FT                   /locus_tag="HBVORF02"
FT                   /note="non-functional"
FT   CDS             1901..2452
FT                   /PRABI_name="C"
FT                   /locus_tag="HBVORF12"
FT                   /codon_start="1"
FT                   /translation="MDIDPYKEFGATVELLSFLPSDFFPSVRDLLDTASALYREALESP
FT                   EHCSPHHTALRQAILCWGDLMTLATWVGGNLQDPTSRDLVVSYVNTHMGLKFRQLLWFH
FT                   ISCLTFGRETVIEYLVSFGVWIRTPQAYRPPNAPILSTLPETTVVRRRGRTPRRRTPSP
FT                   RRRRSQSPRRRRSQSRESQC"
FT   mat_peptide     1901..2449
FT                   /function="Core protein coding sequence"
FT                   /locus_tag="HBVORF12"
FT                   /product="Core protein"
FT                   /PRABI_name="HBc"
FT                   /PRABI_prodft=(pos:1..183, CHAIN, "Core protein")
FT   CDS             1374..1838
FT                   /PRABI_name="X"
FT                   /locus_tag="HBVORF03"
FT                   /codon_start="1"
FT                   /translation="MAARLCCQLDPARDVLCLRPVGAESRGRPFPGSLGTLSSPTPSAV
FT                   STDHGAHLSLRGLPVCAFSSAGPCALRFTSARRMETTVNAHHFLPKVLYKRTLGLSVMS
FT                   TTDLEAYFKDCLFKDWEELGEEIRLMVYVLGGCRHKLVCAPAPCNFFTSA"
FT   mat_peptide     1374..1835
FT                   /function="X protein coding sequence"
FT                   /locus_tag="HBVORF03"
FT                   /product="X protein"
FT                   /PRABI_name="HBx"
```

```
FT                   /PRABI_prodft=(pos:1..154, CHAIN, "X protein")
FT   CDS             join(2848..3182,1..835)
FT                   /PRABI_name="PreS1"
FT                   /locus_tag="HBVORF01"
FT                   /codon_start="1"
FT                   /translation="MGQNLSTSNPLGFFPDHQLDPAFRANTANPDWDFNPNKDTWPDAN
FT                   KVGAGAFGLGFTPPHGGLLGWSPQAQGILQTLPANPPPASTNRQSGRQPTPLSPPLRDT
FT                   HPQAMQWNSTTFHQTLQDPRVRGLYFPAGGSSSGTVNPVPTTVSHISSIFSRIGGPALN
FT                   MENITSGFLRPLLVLQAGFSLLTKILTIPKSLDSWWTSLNFLGGTTVCLGQNSQSPTSN
FT                   HSPTSCPPTCPGYRWMCLRRFIIFLFILLLCLIFLLVLLDYQGMLPVCPLIPGSSTTST
FT                   GPCRTCTTPAQGTSMYPSCCCTKPSDGNCTCIPIPSSWAFGKFLWEWASARFSWLSLLV
FT                   PFVQWFVGLSPTVWLLVIWMMWYWGRSLYSILSPFLPLLPIFFCLWVYI"
FT   mat_peptide     join(2848..3182,1..832)
FT                   /function="PreS1/Large Surface protein coding sequence"
FT                   /locus_tag="HBVORF01"
FT                   /product="PreS1 Surface protein"
FT                   /PRABI_name="LHBs"
FT                   /PRABI_prodft=(pos:1..389, CHAIN, "Large Surface protein")
FT   CDS             join(3172..3182,1..835)
FT                   /PRABI_name="PreS2"
FT                   /locus_tag="HBVORF11"
FT                   /codon_start="1"
FT                   /translation="MQWNSTTFHQTLQDPRVRGLYFPAGGSSSGTVNPVPTTVSHISSI
FT                   FSRIGGPALNMENITSGFLRPLLVLQAGFSLLTKILTIPKSLDSWWTSLNFLGGTTVCL
FT                   GQNSQSPTSNHSPTSCPPTCPGYRWMCLRRFIIFLFILLLCLIFLLVLLDYQGMLPVCP
FT                   LIPGSSTTSTGPCRTCTTPAQGTSMYPSCCCTKPSDGNCTCIPIPSSWAFGKFLWEWAS
FT                   ARFSWLSLLVPFVQWFVGLSPTVWLLVIWMMWYWGRSLYSILSPFLPLLPIFFCLWVYI
FT                   "
FT   mat_peptide     join(3172..3182,1..832)
FT                   /function="PreS2/Middle Surface protein coding sequence"
FT                   /locus_tag="HBVORF11"
FT                   /product="PreS2 Surface protein"
FT                   /PRABI_name="MHBs"
FT                   /PRABI_prodft=(pos:1..281, CHAIN, "Middle Surface protein")
FT   CDS             155..835
FT                   /PRABI_name="S"
FT                   /locus_tag="HBVORF21"
FT                   /codon_start="1"
FT                   /translation="MENITSGFLRPLLVLQAGFSLLTKILTIPKSLDSWWTSLNFLGGT
FT                   TVCLGQNSQSPTSNHSPTSCPPTCPGYRWMCLRRFIIFLFILLLCLIFLLVLLDYQGML
FT                   PVCPLIPGSSTTSTGPCRTCTTPAQGTSMYPSCCCTKPSDGNCTCIPIPSSWAFGKFLW
FT                   EWASARFSWLSLLVPFVQWFVGLSPTVWLLVIWMMWYWGRSLYSILSPFLPLLPIFFCL
FT                   WVYI"
FT   mat_peptide     155..832
FT                   /function="S protein coding sequence"
FT                   /locus_tag="HBVORF21"
FT                   /product="Surface protein S"
FT                   /PRABI_name="SHBs"
FT                   /PRABI_prodft=(pos:1..226, CHAIN, "Small Surface protein")
FT   CDS             join(2307..3182,1..1623)
```

```
FT                   /PRABI_name="P"
FT                   /locus_tag="HBVORF13"
FT                   /codon_start="1"
FT                   /translation="MPLSYQRFRRLLLLDEEAGPLEEELPRLADEGLNRRVAEDLNLGN
FT                   LNVSIPWTHKVGNFTGLYSSTVPVFNPHWKTPSFPNIHLHQDIIKKCEQFVGPLTVNEK
FT                   RRLQLIMPARFYPNVTKYLPLDKGIKPYYPEHLVNHYFQTRHYLHTLWKAGILYKRETT
FT                   RSASFCGSPYSWEQELQHGAESFHQQSSGILSRPPVGSSLQSKHRKSRLGLQSQQGHLA
FT                   RCQQGRSWSIRAGIHPTARRPFGVEPSGSGHTTNLASKSASCLYQSPVRKAAYPSVSTF
FT                   ERHSSSGHAVELHNLPPNSARSQSERPVFPCWWLQFRNSKPCSDYCLSHIVNLLEDWGP
FT                   CAEHGEHHIRIPKTPARVTGGVFLVDKNPHNTEESRLVVDFSQFSRGNYRVSWPKFAVP
FT                   NLQSLTNLLSSNLSWLSLDVSAAFYHLPLHPAAMPHLLVGSSGLSRYVARLSSNSRIFN
FT                   HQHGTLQDLHDSCSRNLYVSLLLLYQTFGRKLHLYSHPIILGFRKIPMGVGLSPFLLAQ
FT                   FTSAICSVVRRAFPHCLAFSYMDDVVLGAKSVQHLESLFTAVTNFLLSLGIHLNPNKTK
FT                   RWGYSLHFMGYVIGCYGSLPQDHIIQKIKECFRKLPVNRPIDWKVCQRIVGLLGFAAPF
FT                   TQCGYPALMPLYACIQSKQAFTFSPTYKAFLCKQYLNLYPVARQRPGLCQVFADATPTG
FT                   WGLVMGHQRMRGTFRAPLPIHTAELLAACFARSRSGANILGTDNSVVLSRKYTSFPWLL
FT                   GCAANWILRGTSFVYVPSALNPADDPSRGRLGLSRPLLRLPFRPTTGRTSLYADSPSVP
FT                   SHLPDRVHFASPLHVAWRPP"
FT   mat_peptide     join(2307..3182,1..1620)
FT                   /function="DNA-polymerase/Reverse Transcriptase coding
FT                   sequence"
FT                   /locus_tag="HBVORF13"
FT                   /product="Polymerase/Reverse Transcriptase"
FT                   /PRABI_name="Pol"
FT                   /PRABI_prodft=(pos:1..832, CHAIN, "Polymerase/Reverse
FT                   transcriptase")
FT                   /PRABI_prodft=(pos:1..180, DOMAIN, "Terminal Protein (TP)/
FT                   Primase domain")
FT                   /PRABI_prodft=(pos:181..335, DOMAIN, "Spacer")
FT                   /PRABI_prodft=(pos:336..679, DOMAIN, "Reverse Transcriptase
FT                   (RT) domain")
FT                   /PRABI_prodft=(pos:418..418, ACT_SITE, "RT catalytic Asp")
FT                   /PRABI_prodft=(pos:540..540, ACT_SITE, "RT catalytic Asp")
FT                   /PRABI_prodft=(pos:541..541, ACT_SITE, "RT catalytic Asp")
FT                   /PRABI_prodft=(pos:680..832, DOMAIN, "Ribonuclease H
FT                   (RNaseH) domain")
FT                   /PRABI_prodft=(pos:689..689, ACT_SITE, "RNaseH catalytic
FT                   Asp")
FT                   /PRABI_prodft=(pos:718..718, ACT_SITE, "RNaseH catalytic
FT                   Glu")
FT                   /PRABI_prodft=(pos:737..737, ACT_SITE, "RNaseH catalytic
FT                   Asp")
FT   CDS             join(2307..2447,489..683)
FT                   /PRABI_name="SP"
FT                   /locus_tag="HBVORF04"
FT                   /codon_start="1"
FT                   /translation="MPLSYQRFRRLLLLDEEAGPLEEELPRLADEGLNRRVAEDLNLGN
FT                   LNDLQPPARDPAGPARLLLKEPLCIPPVAVPNLRTEIAPVFPSHHPGLSENSYGSGPQP
FT                   VSPGSVY"
FT   mat_peptide     join(2307..2447,489..680)
```

```
FT                   /function="HBSP coding sequence"
FT                   /locus_tag="HBVORF04"
FT                   /product="Hepatitis B Spliced Protein"
FT                   /PRABI_name="HBSP"
FT                   /PRABI_prodft=(pos:1..111, CHAIN, "HBV Spliced Protein")
XX
SQ   Sequence 3182 BP; 725 A; 852 C; 695 G; 910 T; 0 other;
     ctccacaacc ttccaccaaa ctctgcaaga tcccagagtg agaggcctgt atttccctgc        60
     tggtggctcc agttcaggaa cagtaaaccc tgttccgact actgtctctc acatatcgtc       120
     aatcttctcg aggattgggg gccctgcgct gaacatggag aacatcacat caggattcct       180
     aagacccctg ctcgtgttac aggcggggtt ttcttgttg acaaaaatcc tcacaatacc       240
     gaagagtcta gactcgtggt ggacttctct caattttcta gggggaacta ccgtgtgtct       300
     tggccaaaat tcgcagtccc caacctccaa tcactcacca acctcctgtc ctccaacttg       360
     tcctggttat cgctggatgt gtctgcggcg ttttatcatc ttcctcttca tcctgctgct       420
     atgcctcatc ttcttgttgg ttcttctgga ctatcaaggt atgttgcccg tttgtcctct       480
     aattccagga tcttcaacca ccagcacggg accctgcagg acctgcacga ctcctgctca       540
     aggaacctct atgtatccct cctgttgctg taccaaacct tcggacggaa attgcacctg       600
     tattcccatc ccatcatcct gggctttcgg aaaattccta tgggagtggg cctcagcccg       660
     tttctcctgg ctcagtttac tagtgccatt tgttcagtgg ttcgtagggc tttcccccac       720
     tgtttggctt ttagttatat ggatgatgtg gtattggggg cgaagtctgt acagcatctt       780
     gagtcccttt ttaccgctgt taccaatttt cttttgtctt tgggtataca tttaaatcct       840
     aacaaaacaa aaagatgggg ttactcttta catttcatgg gctatgtcat tggatgttat       900
     gggtccttgc cacaagatca catcatacaa aaaatcaaag aatgttttag gaaacttcct       960
     gttaacaggc ctattgattg gaaagtctgt caacgtattg tgggtctttt gggttttgct      1020
     gccccttta cacaatgtgg ttatcctgct ttaatgccct tgtatgcatg tattcaatct      1080
     aagcaggctt tcactttctc gccaacttac aaggcctttc tgtgtaaaca atacctgaac      1140
     ctttaccccg ttgcccggca acggccaggt ctgtgccaag tgtttgctga cgcaaccccc      1200
     actggctggg gcttggtcat gggccatcag cgcatgcgtg gaacctttcg ggctcctctg      1260
     ccgatccata ctgcggaact cctagccgct tgttttgctc gcagcaggtc tggagcaaac      1320
     attctcggga cggataactc tgttgttctc tcccgcaaat atacatcgtt tccatggctg      1380
     ctaggctgtg ctgccaactg gatcctgcgc gggacgtcct ttgtttacgt cccgtcggcg      1440
     ctgaatcccg cggacgaccc ttcccggggt cgcttgggac tctctcgtcc cctactccgt      1500
     ctgccgtttc gaccgaccac ggggcgcacc tctctttacg cggactcccc gtctgtgcct      1560
     tctcatctgc cggaccgtgt gcacttcgct tcacctctgc acgtcgcatg gagaccaccg      1620
     tgaacgccca ccacttcttg cccaaggtct tatataagag gactcttgga ctctctgtaa      1680
     tgtcaacgac cgaccttgag gcatacttca aagactgttt gtttaaagac tgggaggagt      1740
     tgggggagga gattagatta atggtttatg tattaggagg ctgtaggcat aaattggtgt      1800
     gcgcaccagc accatgcaac tttttcacct ctgcctaatc atctcttgtt catgtcctac      1860
     tgttcaagcc tccaagctgt gccttgggtg gctttaggac atggacattg atccttataa      1920
     agaatttgga gctactgtgg agttactctc gtttttgcct tctgacttct ttccttccgt      1980
     acgagatctt ctagataccg cctcagcttt atatcgggaa gccttagagt ctcctgagca      2040
     ttgttcacct caccatacgg cactcaggca agcaattctt tgctgggggg atttaatgac      2100
     tctagccacc tgggtgggtg gtaatttgca ggatccaaca tccagggacc tagtagttag      2160
     ttatgttaac actcatatgg gcctaaaatt caggcaacta ttgtggtttc acatttcctg      2220
     tctcactttt ggaagagaaa cggtcataga gtatttggtg tctttcggag tgtggattcg      2280
     cactcctcaa gcttatagac caccaaatgc ccctatctta tcaacgcttc cggagactac      2340
     tgttgttaga cgaagaggca ggacccctag aagaagaact ccctcgcctc gcagacgaag      2400
     gtctcaatcg ccgcgtcgca gaagatctca atctcgggaa tctcaatgtt agtattcctt      2460
     ggactcataa ggtgggaaac tttacggggc tttattcttc tactgtgcct gtctttaacc      2520
     ctcattggaa aacaccctct ttttcctaata tacatttaca ccaagatatt atcaaaaaat      2580
```

```
     gtgaacaatt tgtaggccca cttacagtca atgagaaaag aagactgcaa ttgattatgc      2640
     ctgctaggtt ttatccaaat gttaccaaat atttgccatt ggataagggt attaaacctt      2700
     attatccaga acatctagtt aatcattact tccaaaccag acattattta cacactctat      2760
     ggaaggcggg tatattatat aagagagaaa caacacgtag cgcctcattt tgtgggtcac      2820
     catattcttg ggaacaagag ctacagcatg gggcagaatc tttccaccag caatcctctg      2880
     ggattctttc ccgaccacca gttggatcca gccttcagag caaacaccgc aaatccagat      2940
     tgggacttca atcccaacaa ggacacctgg ccagatgcca acaaggtagg agctggagca      3000
     ttcgggctgg gattcacccc accgcacggc ggccttttgg ggtggagccc tcaggctcag      3060
     ggcatactac aaaccttgcc agcaaatccg cctcctgcct ctaccaatcg ccagtcagga      3120
     aggcagccta cccctctgtc tccacctttg agagacactc atcctcaggc catgcagtgg      3180
     aa                                                                     3182
//
```

© 1998-2022 Centre de Recherche en Cancerologie de Lyon logo <http://www.crcl.fr/> Pole Rhone-Alpes de BioInformatique logo <http://prabi.ibcp.fr/> Centre National de la Recherche Scientifique logo <http://www.cnrs.fr/> Institut national de la sante et de la recherche medicale logo <http://www.inserm.fr/> Universite Claude Bernard Lyon 1 logo <http://www.univ-lyon1.fr/> Legal notice <https://hbvdb.lyon.inserm.fr/HBVdb/HBVdbAbout#legalnotice>
