# Supplementary material for: Full-Length Genome Sequencing and Analysis of Hepatitis B Viruses Isolated from Iraqi Patients
Source: Int J Microbiol. 2024 Apr 29;2024:6826495. doi: 10.1155/2024/6826495 (PMC11074772; doi:10.1155/2024/6826495)
Supplement: Supplementary Materials — The metadata of HBV samples is shown in S1, Supplementary Materials, as a PDF file. Alignment of the obtained nucleotide sequences (OM721310–OM721316) to the reference sequence (NC_003977) in the NCBI GenBank database using the Clustal W method integrated into the SnapGene software is shown in S2, Supplementary Materials, as a PDF file. Determination of genotypes of HBV isolates (OM721310–OM721316) using the annotation algorithm in the HBVdb is shown in S3, Supplementary Materials, as a PDF file. Determination of genotypes, subgenotypes of HBV isolates (OM721310–OM721316), and their sensitivity to antiviral drugs using Geno2pheno are shown in S4, Supplementary Materials, as PDF files. Serotype determination of HBV isolates (OM721310–OM721316) using the HBV Serotyper tool is shown in S5, Supplementary Materials, as a PDF file. [file 6826495.f1.zip › S4. Determination of genotypes, sub genotypes of HBV isolates (OM721310-OM721316) and their sensitivity to antiviral drugs (1).pdf]

geno2pheno®

HBV resistance prediction from genotype (version 2.0)

S4. Determination of genotypes, sub genotypes of HBV isolates (OM721310-OM721316) and their sensitivity to antiviral drugs using Geno2pheno

Sequence Information		Resistance Prediction	
Sample ID:	OM721310 - S1	Lamivudine:	susceptible
Genotype:	D (D1)	Adefovir:	susceptible
RT Mutations:	Y135S, N248H	Entecavir:	susceptible
SHB Mutations:	N3S, T127P	Tenofovir:	susceptible
Escape Mutations:		Telbivudine:	susceptible

Comments:

____
(signature)

geno2pheno®

HBV resistance prediction from genotype (version 2.0)

Sequence Information		Resistance Prediction	
Sample ID:	OM721311 - S2	Lamivudine:	susceptible
Genotype:	D (D1)	Adefovir:	susceptible
RT Mutations:	Y135S, N248H	Entecavir:	susceptible
SHB Mutations:	N3S, L42R, T127P, L175S	Tenofovir:	susceptible
Escape Mutations:		Telbivudine:	susceptible

Comments:

(signature)

Contact: info@mpi-inf.mpg.de
www.geno2pheno.org

geno2pheno®

HBV resistance prediction from genotype (version 2.0)

Sequence Information		Resistance Prediction	
Sample ID:	OM721312 - S3	Lamivudine:	susceptible
Genotype:	D (D1)	Adefovir:	susceptible
RT Mutations:	Y135S, L220I, N248H, Q319L	Entecavir:	susceptible
SHB Mutations:	T127P	Tenofovir:	susceptible
Escape Mutations:		Telbivudine:	susceptible

Comments:

(signature)

Contact: info@mpi-inf.mpg.de
www.geno2pheno.org

geno2pheno®

HBV resistance prediction from genotype (version 2.0)

Sequence Information		Resistance Prediction	
Sample ID:	OM721313 - S4	Lamivudine:	susceptible
Genotype:	D (D1)	Adefovir:	susceptible
RT Mutations:	Y135S, L220I, N248H, Q319L	Entecavir:	susceptible
SHB Mutations:	T127P	Tenofovir:	susceptible
Escape Mutations:		Telbivudine:	susceptible

Comments:

(signature)

Contact: info@mpi-inf.mpg.de
www.geno2pheno.org

geno2pheno®

HBV resistance prediction from genotype (version 2.0)

Sequence Information		Resistance Prediction	
Sample ID:	OM721314 - S5	Lamivudine:	susceptible
Genotype:	D (D1)	Adefovir:	susceptible
RT Mutations:	Y135S, L220I, N248H, Q319L	Entecavir:	susceptible
SHB Mutations:	T127P	Tenofovir:	susceptible
Escape Mutations:		Telbivudine:	susceptible

Comments:

(signature)

geno2pheno®

HBV resistance prediction from genotype (version 2.0)

Sequence Information		Resistance Prediction	
Sample ID:	OM721315 - S6	Lamivudine:	susceptible
Genotype:	D (D1)	Adefovir:	susceptible
RT Mutations:	F122V, T128P, Y135S, N248H, V278I	Entecavir:	susceptible
SHB Mutations:	T127P, L175S	Tenofovir:	susceptible
Escape Mutations:		Telbivudine:	susceptible

Comments:

(signature)

Contact: info@mpi-inf.mpg.de
www.geno2pheno.org

geno2pheno®

HBV resistance prediction from genotype (version 2.0)

Sequence Information		Resistance Prediction	
Sample ID:	OM721316 - S7	Lamivudine:	susceptible
Genotype:	D (D1)	Adefovir:	susceptible
RT Mutations:	R18K, A38E, M129L, N131D, Y135S, N248H	Entecavir:	susceptible
SHB Mutations:	G10R, F20S, R24K, Q30K, T127P, S193L, P203R	Tenofovir:	susceptible
Escape Mutations:		Telbivudine:	susceptible

Comments:

(signature)

Contact: info@mpi-inf.mpg.de
www.geno2pheno.org
