# Supplementary material for: Full-Length Genome Sequencing and Analysis of Hepatitis B Viruses Isolated from Iraqi Patients
Source: Int J Microbiol. 2024 Apr 29;2024:6826495. doi: 10.1155/2024/6826495 (PMC11074772; doi:10.1155/2024/6826495)
Supplement: Supplementary Materials — The metadata of HBV samples is shown in S1, Supplementary Materials, as a PDF file. Alignment of the obtained nucleotide sequences (OM721310–OM721316) to the reference sequence (NC_003977) in the NCBI GenBank database using the Clustal W method integrated into the SnapGene software is shown in S2, Supplementary Materials, as a PDF file. Determination of genotypes of HBV isolates (OM721310–OM721316) using the annotation algorithm in the HBVdb is shown in S3, Supplementary Materials, as a PDF file. Determination of genotypes, subgenotypes of HBV isolates (OM721310–OM721316), and their sensitivity to antiviral drugs using Geno2pheno are shown in S4, Supplementary Materials, as PDF files. Serotype determination of HBV isolates (OM721310–OM721316) using the HBV Serotyper tool is shown in S5, Supplementary Materials, as a PDF file. [file 6826495.f1.zip › S5. Serotype determination of HBV isolates (OM721310-OM721316) using the HBV Serotyper tool (1).pdf]

S5. Serotype determination of HBV isolates (OM721310-OM721316) using the HBV Serotyper tool

"Purdy" HBV Serotyper Results

Input file: HBsAg (OM721310).fa

S Region Start Motif: ATGGAGAGCAT

The "Motif1" column shows the five amino acids (122, 160, 127, 159 and 140) required for serotyping. All five amino acids are not required to determine all serotypes, but all five are shown for each sequence. The "Motif3" column shows the three-letter abbreviations for the amino acid motif. The "Motif Sequence" shows the nucleotide sequence for each of the five amino acids for reference (positions 366-368, 480-482, 381-383, 477-479 and 420-422). Serotypes which cannot be determined are shown as "Unknown". A serotype of "(ad)" or "(ay)" indicates that only the /d/ or /y/ determinant class could be deduced. A summary table of the amino acids required for each serotype appears below the results.

The serotype is determined as per Figure 3 in Purdy, M. A., Talekar, G., Swenson, P., Araujo, A. and Fields, H. 2007. A new algorithm for deduction of hepatitis B virus surface antigen subtype determinants from the amino acid sequence. /Intervirology/ *50*: 45-51. DOI: 10.1159/000096312.

Sequence ID	*Serotype*	*Motif1*	*Motif3*	*Motif Sequence*
HBsAg (S) (681 bp)	ayw2	RKPGT	ArgLysProGlyThr	AGAAAACCTGGAACC

	122	160	127	159	140
adr	K	R			
adw2	K	K	P		
adw3	K	K	T		
adw4	K	K	I or L		
ayr	R	R			
ayw3	R	K	T		
ayw4	R	K	I or L		
ayw1	R	K	P	A	
ayw2	R	K	P	Not A	Not S
ayw4	R	K	P	Not A	S

Version 0.1 Beta (July 2010)
Request from 37.237.176.54
Served by hvdr.bioinf.wits.ac.za at 146.141.240.41
Run completed 2022-02-11 15:42
0.151210 seconds

"Purdy" HBV Serotyper Results

Input file: HBsAg (S).OM721311.fa

S Region Start Motif: ATGGAGAGCAT

The "Motif1" column shows the five amino acids (122, 160, 127, 159 and 140) required for serotyping. All five amino acids are not required to determine all serotypes, but all five are shown for each sequence. The "Motif3" column shows the three-letter abbreviations for the amino acid motif. The "Motif Sequence" shows the nucleotide sequence for each of the five amino acids for reference (positions 366-368, 480-482, 381-383, 477-479 and 420-422). Serotypes which cannot be determined are shown as "Unknown". A serotype of "(ad)" or "(ay)" indicates that only the /d/ or /y/ determinant class could be deduced. A summary table of the amino acids required for each serotype appears below the results.

The serotype is determined as per Figure 3 in Purdy, M. A., Talekar, G., Swenson, P., Araujo, A. and Fields, H. 2007. A new algorithm for deduction of hepatitis B virus surface antigen subtype determinants from the amino acid sequence. /Intervirology/ *50*: 45-51. DOI: 10.1159/000096312.

Sequence ID	*Serotype*	*Motif1*	*Motif3*	*Motif Sequence*
HBsAg (S) (681 bp)	ayw2	RKPGT	ArgLysProGlyThr	AGAAAACCTGGAACC

	122	160	127	159	140
adr	K	R			
adw2	K	K	P		
adw3	K	K	T		
adw4	K	K	I or L		
ayr	R	R			
ayw3	R	K	T		
ayw4	R	K	I or L		
ayw1	R	K	P	A	
ayw2	R	K	P	Not A	Not S
ayw4	R	K	P	Not A	S

Version 0.1 Beta (July 2010)
Request from 37.237.176.67
Served by hvdr.bioinf.wits.ac.za at 146.141.240.41
Run completed 2022-02-11 15:54
0.145867 seconds

"Purdy" HBV Serotyper Results

Input file: HBsAg (S)-OM721312.fa

S Region Start Motif: ATGGAGAACAT

The "Motif1" column shows the five amino acids (122, 160, 127, 159 and 140) required for serotyping. All five amino acids are not required to determine all serotypes, but all five are shown for each sequence. The "Motif3" column shows the three-letter abbreviations for the amino acid motif. The "Motif Sequence" shows the nucleotide sequence for each of the five amino acids for reference (positions 366-368, 480-482, 381-383, 477-479 and 420-422). Serotypes which cannot be determined are shown as "Unknown". A serotype of "(ad)" or "(ay)" indicates that only the /d/ or /y/ determinant class could be deduced. A summary table of the amino acids required for each serotype appears below the results.

The serotype is determined as per Figure 3 in Purdy, M. A., Talekar, G., Swenson, P., Araujo, A. and Fields, H. 2007. A new algorithm for deduction of hepatitis B virus surface antigen subtype determinants from the amino acid sequence. /Intervirology/ *50*: 45-51. DOI: 10.1159/000096312.

Sequence ID	*Serotype*	*Motif1*	*Motif3*	*Motif Sequence*
HBsAg (S) (681 bp)	ayw2	RKPGT	ArgLysProGlyThr	AGAAAACCTGGAACC

	122	160	127	159	140
adr	K	R			
adw2	K	K	P		
adw3	K	K	T		
adw4	K	K	I or L		
ayr	R	R			
ayw3	R	K	T		
ayw4	R	K	I or L		
ayw1	R	K	P	A	
ayw2	R	K	P	Not A	Not S
ayw4	R	K	P	Not A	S

Version 0.1 Beta (July 2010)
Request from 37.237.176.67
Served by hvdr.bioinf.wits.ac.za at 146.141.240.41
Run completed 2022-02-11 15:56
0.131097 seconds

"Purdy" HBV Serotyper Results

Input file: HBsAg (S)-OM721313.fa

S Region Start Motif: ATGGAGAACAT

The "Motif1" column shows the five amino acids (122, 160, 127, 159 and 140) required for serotyping. All five amino acids are not required to determine all serotypes, but all five are shown for each sequence. The "Motif3" column shows the three-letter abbreviations for the amino acid motif. The "Motif Sequence" shows the nucleotide sequence for each of the five amino acids for reference (positions 366-368, 480-482, 381-383, 477-479 and 420-422). Serotypes which cannot be determined are shown as "Unknown". A serotype of "(ad)" or "(ay)" indicates that only the /d/ or /y/ determinant class could be deduced. A summary table of the amino acids required for each serotype appears below the results.

The serotype is determined as per Figure 3 in Purdy, M. A., Talekar, G., Swenson, P., Araujo, A. and Fields, H. 2007. A new algorithm for deduction of hepatitis B virus surface antigen subtype determinants from the amino acid sequence. /Intervirology/ *50*: 45-51. DOI: 10.1159/000096312.

Sequence ID	*Serotype*	*Motif1*	*Motif3*	*Motif Sequence*
HBsAg (S) (681 bp)	ayw2	RKPGT	ArgLysProGlyThr	AGAAAACCTGGAACC

	122	160	127	159	140
adr	K	R			
adw2	K	K	P		
adw3	K	K	T		
adw4	K	K	I or L		
ayr	R	R			
ayw3	R	K	T		
ayw4	R	K	I or L		
ayw1	R	K	P	A	
ayw2	R	K	P	Not A	Not S
ayw4	R	K	P	Not A	S

Version 0.1 Beta (July 2010)
Request from 37.237.176.67
Served by hvdr.bioinf.wits.ac.za at 146.141.240.41
Run completed 2022-02-11 15:58
0.133774 seconds

"Purdy" HBV Serotyper Results

Input file: HBsAg (S)-OM721314.fa

S Region Start Motif: ATGGAGAACAT

The "Motif1" column shows the five amino acids (122, 160, 127, 159 and 140) required for serotyping. All five amino acids are not required to determine all serotypes, but all five are shown for each sequence. The "Motif3" column shows the three-letter abbreviations for the amino acid motif. The "Motif Sequence" shows the nucleotide sequence for each of the five amino acids for reference (positions 366-368, 480-482, 381-383, 477-479 and 420-422). Serotypes which cannot be determined are shown as "Unknown". A serotype of "(ad)" or "(ay)" indicates that only the /d/ or /y/ determinant class could be deduced. A summary table of the amino acids required for each serotype appears below the results.

The serotype is determined as per Figure 3 in Purdy, M. A., Talekar, G., Swenson, P., Araujo, A. and Fields, H. 2007. A new algorithm for deduction of hepatitis B virus surface antigen subtype determinants from the amino acid sequence. /Intervirology/ *50*: 45-51. DOI: 10.1159/000096312.

Sequence ID	*Serotype*	*Motif1*	*Motif3*	*Motif Sequence*
HBsAg (S) (681 bp)	ayw2	RKPGT	ArgLysProGlyThr	AGAAAACCTGGAACC

	122	160	127	159	140
adr	K	R			
adw2	K	K	P		
adw3	K	K	T		
adw4	K	K	I or L		
ayr	R	R			
ayw3	R	K	T		
ayw4	R	K	I or L		
ayw1	R	K	P	A	
ayw2	R	K	P	Not A	Not S
ayw4	R	K	P	Not A	S

Version 0.1 Beta (July 2010)
Request from 37.237.176.67
Served by hvdr.bioinf.wits.ac.za at 146.141.240.41
Run completed 2022-02-11 16:01
0.133073 seconds

"Purdy" HBV Serotyper Results

Input file: HBsAg (S)-OM721315.fa

S Region Start Motif: ATGGAGAACAT

The "Motif1" column shows the five amino acids (122, 160, 127, 159 and 140) required for serotyping. All five amino acids are not required to determine all serotypes, but all five are shown for each sequence. The "Motif3" column shows the three-letter abbreviations for the amino acid motif. The "Motif Sequence" shows the nucleotide sequence for each of the five amino acids for reference (positions 366-368, 480-482, 381-383, 477-479 and 420-422). Serotypes which cannot be determined are shown as "Unknown". A serotype of "(ad)" or "(ay)" indicates that only the /d/ or /y/ determinant class could be deduced. A summary table of the amino acids required for each serotype appears below the results.

The serotype is determined as per Figure 3 in Purdy, M. A., Talekar, G., Swenson, P., Araujo, A. and Fields, H. 2007. A new algorithm for deduction of hepatitis B virus surface antigen subtype determinants from the amino acid sequence. /Intervirology/ *50*: 45-51. DOI: 10.1159/000096312.

Sequence ID	*Serotype*	*Motif1*	*Motif3*	*Motif Sequence*
HBsAg (S) (681 bp)	ayw2	RKPGT	ArgLysProGlyThr	AGAAAACCTGGAACC

	122	160	127	159	140
adr	K	R			
adw2	K	K	P		
adw3	K	K	T		
adw4	K	K	I or L		
ayr	R	R			
ayw3	R	K	T		
ayw4	R	K	I or L		
ayw1	R	K	P	A	
ayw2	R	K	P	Not A	Not S
ayw4	R	K	P	Not A	S

Version 0.1 Beta (July 2010)
Request from 37.237.176.67
Served by hvdr.bioinf.wits.ac.za at 146.141.240.41
Run completed 2022-02-11 16:02
0.126542 seconds

"Purdy" HBV Serotyper Results

Input file: HBsAg (S)-OM721316.fa

S Region Start Motif: ATGGAGAACAT

The "Motif1" column shows the five amino acids (122, 160, 127, 159 and 140) required for serotyping. All five amino acids are not required to determine all serotypes, but all five are shown for each sequence. The "Motif3" column shows the three-letter abbreviations for the amino acid motif. The "Motif Sequence" shows the nucleotide sequence for each of the five amino acids for reference (positions 366-368, 480-482, 381-383, 477-479 and 420-422). Serotypes which cannot be determined are shown as "Unknown". A serotype of "(ad)" or "(ay)" indicates that only the /d/ or /y/ determinant class could be deduced. A summary table of the amino acids required for each serotype appears below the results.

The serotype is determined as per Figure 3 in Purdy, M. A., Talekar, G., Swenson, P., Araujo, A. and Fields, H. 2007. A new algorithm for deduction of hepatitis B virus surface antigen subtype determinants from the amino acid sequence. /Intervirology/ *50*: 45-51. DOI: 10.1159/000096312.

Sequence ID	*Serotype*	*Motif1*	*Motif3*	*Motif Sequence*
HBsAg (S) (681 bp)	ayw2	RKPGT	ArgLysProGlyThr	AGGAAACCTGGAACC

	122	160	127	159	140
adr	K	R			
adw2	K	K	P		
adw3	K	K	T		
adw4	K	K	I or L		
ayr	R	R			
ayw3	R	K	T		
ayw4	R	K	I or L		
ayw1	R	K	P	A	
ayw2	R	K	P	Not A	Not S
ayw4	R	K	P	Not A	S

Version 0.1 Beta (July 2010)
Request from 37.237.176.67
Served by hvdr.bioinf.wits.ac.za at 146.141.240.41
Run completed 2022-02-11 16:05
0.132641 seconds
